# From Polyester Plastics to Diverse Monomers via Low‐Energy Upcycling

**DOI:** 10.1002/advs.202403002

**Published:** 2024-04-16

**Authors:** Lei Ji, Jiaolong Meng, Chengliang Li, Ming Wang, Xuefeng Jiang

**Affiliations:** ^1^ State Key Laboratory of Molecular & Process Engineering School of Chemistry and Molecular Engineering East China Normal University North Zhongshan Road 3663 Shanghai 200062 China; ^2^ School of Chemistry and Chemical Engineering Henan Normal University Xinxiang Henan 453007 China

**Keywords:** chemical upcycling, polyester plastics, polyethylene terephthalate, pre‐activation, trifluoroacetic acid

## Abstract

Polyester plastics, constituting over 10% of the total plastic production, are widely used in packaging, fiber, single‐use beverage bottles, etc. However, their current depolymerization processes face challenges such as non‐broad spectrum recyclability, lack of diversified high‐value‐added depolymerization products, and crucially high energy consumption. Herein, an efficient strategy is developed for dismantling the compact structure of polyester plastics to achieve diverse monomer recovery. Polyester plastics undergo swelling and decrystallization with a low depolymerization energy barrier via synergistic effects of polyfluorine/hydrogen bonding, which is further demonstrated via density functional theory calculations. The swelling process is elucidated through scanning electron microscopy analysis. Obvious destruction of the crystalline region is demonstrated through X‐ray crystal diffractometry curves. PET undergoes different aminolysis efficiently, yielding nine corresponding high‐value‐added monomers via low‐energy upcycling. Furthermore, four types of polyester plastics and five types of blended polyester plastics are closed‐loop recycled, affording diverse monomers with exceeding 90% yields. Kilogram‐scale depolymerization of real polyethylene terephthalate (PET) waste plastics is successfully achieved with a 96% yield.

## Graphical abstract

Polyester plastics undergo swelling and decrystallization with a low depolymerization energy barrier via synergistic effects of polyfluorine/hydrogen bonding by TFA. TFA‐treated polyester undergoes different aminolysis, yielding nine corresponding high‐added‐value chemicals. Furthermore, four polyester plastics and five blended polyester plastics are closed‐loop recycled efficiently, Kilogram‐scale depolymerization of real mixed PET waste plastics is achieved with 96% yield.

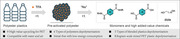

## 1 Introduction

**Scheme 1:**
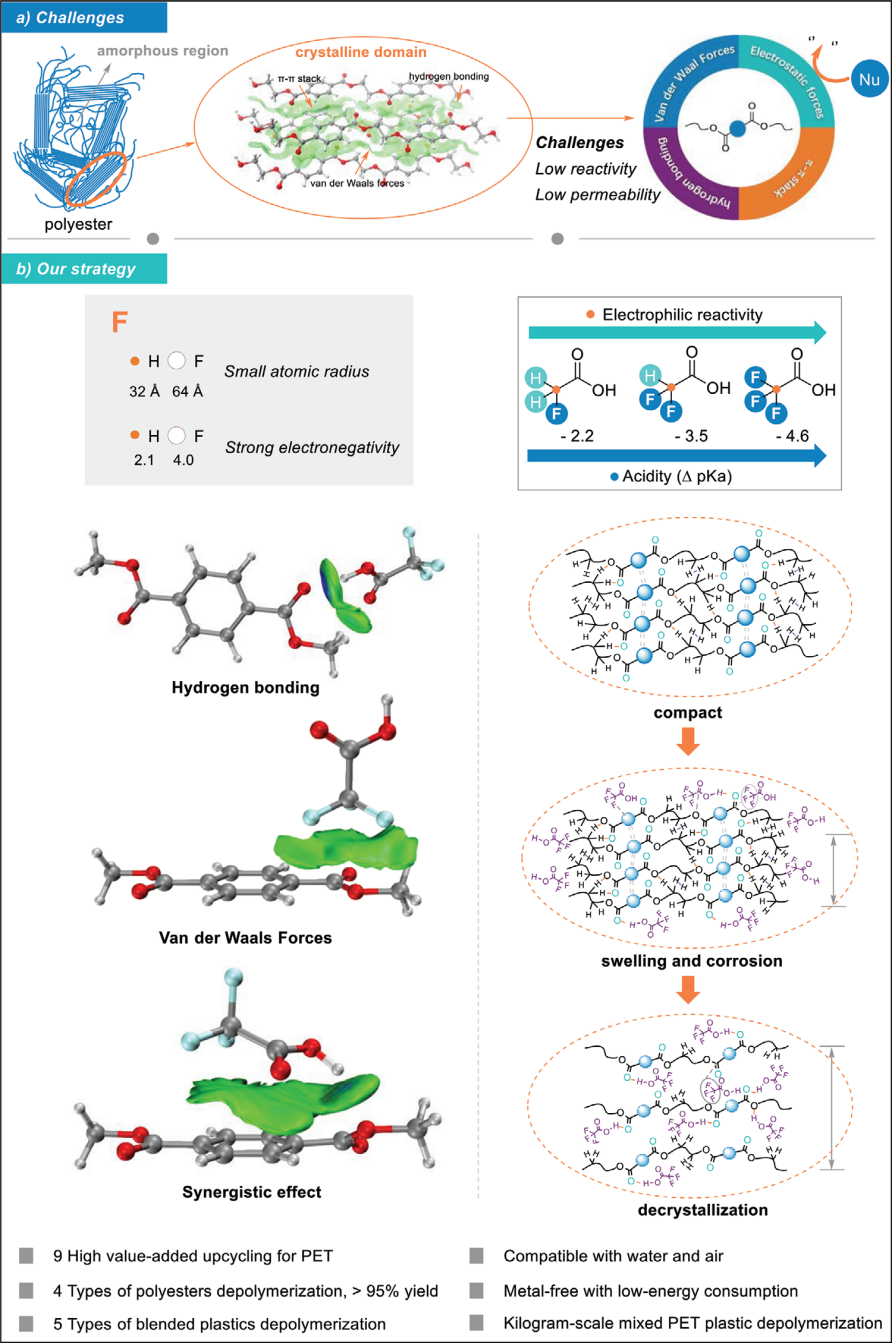
Strategies for depolymerization of polyesters. a) Challenges for depolymerization of semi‐crystalline polyesters; b) our strategy. Δ pKa (the pKa based on acetic acid is 0).

Polyesters are symmetrical and saturated linear polymers.^[^ ^1^ ^]^ Compared to their monomers and oligomers, polyesters exhibit excellent crystallinity and intense intra/intermolecular forces (such as van der Waals forces, π–π stack, hydrogen bonding, etc), which dramatically decrease the reactivity of the carbonyl group from ester posing the challenges for depolymerization under ambient conditions.^[^ ^2^ ^]^ Chemical depolymerization of polyester^[^ ^3^ ^]^ primarily includes hydrolysis,^[^ ^4^ ^]^ alcoholysis,^[^ ^5^ ^]^ and aminolysis,^[^ ^6^ ^]^ demanding the assistance of high temperature, high pressure, or stoichiometric metal additives (**Scheme** **1**a).^[^ ^7^ ^]^ Depolymerization for polyester is in great demand with an energy‐efficient process and diverse high‐value‐added monomer recovery for environmentally friendly recycling.^[^ ^8^ ^]^

Dissolution/reprecipitation is an effective pathway for recycling polymers.^[^ ^9^ ^]^ Higher solubility facilitates polymer chains swelling with stronger polymer/solvent interactions. Polymer preactivation can be achieved by disrupting the crystalline structure through polymer/solvent interaction. Manipulation of solvent solubility influences the morphology, crystallinity, and chain conformation of the polymer.^[^ ^10^, ^11^ ^]^ In 2019, Rafael Vazquez‐Duhalt employed the trifluoroacetic acid/water system to afford PET nanoparticles.^[^ ^12^ ^]^ In 2021, Mu employed the γ‐valerolactone/water system promoting hydrolysis for PET pretreatment at 120–170 °C.^[^ ^13^ ^]^

Due to the small atomic radius, low polarizability, and strong electronegativity of fluorine,^[^ ^14^ ^]^ polyfluorinated organic acids^[^ ^15^ ^]^ exhibit excellent lipophilicity and acidity^[^ ^16^ ^]^ enabling the strong interactions with polyester chains,^[^ ^17^ ^]^ thereby disrupting the condensed matter structure. Herein, a one‐pot strategy was employed utilizing the synergistic effect of fluorine and hydrogen bonding to pre‐activate polyester, facilitating the closed‐loop multifunctional upcycling of PET for diverse real polyester plastics (Scheme 1b).

## 2 Results and Discussion

**Figure 1:**
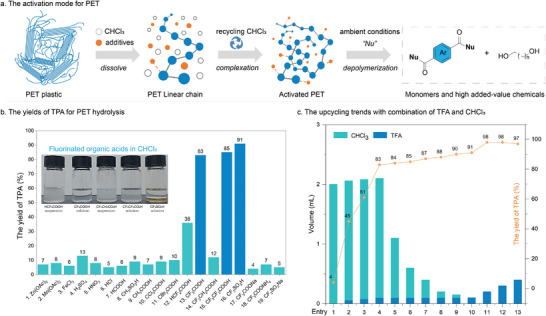
Depolymerization of PET. a) The activation mode for PET; b) the yields of TPA for PET hydrolysis; c) the upcycling trends with the combination of TFA and CHCl_3_.

Our study commenced with electrophilic fluorinated additives, disrupting the intermolecular forces between PET chains (**Figure** **1**a). After an extensive screening, the results revealed a significant advantage of fluorinated acids over other Lewis and Brønsted acids (Figure 1b). Considering economy and practicability, trifluoroacetic acid (TFA) was chosen as the best additive (Table S3, Supporting Information), leading to the efficient depolymerization for PET with 83% yield recovery of terephthalic acid (TPA) (Figure 1b, entry 13). TFA salts failed to depolymerize (entries 17–19), indicating that the acidity of TFA played a crucial role in the depolymerization. Subsequently, the ratio between chloroform (CHCl_3_) and TFA was further investigated (Figure 1c). The yield of TPA was correlated with the concentration of TFA. With the absence of CHCl_3_, 10 equivalents of TFA enable PET depolymerization to TPA up to 98% (Figure 1c, entry 11).

**Figure 2:**
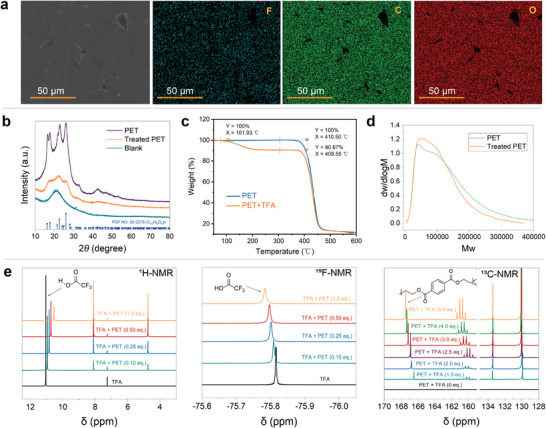
Characterization of activation and depolymerization. a) EDS studies; b) XRD curves; c) TGA curves; d) GPC curves; e) ^1^H NMR, ^19^F NMR (TFA was employed as the criteria), and 13C NMR studies (PET was employed as the criteria), see Supporting Information for details.

To acquire a comprehensive understanding of the depolymerization process, a series of characterizations were further conducted. In scanning electron microscopy (SEM) analysis, the surface texture of PET plastic was smooth and dense on a 5–10 µm scale, while TFA‐treated PET displayed a porous and loose structure (Figure S12, Supporting Information). Energy dispersion spectrometer (EDS) studies showed the uniform distribution of fluorine excluding TFA as a solvent residue, indicating that a complex was formed between PET plastic and TFA from the perspective of morphology (**Figure** **2**a). X‐ray crystal diffractometry (XRD) analysis revealed that the crystalline region of PET was disrupted (Figure 2b). The differential scanning calorimetry (DSC) curve of TFA‐treated PET shows an increased glass transition temperature (*T_g_*) from 85.6 °C for PET plastic to 111.2 °C (Figure S15, Supporting Information), consistent with the impact of hydrogen bonding interactions on *T_g_* of amorphous region in semi‐crystalline plastics.^[^ ^18^ ^]^ The thermal gravimetric analysis (TGA) curve indicates that TFA‐treated PET (depicted by the orange line) experiences a 9.3% mass loss at 101.9 °C and a mass loss similar to PET plastic at 408.6 °C (blue line), demonstrating that the first mass loss at 101.9 °C is TFA coated on PET chains. Moreover, TFA‐treated PET cannot be hydrolyzed after losing TFA at high temperatures. Thus, the existence of interactions between PET and TFA is a crucial factor enabling the depolymerization of the complex, in line with the reversibility of hydrogen bonding interactions (Figure 2c).^[^ ^19^ ^]^ Notably, a comparison of gel permeation chromatography (GPC) curves indicates no substantial reduction in molecular weight between TFA‐treated PET and the original PET, which reveals that TFA serves as an activator but not for depolymerization. (Figure 2d). Given the above, the pre‐activation process involved swelling and decrystallization of PET in TFA, followed by the removal of free TFA. A complex of PET with TFA was formed through intra/intermolecular forces, replacing the original intermolecular forces of PET chains.

From ^1^H NMR and ^19^F NMR studies, the characteristic chemical shifts of TFA were shifted with the concentration of TFA in chloroform‐*d_3_* increasing, while the chemical shift of ester carbonyl in PET shifted toward the low field in ^13^C NMR studies (Figure 2e). These phenomena suggested the interactions between hydrogen and fluorine atoms in TFA and ester carbonyl groups in PET chains. Specifically, TFA activated the ester carbonyl groups of PET chains, thereby increasing their polarization and electrophilicity. Fourier‐transform infrared spectroscopy (FT‐IR) analysis of the PET/TFA complex indicated a shift in the absorption peak of the carbonyl group from 1712.86 cm^−1^ in PET plastic to 1714.79 cm^−1^ (Figure S17, Supporting Information), supporting the TFA interaction with the ester carbonyl groups in PET/TFA complex.

**Figure 3:**
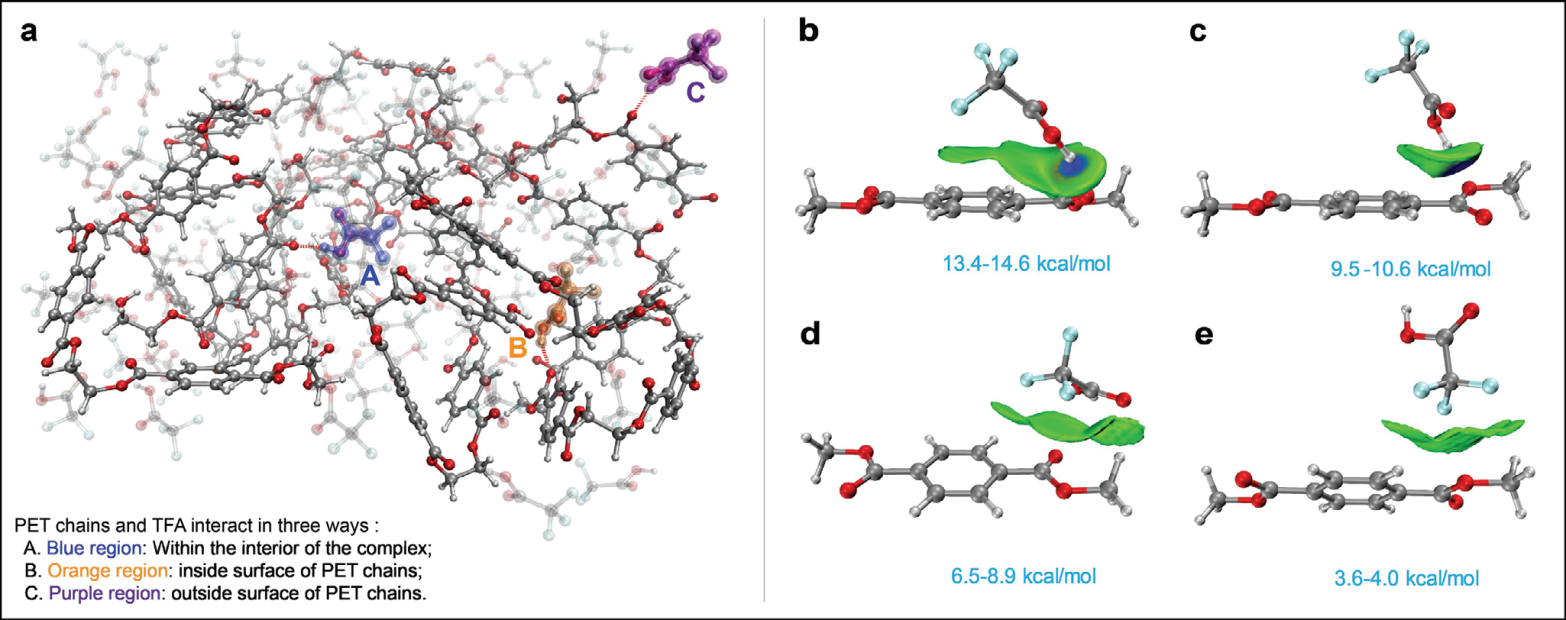
a) Molecular dynamics simulation of PET/TFA (1/2) complex (left); b‐e) visualization of weak interactions in DMT/TFA (1/1) complex (right). All optimized structures were visualized using the VMD 1.9.3 program^[^ ^21^ ^]^ and Multiwfn.^[^ ^22^ ^]^

Molecular dynamics^[^ ^20^ ^]^ simulations observed that TFA undergoes three different chemical environments in the PET/TFA complex (molar ratio = 1:2, PET based on the single repeat unit, **Figure** **3**a), potentially influencing subsequent reactions. 1) Within the interior of the complex (Blue region), TFA disrupted the crystalline region and the regularity of PET chains, enabling porosity and infiltration from nucleophilic reagents. 2) On the inside surface of PET chains (Orange region), the proton of TFA formed hydrogen bonding with the ester carbonyl groups in PET chains, activating the carbonyl of the ester group. 3) On the outside surface of PET chains (Purple region), TFA was readily neutralized by NaOH to form trifluoroacetate, thereby activating nucleophilic reagents (Figure S33, Supporting Information).

To further understand the interaction models between TFA and PET chains, DFT calculations identified four typical interaction modes, comprising two hydrogen bonding models and two van der Waals interactions. Hydrogen bonding forms between the oxygen atom of the ester group and the proton of TFA, with the binding energy ranging from 9.5 to 14.6 kcal mol^−1^ (Figure 3b,c). Van der Waals interactions form between the carboxyl plane of TFA and PET chain, or between the trifluoromethyl group and the ester group, with strengths ranging from 3.6 to 8.9 kcal mol^−1^ (Figure 3d,e). The binding energy of hydrogen bonding formed between PET chains and TFA is stronger than the interactions of PET chains (Figure S26, Supporting Information), which is fundamental to PET's solubility in TFA (Figure S29, Supporting Information).

**Figure 4:**
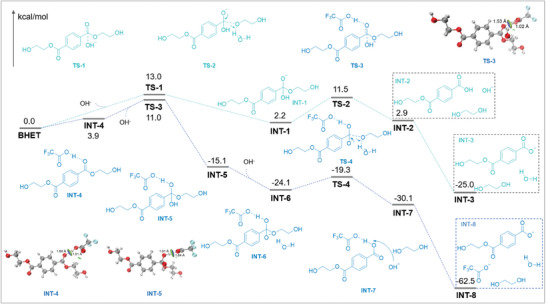
The energy profiles were calculated at the level of M06‐2X‐D3/6‐311+G(d, p)/SMD(water)//B3LYP‐D3(BJ)/6‐31+G(d, p)/PCM(water). All optimized structures were visualized using the VMD 1.9.3 program.

Finally, the polyester monomer bis(2‐hydroxyethyl) terephthalate (BHET) was employed as a theoretical model to investigate the reaction pathways of the ester carbonyl group activated by TFA. Density functional theory (DFT) calculations (**Figure** **4**) revealed that the hydrogen bonding interaction formed between the ester carbonyl groups in BHET and the proton from TFA, which stabilized oxygen anions in the nucleophilic attack process, resulting in the energy barrier of **TS‐3** was 2.0 kcal mol^−1^ lower than that of **TS‐1**, and then the proton of TFA was close to the oxygen atom of ester groups (**INT‐5**), thereby promoting the departure of ethylene glycol, resulting the energy barrier of **TS‐4** was 4.5 kcal mol^−1^ lower than that of **TS‐2**. As the main chain of PET was further cleavaged to the oligomer, which gradually dissolved in aqueous NaOH. TFA was quenched by NaOH to produce sodium trifluoroacetate, completely disrupting the interactions between TFA and the ester carbonyl group. Finally, the oligomer could be efficiently hydrolyzed to monomer at room temperature.

**Figure 5:**
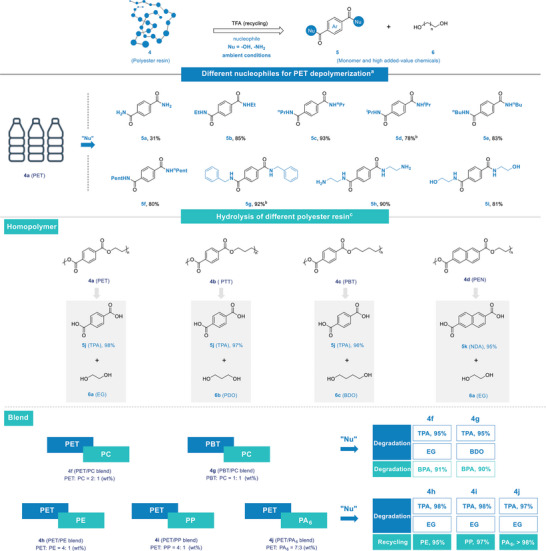
General conditions: a) PET resin (0.25 mmol), TFA (10 equiv.), RNH_2_ (2 mL), r.t., 24 h; b) PET resin (0.25 mmol), TFA (10 equiv.), RNH_2_ (2 mL), TBD (3 equiv.), r.t., 72 h; c) polyesters or blends (1 g), TFA (10 equiv.), 2 N NaOH (aq.), r.t., 8–12 h. EG = ethylene glycol; PDO = propanediol; BDO = butanediol. BPA = bisphenol A.

With the standard procedures and conditions in hand, we attempted depolymerization with various nucleophilic reagents on pre‐activated PET, achieving the upcycling of PET into various high‐value‐added chemicals (**Figure** **5**).^[^ ^23^ ^]^ Pre‐activated PET could be depolymerized directly in NH_3_·H_2_O to generate terephthalamide **5a**. When primary alkyl amines (ethylamine, *n*‐propylamine, *i*‐propylamine, *n*‐butylamine, and *n*‐amylamine) were applied, excellent yields of **5b**, **5c**, **5d**, **5e**, and **5f** were obtained, especially with *n*‐propylamine **5c** achieving a high yield of 93%. *i‐*Propylamine and benzylamine gave lower efficiency possibly due to steric hindrance. The addition of an extra base, triazabicyclo [4.4.0] dec‐5‐ene (TBD), improved their nucleophilicity with increased yields of **5d** and **5g** to 78% and 92%, respectively. It is noteworthy that ethylenediamine and ethanolamine, as nucleophilic reagents, could efficiently depolymerize pre‐activated PET to produce the corresponding amides **5h** and **5i**, in which exposed hydroxyl and amino groups could serve as monomers for polyurethane.^[^ ^24^ ^]^

Polytrimethylene terephthalate (PTT),^[^ ^25^, ^26^ ^]^ polybutylene terephthalate (PBT),^[^ ^27^ ^]^ and polyethylene naphthalate (PEN) are polymerized from different aryl dicarboxylic acids and diols, broadening the application of polyesters,^[^ ^28^ ^]^ along with the challenge for dismantling the compact structure of polyester plastics. Fortunately, efficient hydrolysis of pre‐activated polyesters was realized at room temperature with the recovery rates of the corresponding products exceeding 95%, along with almost quantitative recovery of diols.

Thermoplastic polyesters are often blended with other plastics for altering their inherent properties, such as polycarbonate (PC) blended with polyester enhancing the toughness, rigidity, and heat resistance of polyester. PET/PC blend **4f** and PBT/PC blend **4g**,^[^ ^29^, ^30^ ^]^ after pre‐activation with TFA, efficiently degrade at room temperature into TPA (95% yield), corresponding diols (quant.), bisphenol A (>90% yield, Figure S20, Supporting Information). PET/polyethylene (PE) blend **4h**, modified with hydrophobic PE for enhanced toughness, improves the impact strength and crystallization rate of PET.^[^ ^31^ ^]^ Blend **4i**, composed of PET and polypropylene (PP), reduces PET's sensitivity to water,^[^ ^32^ ^]^ allowing for the easy separation of resistant PE and PP through simple filtration during the hydrolysis process. This facilitates the directed efficient depolymerization of PET in PET/PE and PET/PP blends, with separate recovery of PE and PP (Figures S21–S22, Supporting Information). Blend **4j**, formed by blending PET and polyamide 6 (PA_6_), enhanced PET's crystallization performance, permeation resistance, and mechanical properties.^[^ ^33^ ^]^ Depolymerization of PET in PET/PA_6_ blend was achieved in the hydrolysis process and separated with the recovery of PA_6_ (Figure S19, Supporting Information).

**Figure 6:**
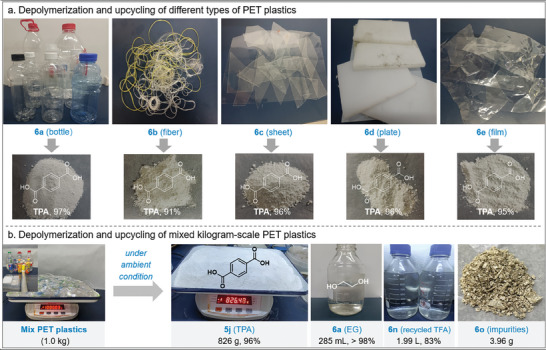
Real PET plastic waste depolymerization. a) PET fiber, bottle, sheet, plate, film (1.0 g), TFA (10 equiv.); b) mixed kilogram‐scale PET plastics (1.0 kg), TFA (6 equiv., 2.4 L).

Polyester resin, enhanced with a variety of additives including plasticizers, adhesives, crosslinking agents, etc is utilized to optimize the properties of polyester plastics. Therefore, the complexity introduces challenges to the depolymerization process. Moreover, plastic waste, characterized by water stains, blemishes, and impurities (labels, debris, etc.), presents obstacles to the depolymerization process as well. To further explore the potential practical applications, we undertook the task of degrading and recovering high‐value‐added products from diverse real PET waste plastics (**Figure** **6**). PET plastics can be categorized into bottle (**6a**), fiber (**6b**), sheet (**6c**), plate (**6d**), and film (**6e**) based on their product types. Under standard conditions, PET fiber demonstrated an excellent recovery yield of 91% for TPA, while other types of PET products achieved recovery yields surpassing 95%.

A kilogram of diverse types of PET was collected, including disposable beverage bottles (320 g), fibers (50 g), sheets (150 g), plates (160 g), films (170 g), and resins (150 g). Mixed depolymerization resulted in a 96% yield for TPA, 98% for ethylene glycol (EG), the recovery of TFA (1.99 L), and the isolation of impurities (3.96 g) under standard conditions. The mixed PET plastics were fully dissolved with 6 equivalents of TFA, and then excess TFA could be recovered through rotary evaporation with an 83% recovery rate, only 1 equivalent of TFA was required. The resulting complex was completely depolymerization to sodium terephthalate, which dissolved in NaOH (aq.). Then, impurities (labels and debris) were separated through filtration. Further pH adjustments precipitated monomers (TPA) from the filtration, achieving 96% TPA recovery, meanwhile EG was recovered. This approach not only showcases the efficient depolymerization of kilogram‐scale mixed real PET waste but also highlights the potential for recovering individual monomers.

## 3 Conclusion

In conclusion, the activation by TFA facilitated the closed‐loop recycling and the diversified upcycling of PET effectively under ambient conditions with excellent recovery yields. Moreover, this strategy extends to the recycling of other polyester plastics and blended polyester plastics, with excellent recycling efficiency surpassing 95%. Through DSC studies, SEM analysis, and XRD curves, the topological and morphological characteristics from the PET activation process were characterized, while GPC curves and NMR studies were employed to monitor the variation occurring in molecular structures during the depolymerization process. DFT calculation and molecular dynamics simulations demonstrated that the synergistic effect of polyfluorine and hydrogen bonding promoted the decrystallization of semi‐crystalline polyesters and the pre‐activation of the ester groups process. Finally, kilogram‐scale depolymerization of mixed real PET waste addressed the potential for the sustainable recovery of polyester plastics.

## Conflict of Interest

The authors declare no conflict of interest.

## Supporting information

Supporting Information

## Data Availability

The data that support the findings of this study are openly available in [No] at [DOI], reference number 1.
