# Supplementary material for: From Polyester Plastics to Diverse Monomers via Low‐Energy Upcycling
Source: Adv Sci (Weinh). 2024 Apr 16;11(25):2403002. doi: 10.1002/advs.202403002 (PMC11220695; doi:10.1002/advs.202403002)

ADVANCED SCIENCE
Open Access

Supporting Information

for *Adv. Sci.*, DOI 10.1002/advs.202403002

From Polyester Plastics to Diverse Monomers via Low-Energy Upcycling

*Lei Ji, Jiaolong Meng, Chengliang Li, Ming Wang and Xuefeng Jiang**

Supporting Information

From Polyester Plastics to Diverse Monomers via Low-Energy Upcycling

Lei Ji,+[a] Jiaolong Meng,+[a] Chengliang Li,[a] Ming Wang,[a] and Xuefeng Jiang*[a],[b]

[a] L. Ji, J. Meng, C. Li, M. Wang, and Prof. X. Jiang
State Key Laboratory of Molecular & Process Engineering, School of Chemistry and Molecular Engineering
East China Normal University
North Zhongshan Road 3663, Shanghai 200062, China
E-mail: xfjiang@chem.ecnu.edu.cn.

[b] Prof. X. Jiang
School of Chemistry and Chemical Engineering
Henan Normal University
Xinxiang, Henan 453007, China

[+] These authors contributed equally.

Table of Contents

I. General Information.......... S2

II. Optimization for the hydrolysis of PET.......... S4

III. Procedures for the depolymerization of various polyesters.......... S10

IV. Characterization of PET depolymerization.......... S19

V. Mechanistic studies.......... S30

VI. Characterization of polyester depolymerization products.......... S110

VII. References.......... S117

VIII. NMR spectra.......... S119

I. General Information.

All polyester resins are commercially available unless otherwise noted. including polyethylene terephthalate (PET) resin particles (white granular, viscosity, 0.8-0.83 mPa·s, purchased by *Bidepharm*, China, http: www.bidepharmatech.com) was used as a model for the optimization of the hydrolysis/ammonolysis conditions of PET; PET resin particle (*Bidepharm*) as the model was used for the ammonolysis by different amines; PET resin powder (purchased by *Dongguan Ruixiang plastic Co., LTD*, China) was used as model for the mechanism studies (GPC, SEM, EDS, XRD, DSC, TGA, FT-IR, NMR); PTT (purchased by *Klamar*, China, http: www.klamar-reagent.com), PBT (*Klamar*, China), PEN (purchased by *Dongguan Dongwo plastic raw materials Co., LTD*, China, https:www.pvc123.com/b-DONGWOSJXLI/introduce) virgin resin particle was used as model for the hydrolysis of different polyesters." PE (polyethylene) and PP (polypropylene), PA_6 (polyamide 6) (purchased by *Macklin*, China, https://www.macklin.cn/); PET/PC (polycarbonate) blend, PBT/PC blend (purchased by *Dongguan Dongwo plastic raw materials Co., LTD*, China).

Figure S1. The morphology of different polyester resins.

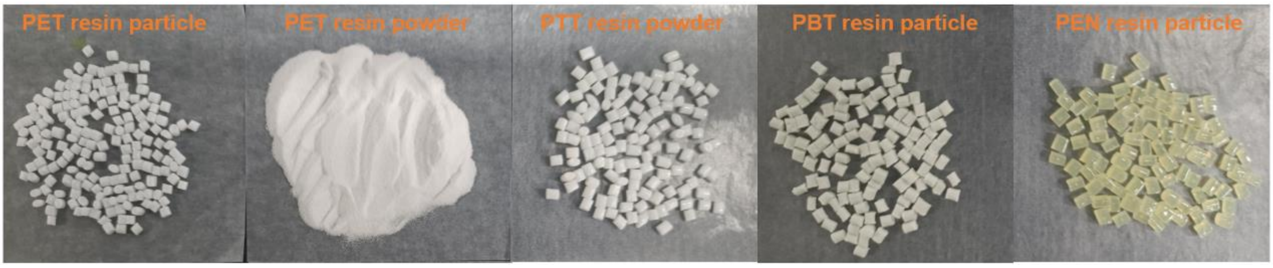

All reaction reagents including trifluoroacetic acid (TFA), trifluoromethanesulfonic acid (CF_3SO_3H), perfluoropropionic acid (CF_3CF_2COOH), 1,5,7-triazabicyclo [4.4.0] dec-5-ene (TBD), NaOH, HCl, NH_4OH, ethylamine, *n*-propylamine, *i*-propylamine, *n*-butylamine, *n*-amylamine, benzylamine, ethylenediamine, monoethanolamine are commercially available unless otherwise noted. ^1H, ^{13}C, and ^{19}F NMR spectra were recorded on 400 MHz or 500 MHz NMR spectrometers (Bruker AVANCE) using DMSO-d_6 or D_2O. ^1H NMR and ^{19}F NMR tracking spectra were recorded on 400 MHz or 500 MHz NMR spectrometers (Bruker AVANCE) using $CDCl_3$. Chemical shifts are reported in parts per million (ppm). Chemical

shifts for protons are reported in parts per million relatives to $CDCl_3$ (δ 7.26). Chemical shifts for carbon are reported in parts per million relatives to $CDCl_3$ (δ 77.0). Chemical shifts for protons are reported in parts per million relatives to D_2O (δ 4.79). Chemical shifts for protons are reported in parts per million relatives to DMSO-d_6 (δ 2.50). Chemical shifts for carbon are reported in parts per million relatives to DMSO-d_6 (δ 39.52). Data are represented as follows: chemical shift, multiplicity (br = broad, s = singlet, d = doublet, t = triplet, q = quartet, m = multiplet), coupling constants in Hertz (Hz) integration.

Relative molecular weights and molecular weight distributions were measured by the conventional gel permeation chromatography (**GPC**) system (Agilent 1260 Infinity II) equipped with a refractive index (RI) detector; GPC measurements were carried out at 30°C using HFIP as eluent with a flow rate of 1.0 mL/min. The system was calibrated with linear polystyrene standards; The micro-morphology of the samples was observed by using the scanning electron microscope (**SEM**) on Zeiss Gemini SEM450, and the samples were sprayed with gold before the test; The surface ingredient of the samples was observed by using Energy Dispersive Spectrometer (**EDS**) on the Zeiss Gemini SEM450; The decomposition temperature of the samples was observed by using thermogravimetric analysis (**TGA**) on STA449F3; The Glass Transition Temperature (*Tg*) was performed by differential scanning calorimetry (**DSC**) with the device (Shearwater Q2000); An Ultima IV X-ray powder diffractometer equipped with Cu-Kα radiation (λ = 1.5406 Å) was used to record crystal phase X-ray diffraction (**XRD**) patterns; **IR spectra** were recorded on TENSOR (27) Series FT-IR 241 Spectrometers; The Molecular weight was measured by Inductively Coupled Plasma Mass Spectrometry (**ICP-MS**) on NexION 2000-(A-10). Column chromatography was performed with silica gel (300 - 400 mesh ASTM).

II. Optimization for the hydrolysis of PET.

Figure S2. Screening of activating reagents.

a) Lewis acids and Brønsted acids**.**

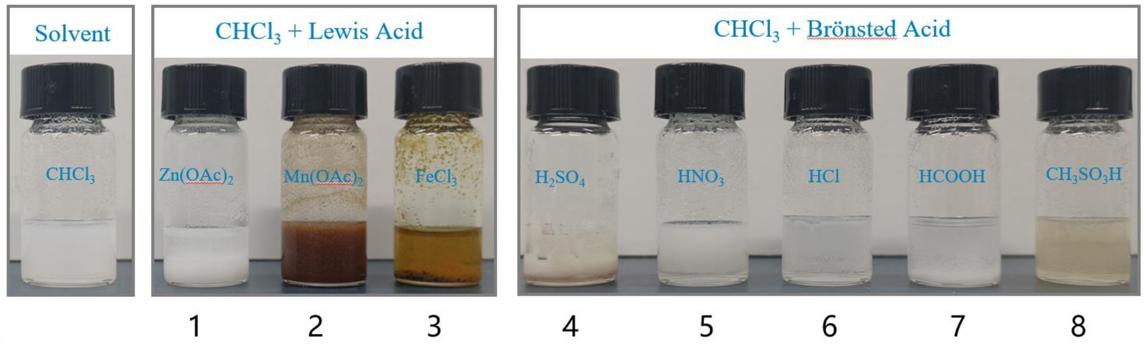

b) Acetic acid is substituted by different halogens.

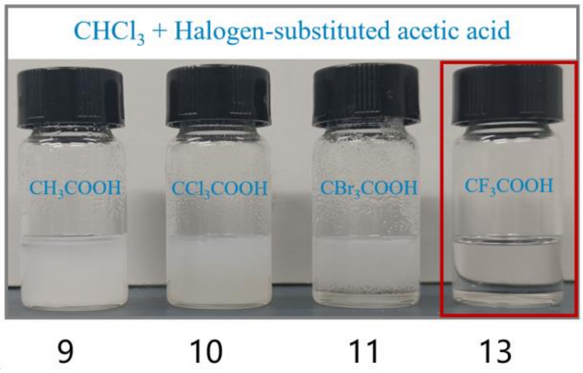

c) Brønsted acids containing fluorine atoms.

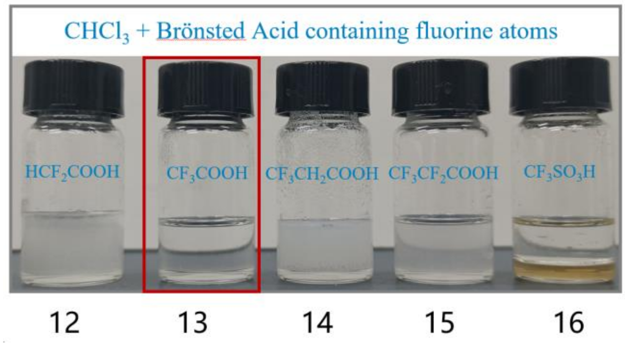

d) Salts containing fluorine atoms.

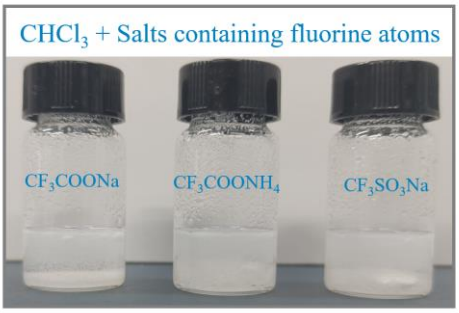

17 18 19

Table S1. One-pot strategy.

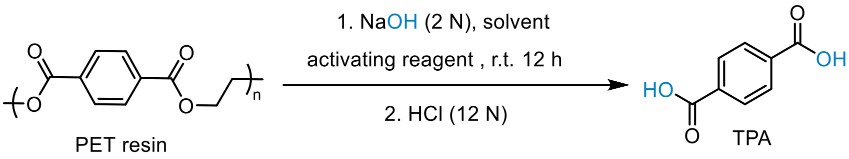

entry	solvent	activating reagent	yield
1	$CHCl_3$	HCF_2COOH	<10
2	$CHCl_3$	CF_3COOH	21
3	$CHCl_3$	CF_3CH_2COOH	<10
4	$CHCl_3$	CF_3CF_2COOH	22
5	CH_2Cl_2	CF_3SO_3H	27
6	DCE	CF_3COOH	trace
7	MeOH	CF_3COOH	trace
8	CH_3CN	CF_3COOH	trace
9	H_2O	CF_3COOH	trace

General condition: PET resin particles (48 mg, 0.25 mmol), solvent (2 mL), activating reagent (5 equiv.), and 2 N NaOH (aq.) were added after PET completely dissolved in $CHCl_3$ containing activating reagent.

Table S2. One-pot stepwise strategy.[a]

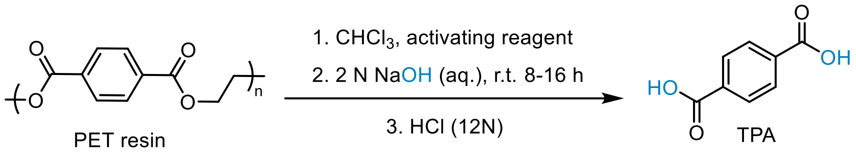

entry	solvent	activating reagent	yield
1	$CHCl_3$	$Zn(OAc)_2$[b]	7
2	$CHCl_3$	$Mn(OAc)_2$[b]	8
3	$CHCl_3$	$FeCl_3$[b]	6
4	$CHCl_3$	H_2SO_4	13
5	$CHCl_3$	HNO_3	8
6	$CHCl_3$	HCl	5
7	$CHCl_3$	HCOOH	6
8	$CHCl_3$	CH_3SO_3H	9
9	$CHCl_3$	CH_3COOH	7
10	$CHCl_3$	CCl_3COOH	9
11	$CHCl_3$	CBr_3COOH	10
12	$CHCl_3$	HCF_2COOH	36
13	$CHCl_3$	CF_3COOH	83
14	$CHCl_3$	CF_3CH_2COOH	12
15	$CHCl_3$	CF_3CF_2COOH	85
16	$CHCl_3$	CF_3SO_3H	91
17	$CHCl_3$	CF_3COONa	4
18	$CHCl_3$	CF_3COONH_4	7
19	$CHCl_3$	CF_3SO_3Na	5

General conditions: a) PET resin particles (48 mg, 0.25 mmol), solvent (2 mL), activating reagent (5 equiv.), After PET was completely dissolved in $CHCl_3$ containing the activating reagent, $CHCl_3$ was removed and then 2 N NaOH (aq.) was added; b) activating reagent (3 equiv.).

Table S3. Price of perfluorinated substituted organic acids.

Entry	perfluorinated organic acid	price ($)
1	CF_3COOH	170
2	CF_3CF_2COOH	4501
3	CF_3SO_3H	1382

Notes: The current exchange rate: 1 US dollar = 7.2 CNY. Provided by Sigma-Aldrich (500 g, purity: 99%). https://www.sigmaaldrich.cn/.

Table S4. Optimization of different volumes of $CHCl_3$ and TFA.

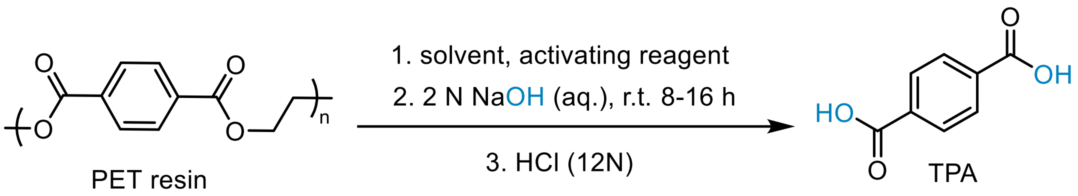

entry	solvent/$CHCl_3$	activating reagent/TFA	yield
1	2.0 mL	0 mL	4
2	2.0 mL	0.06 mL/ 3 eq.	45
3	2.0 mL	0.08 mL/ 4 eq.	61
4	2.0 mL	0.10 mL/ 5 eq.	83
5	1.0 mL	0.10 mL/ 5 eq.	84
6	0.5 mL	0.10 mL/ 5 eq.	85
7	0.3 mL	0.10 mL/ 5 eq.	87
8	0.1 mL	0.10 mL/ 5 eq.	88
9	0.05 mL	0.10 mL/ 5 eq.	90
10	/	0.10 mL/ 5 eq.	91
11	/	0.20 mL/ 10 eq.	98
12	/	0.30 mL/ 15 eq.	98
13	/	0.40 mL/ 20 eq.	97

General condition: PET resin particles (48 mg, 0.25 mmol); After PET was completely dissolved in a solvent, the solvent was removed and then 2 N NaOH (aq.) was added.

Table S5. Optimization of the ammonolysis of PET.[a]

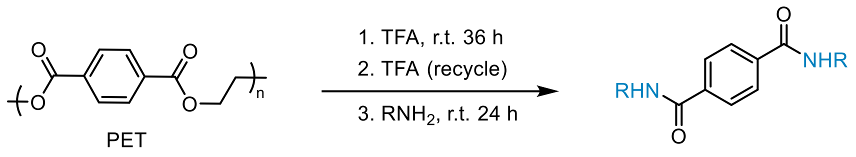

entry	PET source	activating reagent	RNH_2	yield
1	resin	TFA(10 eq.)	NH_3•H_2O	15
2	resin	TFA(10 eq.)	$EtNH_2$	75
3	resin	TFA(10 eq.)	$^nPrNH_2$	93
4	resin	TFA(10 eq.)	$^nBuNH_2$	80
5[b]	resin	TFA(10 eq.)	$^nPrNH_2$	13
6[c]	resin	TFA(10 eq.)	$^nPrNH_2$	82
7[d]	resin	TFA(10 eq.)	$^nPrNH_2$	67
8	resin	TFA(5 eq.)	$^nPrNH_2$	88
9	resin	TFA(20 eq.)	$^nPrNH_2$	84
10	fiber	TFA(10 eq.)	$^nPrNH_2$	85
11	bottle	TFA(10 eq.)	$^nPrNH_2$	95
12	plate	TFA(10 eq.)	$^nPrNH_2$	94
13	film	TFA(10 eq.)	$^nPrNH_2$	92

a) General conditions: PET resin particles (48 mg, 0.25 mmol), RNH_2 (2 mL), after PET was completely dissolved in TFA, TFA was removed and then RNH_2 was added; b) 1 mL $^nPrNH_2$ dissolved in 1 mL MeOH instead of $^nPrNH_2$ (2 mL); c) $^nPrNH_2$ (1 mL); d) $^nPrNH_2$ (0.5 mL).

III. Procedures for the depolymerization of various polyesters

1. General procedure for the hydrolysis of PET.

General procedure A: PET resin (0.25 mmol, 48 mg) was added into 10 mL dried Schlenk tube, then TFA (2.5 mmol, 10 equiv.) was added to dissolve PET. After PET completely dissolved in TFA (It takes 36 h for PET resin particles to dissolve, and 6 h for PET resin powder to dissolve), excess TFA was removed by rotary evaporator under vacuum below 45°C, unremoved TFA formed TFA-treated PET (white solid) with PET, followed NaOH (2 N) (5.0 equiv.) was added to depolymerize TFA-treated PET, completely depolymerization of TFA-treated PET to sodium terephthalate and EG dissolved in NaOH (aq.), it takes approximately 6 h to form yellowish clarified solution, terephthalic acid (TPA) was precipitated from the clarified solution when used HCl (conc.) adjust pH = 3. Finally, TPA was separated and purified by vacuum filtration and dried in an 80°C oven (white solid).

2. General procedures for the ammonolysis of PET.

General procedure B**:** PET resin particles (0.25 mmol, 48 mg) were added into 10 mL dried Schlenk tube, then TFA (2.5 mmol, 10 equiv.) was added to dissolve PET. After PET completely dissolved in TFA*,* excess TFA was removed by a rotary evaporator under a vacuum below 45°C, followed by 2.0 mL RNH_2 (ethylamine, *n*-propylamine, *n*-butylamine, *n*-amylamine) was added to depolymerize TFA-treated PET, obtaining white suspension, followed by quenching with silica gel (note: the analysis of TLC before quenching). After evaporation of the solvent under vacuum, the residue was purified by column chromatography to yield the corresponding products (yield of **5b** 85%, **5c** 93%, **5e** 83%, **5f** 80%, white solid).

General procedure C**:** Other PET types (0.25 mmol, 48 mg) instead of PET resin particles in general procedure B (It takes 10 min for PET fiber to dissolve, 10 min for PET bottle to dissolve, 1 h for PET plate to dissolve, 20 min for PET sheet to dissolve, 15 min for PET film to dissolve).

Separation and purification of **5a**: TFA-treated PET was obtained by general procedure B,

then NH_4OH (2 mL) was added to depolymerize the TFA-treated PET, depolymerization of TFA-treated PET takes approximately 72 h to obtain a white suspension. This suspension was filtered under vacuum removed excess NH_4OH, and the residue was dissolved in MeOH. Subsequently, it was quenched with silica gel (note: the analysis of TLC was conducted before quenching). After evaporation of MeOH under vacuum, the residue was purified by column chromatography to yield the corresponding **5a** (yield 31%, white solid).

Separation and purification of **5d** and **5g**: TFA-treated PET was obtained by general procedure B, then TBD (104 mg, 0.75 mmol, 3 equiv.) and *i*-propylamine (2 mL) or benzylamine (2 mL) were added to depolymerize the TFA-treated PET, completely depolymerization of TFA-treated PET takes approximately 72 h to obtain clarified solution, followed petroleum ether was added and then filtered under vacuum removed excess *i*-propylamine or benzylamine obtained the residue, the residue was purified by recrystallization to yield the corresponding products (yield of **5d** 78%, recrystallization solvent, DCM/PE, white solid; yield of **5g** 92%, recrystallization solvent, DCM/PE, white solid).

Separation and purification of **5h:** TFA-treated PET was obtained by general procedure B, then ethylenediamine (2 mL) was added to depolymerize TFA-treated PET, completely depolymerization of TFA-treated PET to dissolve in ethylenediamine, followed excess ethylenediamine was removed by vacuum distillation to obtain the residue, the residue was washed by MeOH/DCM (10 mL, v/v = 2:3) and purified by recrystallization (H_2O/CH_3CN) to yield the corresponding product **5h** (90%, white solid).

Separation and purification of **5i**: TFA-treated PET was obtained by general procedure B, then ethanolamine (2 mL) was added to depolymerize TFA-treated PET, completely depolymerization of TFA-treated PET to dissolve in ethanolamine, followed DCM was added into the reaction solution, then the residue was precipitated from the reaction solution and purified by recrystallization to yield the corresponding **5i** (yield 81%, recrystallization solvent, MeOH/DCM, white solid).

3. General procedures for the hydrolysis of various polyesters.

General procedure D for the hydrolysis of PET/PTT/PBT: Polyester resin particles (1.0 g) was added into 25 mL dried round-bottom flask, then TFA (10 equiv.) was added into the flask to dissolve polyester. After the polyester completely dissolved in TFA (It takes 3 h for PET to dissolve, 4 h for PTT resin to dissolve, 6 h for PBT resin to dissolve), excess TFA was removed by rotary evaporator under vacuum below 45^oC, unremoved TFA formed TFA-treated polyester (white solid) with polyester, followed 2 N NaOH (aq.) was added to depolymerize TFA-treated polyester, completely depolymerization of TFA-treated polyester to sodium terephthalate dissolved in NaOH (aq.), it takes approximately 8-14 h to form yellowish clarified solution, TPA was precipitated from the clarified solution when used HCl (12 N) adjust pH = 3. Finally, TPA was separated by vacuum filtration and then dried in an 80^oC oven (PET, yield 98%; PTT, yield 97%; PBT, yield 96%, white solid).

General Procedure E for the separation and purification of diols**:** After TPA was precipitated from the clarified solution when HCl (12 N) was used, adjusting pH = 3. Filtered under vacuum separated TPA to obtain white clarified filtration solution, then water and TFA were separated by vacuum distillation with a rotary evaporator to obtain diols (**6a**, glycol; **6b**, propanediol; **6c**, butanediol) and NaCl (s), filtered under vacuum and then washed NaCl (s) with DCM/EtOH (v/v = 1:1) to obtain mixture filtration solution of diols and DCM/EtOH, DCM/ethanol was removed by vacuum distillation to yield crude diols (Contains a small amount of TFA), finally, diols were separated and purified by vacuum distillation.

Procedure F for the hydrolysis of PEN: PEN resin (4.1 mmol, 1.0 g) was added into 25 mL dried round-bottom flask, then TFA (41 mmol, 10 equiv.) was added into the flask to dissolve PEN. After 8 h of reaction (white suspension), excess TFA was removed by rotary evaporator under vacuum below 45^oC, followed by NaOH (2 N) (20.0 mmol, 4.9 equiv.) was added to depolymerize the TFA-treated PEN, completely depolymerization of the TFA-treated PEN to sodium naphthalene-2,6-dicarboxylate dissolved in NaOH (aq.), it takes

approximately 12 h to form clarified solution, sodium naphthalene-2,6-dicarboxylate was precipitated from the clarified solution when used HCl (12 N) adjust pH = 3. Finally, naphthalene-2,6-dicarboxylic acid (NDA) was separated by vacuum filtration and dried in an 80°C oven (yield 93%, white solid), then EG was separated and purified by general procedure E.

Figure S3 The hydrolysis flow chart of polyesters.

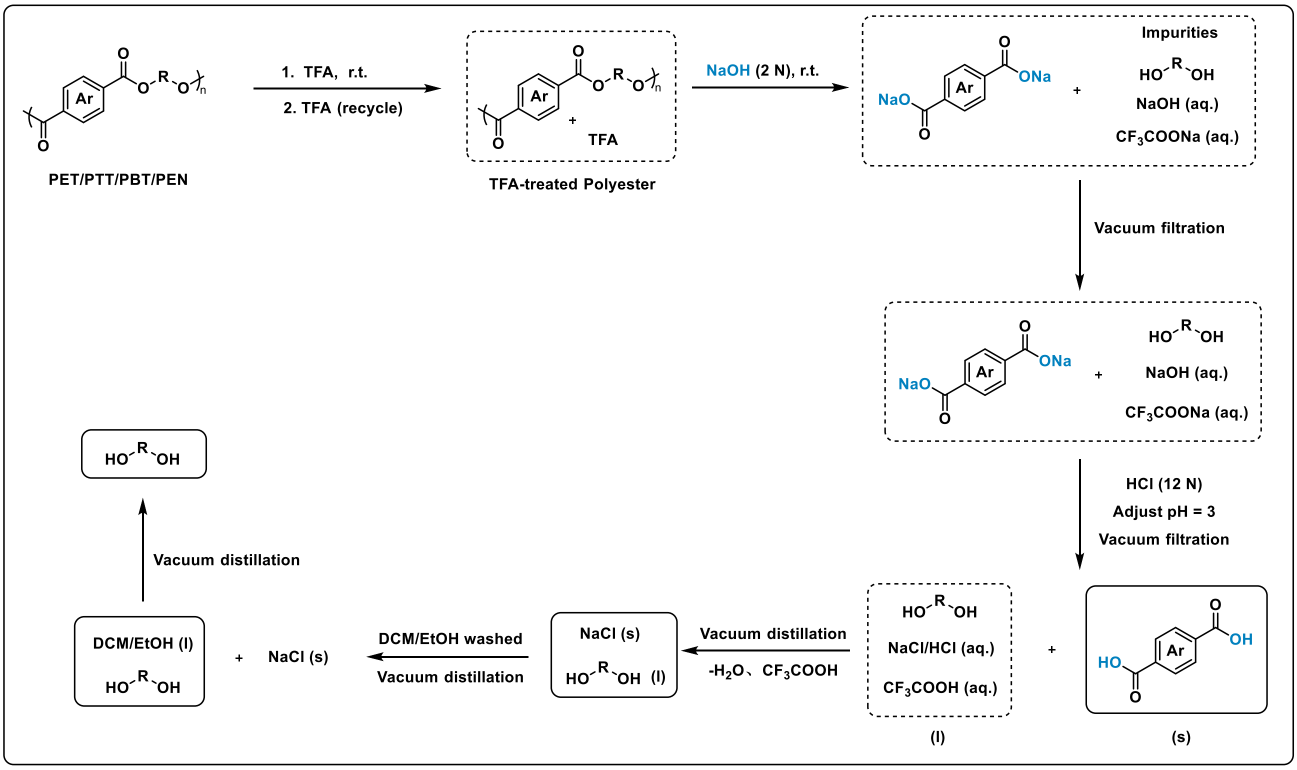

4. The hydrolysis of various polyester/plastic blends.

General Procedure G for the hydrolysis of PET/PC blend and PBT/PC blend**:** PET/PC blend ($m_{(PET)}$:$m_{(PC)}$ = 2:1) or PBT/PC blend ($m_{(PBT)}$:$m_{(PC)}$ = 1:1) (1.0 g) was added into 25 mL dried round-bottom flask, then TFA (10 equiv.) was added into flask to dissolve PET/PC blend or PBT/PC blend. After the PET/PC blend or PBT/PC blend completely dissolved in TFA (notes: both PET and PBT were dissolved in TFA, the solubility of PC plastics in TFA was poor, white suspension), excess TFA was removed by rotary evaporator under vacuum below 45°C, followed NaOH (2 N) was added, completely depolymerization of PET or PBT and PC to sodium terephthalate and sodium 4,4'-(propane-2,2-diyl)diphenolate dissolved in NaOH (aq.), it takes approximately 12 h, TPA and bisphenol A were precipitated from the clarified filtrate when used HCl (12 N) adjust pH = 3, followed TPA and bisphenol A were separated by vacuum filtration, bisphenol A was separated and purified by DCM/EtOH (v/v = 1:1) washed, TPA and BPA dried in 80°C oven, then EG and butanediol was separated and purified by general procedure E.

Procedure H for the hydrolysis of PET/PE blend and PET/PP blend**:** PET/PE blend ($m_{(PET)}$:$m_{(PE)}$ = 4:1) or PET/PP blend ($m_{(PET)}$:$m_{(PP)}$ = 4:1) (1.0 g) was added into 25 mL dried round-bottom flask, then TFA (10 equiv.) was added into the flask to dissolve PET/PE blend or PET/PP blend. It takes 6 h for PET to dissolve (notes: both PE and PP were insoluble in TFA, white suspension), excess TFA was removed by rotary evaporator under vacuum below 45°C, followed 2 N NaOH (aq.) was added to depolymerize PET, completely depolymerization of PET to sodium terephthalate dissolved in NaOH (aq.), it takes approximately 12 h (white suspension), unreacted PE or PP was separated by vacuum filtration (filtration solid), then TPA was precipitated from the clarified filtrate when used HCl (12 N) adjust pH = 3. Finally, TPA was separated by vacuum filtration and dried in 80°C oven, then EG was separated and purified by general procedure E.

Procedures I for the hydrolysis of PET/PA_6 (resin powder) blend: PET/PA_6 blend ($m_{(PET)}$:$m_{(PA6)}$ = 7:3) (1.0 g) was added into 25 mL dried round-bottom flask, then TFA (10

equiv.) was added into flask to dissolve PET/PA_6 blend. After the PET/PA_6 blend completely dissolved in TFA (It takes 16 h for PET/PA_6 blend to dissolve, notes: both PET and PA_6 were dissolved in TFA), excess TFA was removed by rotary evaporator under a vacuum below 45°C, followed 2 N NaOH (aq.)was added to depolymerize PET, completely depolymerization of PET to sodium terephthalate dissolved in NaOH (aq.), it takes approximately 12 h (white suspension), unreacted PA_6 was separated by vacuum filtration (filtration solid), then TPA was precipitated from the clarified filtrate when used HCl (12 N) adjust pH = 3. Finally, TPA was separated by vacuum filtration and dried in 80°C oven, then EG was separated and purified by general procedure E.

5. The hydrolysis of various types of PET.

Procedures J: PET (5.2 mmol, 1.0 g) was added into a 25 mL dried round-bottom flask, then TFA (52 mmol, 10 equiv.) was added into flask to dissolve PET. After PET completely dissolved in TFA, excess TFA was removed by rotary evaporator under vacuum below 45°C, followed by 2 N NaOH (aq.) (20.8 mmol, 4.0 equiv.) was added to depolymerized TFA-treated PET, completely depolymerization of TFA-treated PET to sodium terephthalate dissolved in NaOH (aq.), it takes approximately 4 - 8 h to form yellowish clarified solution, TPA was precipitated from the clarified solution when used HCl (12 N) adjust pH = 3. Finally, TPA was separated by vacuum filtration and dried in an 80°C oven (white solid), TFA and H_2O in the filtrate were removed by vacuum distillation to obtain EG(l) and NaCl(s), then washed by DCM/EtOH (v/v = 1:1) to separate EG.

Figure S4. The hydrolysis flow chart of various types PET.

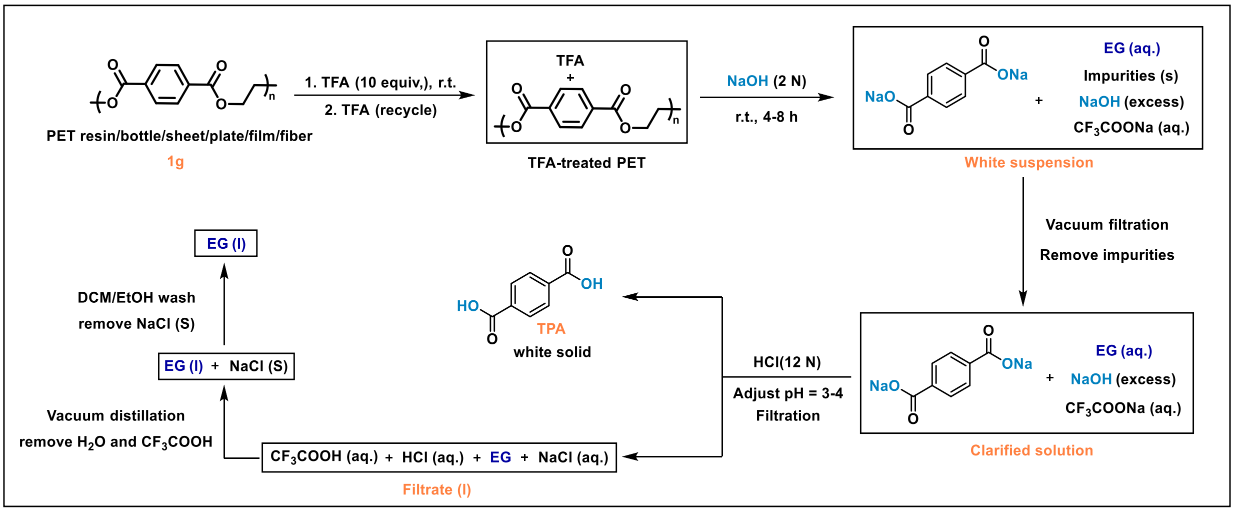

6. Procedures for the hydrolysis of kilogram PET.

Procedure of TPA recycling**:** 1 kg PET including PET resin particles (100 g white granular, viscosity: 0.8-0.83 mPa·s), PET resin powder (50 g), PET single-use beverage bottles (320 g), PET fiber (50 g), PET plate (160 g), PET sheet (150 g), PET film (170 g) were cut into small pieces, then PET small pieces (1 kg, 5.2 mol) were added into 5 L oven-dried three-neck flask. TFA (31.3 mol, 2.4 L, 6 equiv.) was added into the flask to dissolve PET, stirred by mechanical stirring, after 1 kg PET completely dissolved in TFA, excess TFA was recycled by rotary evaporator under vacuum below 45°C (recycled TFA, 1.99 L, recycled yield 83%), followed 2 N NaOH (aq.) was added to depolymerize TFA-treated PET, completely depolymerization of TFA-treated PET to sodium terephthalate dissolved in NaOH (aq.), it takes approximately 6 h to form white solid suspension (sodium terephthalate saturated in the solution), filtered under vacuum (1 L sand core funnel) and then washed the filtration solid with water to obtain unreacted impurities (such as other plastics, tag, adhesives and debris-3.96 g) and yellowish clarified filtration solution. TPA was precipitated from the clarified solution when HCl (12 N) was used, adjusting pH = 3. Finally, TPA was separated by vacuum filtration and dried in an 80°C oven (white solid, 826 g, recovery yield 96%).

Procedure of EG recycling: After TPA was precipitated from the clarified solution when used

HCl (12 N) adjust pH = 3. filtered under vacuum separated TPA to obtain light white clarified filtration solution, then water and TFA were separated by vacuum distillation with rotary evaporator to obtain EG and NaCl(s), filtered under vacuum and then washed NaCl(s) with DCM/EtOH (v/v = 1:1) to obtain mixture filtration solution of EG and DCM/EtOH, DCM/EtOH was removed by vacuum distillation to obtain crude EG (Contains a small amount of TFA), finally, crude EG was purified by vacuum distillation.

Figure S5. The hydrolysis flow chart of kilogram scale PET.

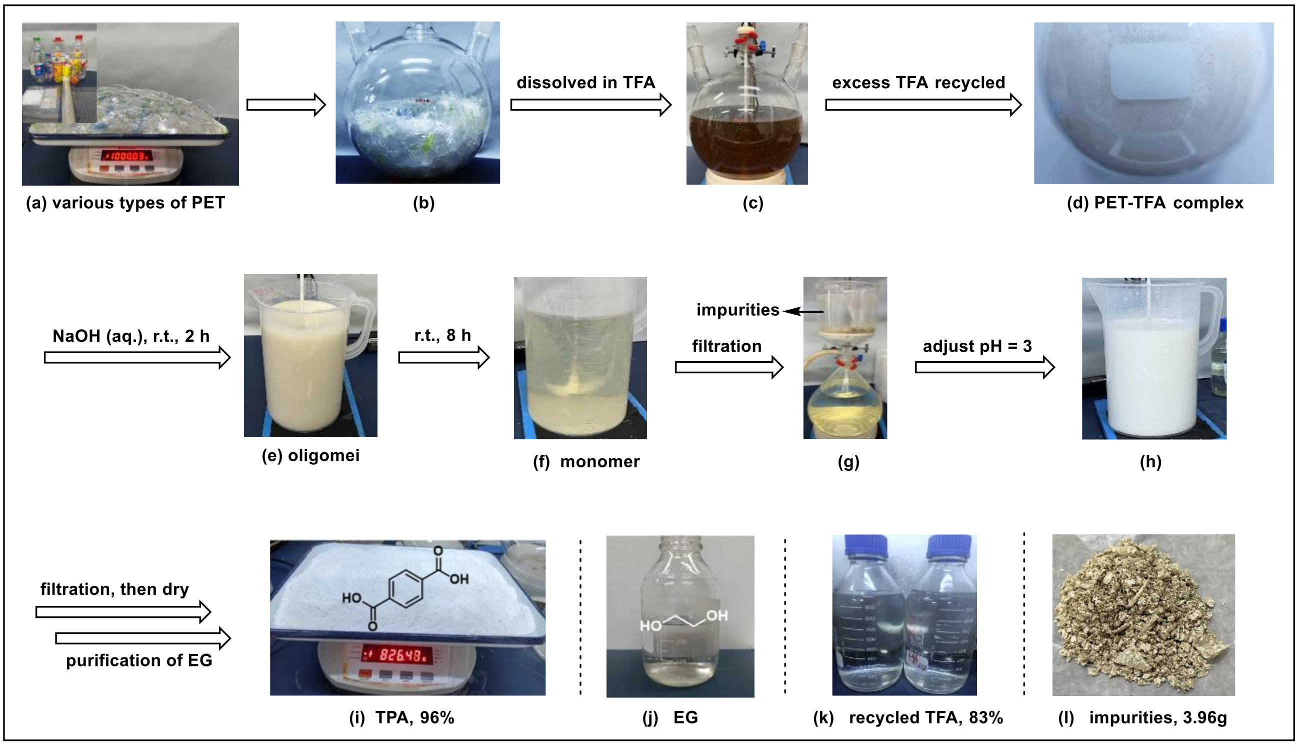

7. Procedures for excess TFA recycling.

Procedure of TFA recycling: In 5 L three-neck flask, after 1 kg PET completely dissolved in TFA (2.4 L), then excess TFA was recycled by rotary evaporator under vacuum below 45°C, 2 L TFA was recycled, recycled yield of TFA 83 %.

Recycled TFA was applied to PET hydrolysis: 100 g PET sheet (0.52 mol) was completely dissolved in 400 mL once-recycled TFA (5.2 mol, 10 equiv.), undergo once hydrolysis process obtained 323 mL twice-recycled TFA and 83.8 g TPA (recycled yield of TPA, 97 %

and recycled yield of TFA, 81 %), then 100 g PET sheet (0.52 mol) was completely dissolved in 323 mL twice-recycled TFA (4.2 mol, 8.1 equiv.), Undergo twice hydrolysis process obtained 246 mL third-recycled TFA and 80.5 g TPA (recycled yield of TPA, 93 % and recycled yield of TFA, 76 %), finally, 100g PET sheet (0.52 mol) was completely dissolved in 246 mL third-recycled TFA (3.2 mol, 6.2 equiv.), Undergo third hydrolysis process obtained 168 mL fourth-recycled TFA and 78.7 g TPA (recycled yield of TPA, 91 % and recycled yield of TFA, 68 %). TFA was recycled for three consecutive times can still be used for efficient hydrolysis of PET at room temperature.

Figure S6. Recycled-TFA was applied to PET hydrolysis.

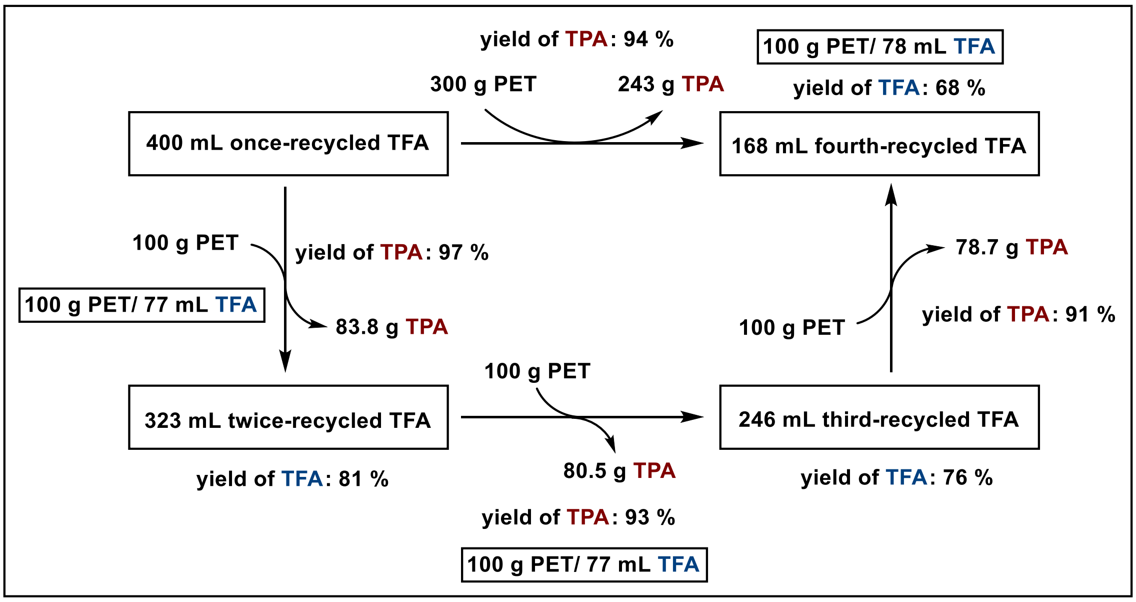

IV. Characterization of PET depolymerization.

1. ^{1}H NMR and ^{19}F NMR studies.

Figure S7. NMR spectra of TFA-PET

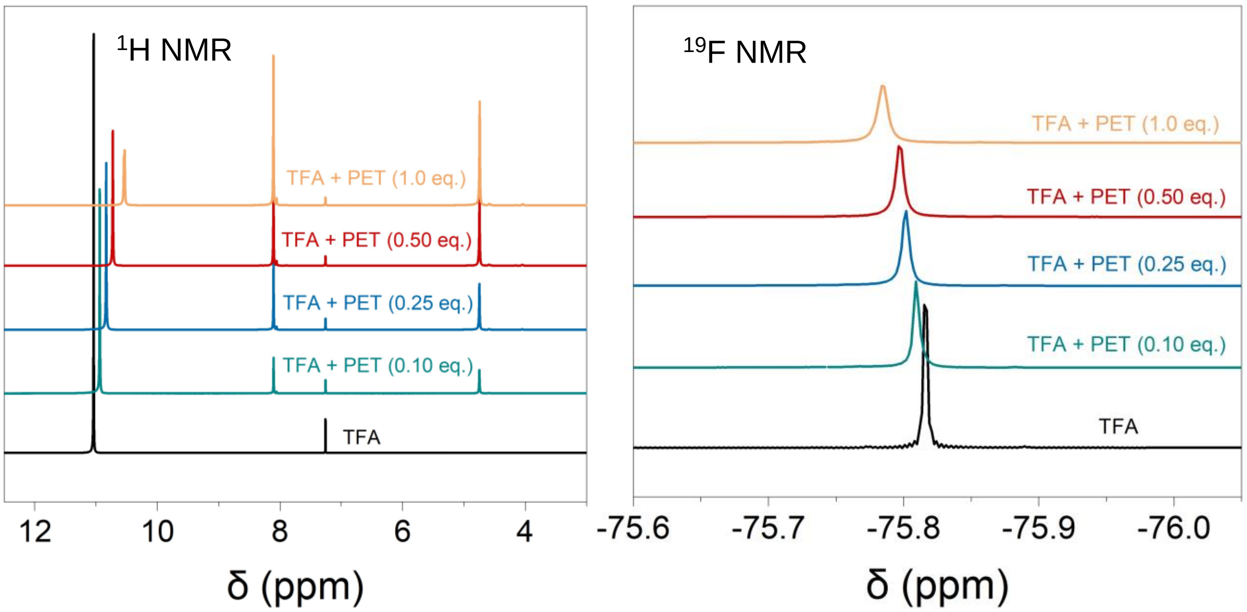

Notes: TFA (0.2 mmol, 15 µL, 1 equiv.) was added into $CDCl_3$ (1 mL), then different amounts of PET resin powder were added into $CDCl_3$ which containing TFA, with the increased of PET in the solvent, the active H characteristic peak (δ = 11.04 ppm) of TFA in ^{1}H NMR and the F atoms characteristic peak (δ = -75.81 ppm) of TFA in ^{19}F NMR was shifted.

2. ^{13}C NMR studies.

Figure S8. ^{13}C NMR spectra of PET-TFA

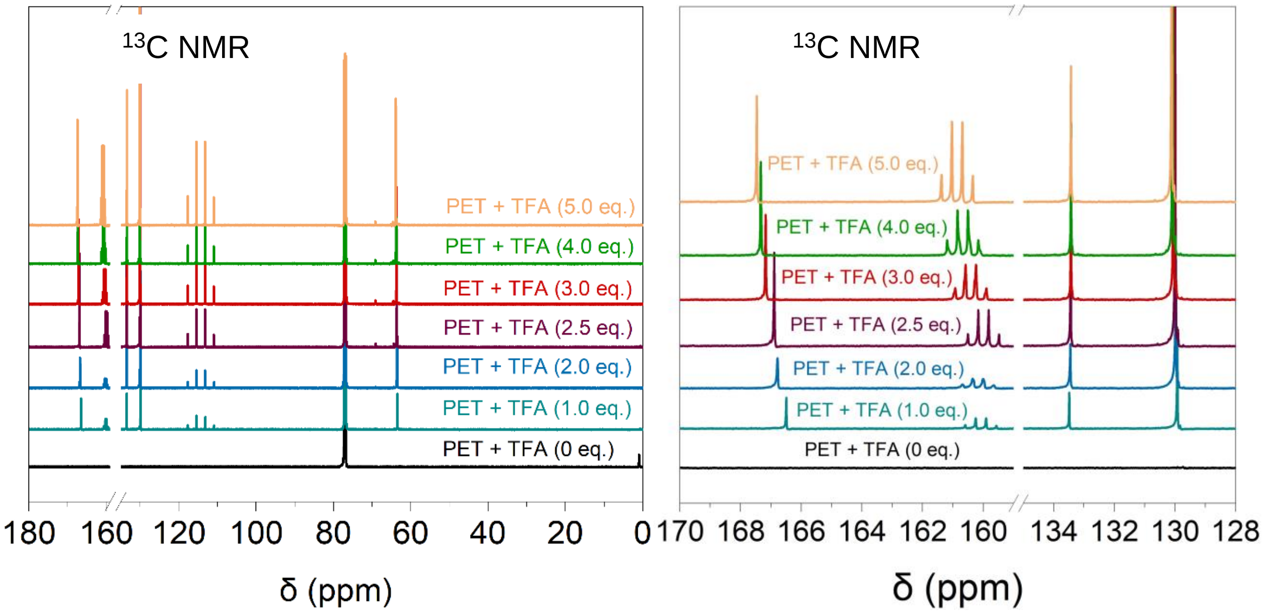

Notes: PET resin powder (48 mg, 0.25 mmol, 1 equiv.) was added into $CDCl_3$ (1 mL), then different amounts of TFA was added into $CDCl_3$, with the increased of TFA (0 - 3 equiv.) in $CDCl_3$, PET gradually dissolved in $CDCl_3$, PET was completely dissolved in $CDCl_3$ containing TFA (3 equiv.), Continue to increase the amount of TFA (3 - 5 equiv.), the carbonyl characteristic peak (δ = 166.49 ppm) of PET in ^{13}C NMR shifted toward lower field.

3. The stability of PET in TFA.

Figure S9. ^{1}H NMR studies of PET in TFA at different time.

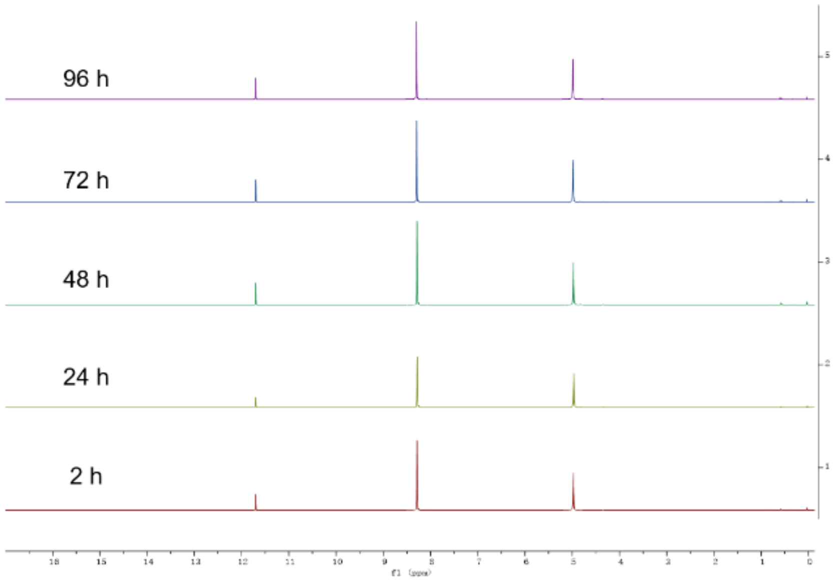

Figure S10. ^{13}C NMR studies of PET in TFA at different time.

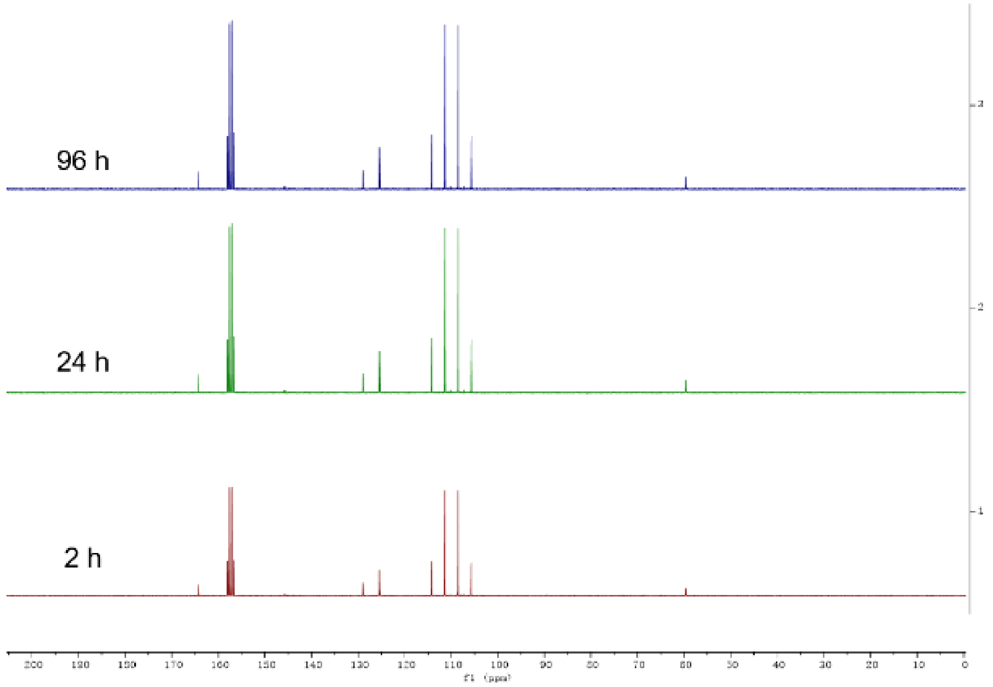

4. Gel Permeation Chromatography (GPC).

a). GPC curve of PET resin powder.

Calibration Type: Narrow Standard   Curve Fit Used: 3   K: 14.1000   Alpha: 0.7000

Calibration Curve: y = 19.937493 - 5.883792x^1 + 0.822949x^2 - 0.041422x^3

High Limit MW RT: 4.65 mins   Low Limit MW RT: 9.03 mins

Flow Marker RT: 0.00 mins   FRCF: 1.0000   FRM Name:

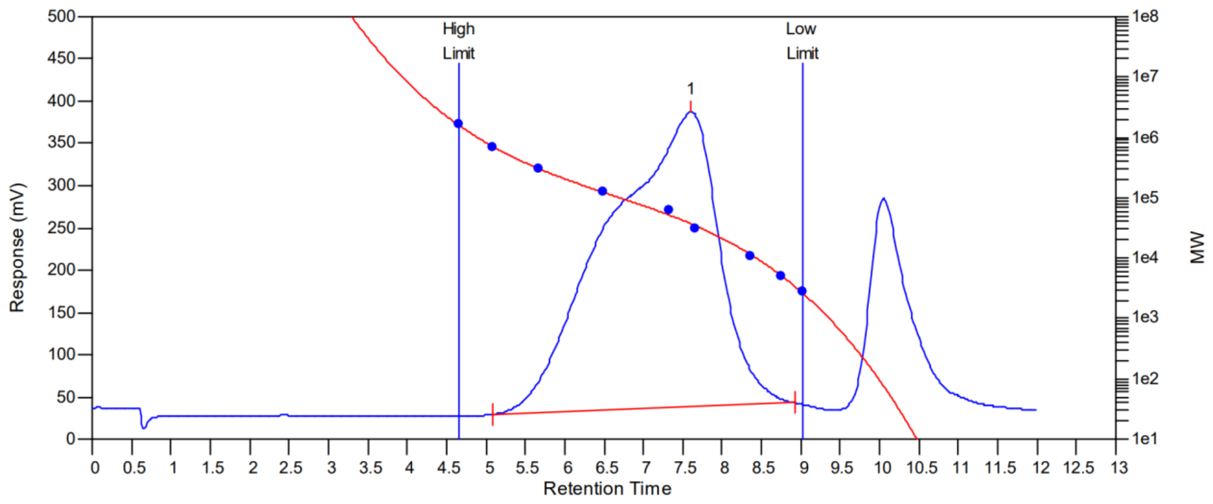

Figure S11a. GPC curves

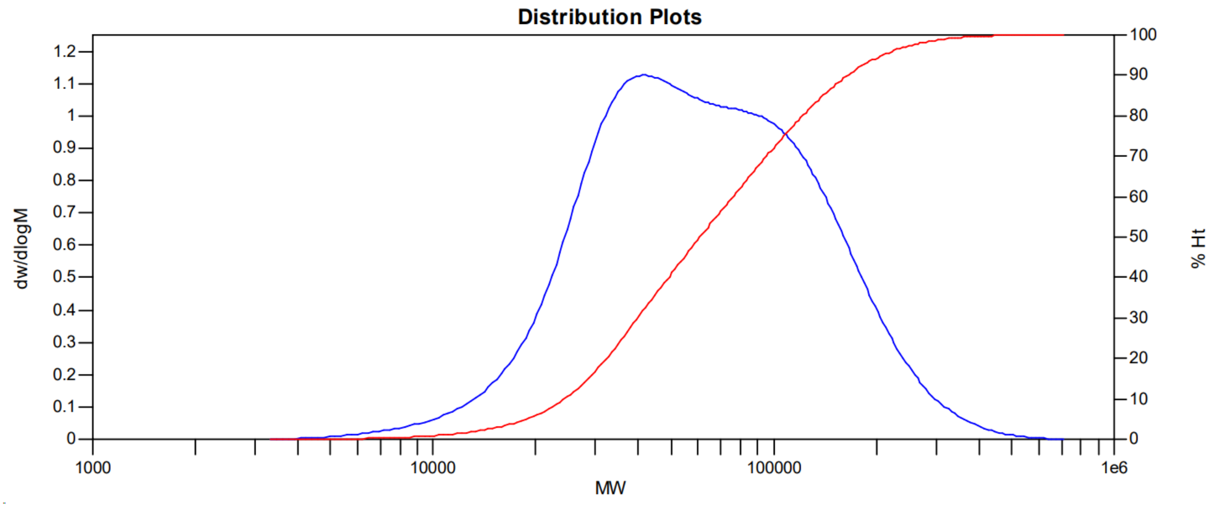

MW Averages：

Peak No	Mp	Mn	Mw	Mz	Mz+1	Mv	PD
1	37244	51730	84931	130725	185521	79197	1.64181

Processed Peaks：

Peak No	Name	Start RT	Max RT	End RT	Pk Height	%Height	Area %	Area
		(mins)	(mins)	(mins)	(mV)			(mV.secs)
1	5.08	7.60	8.93	349.177	0	34279.5	100	

b). GPC curve of TFA-treated PET.

Calibration Type: Narrow Standard Curve Fit Used: 3 K: 14.1000 Alpha: 0.7000

Calibration Curve: y = 19.937493 - 5.883792x^1 + 0.822949x^2 - 0.041422x^3

High Limit MW RT: 4.65 mins Low Limit MW RT: 9.03 mins

Flow Marker RT: 0.00 mins FRCF: 1.0000 FRM Name:

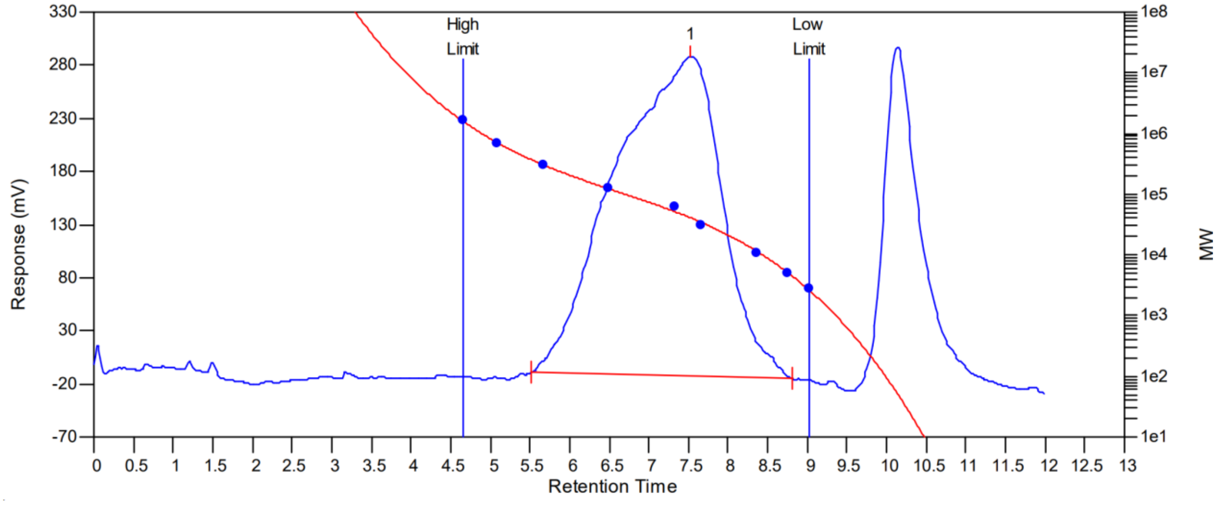

Figure S11b. GPC curves

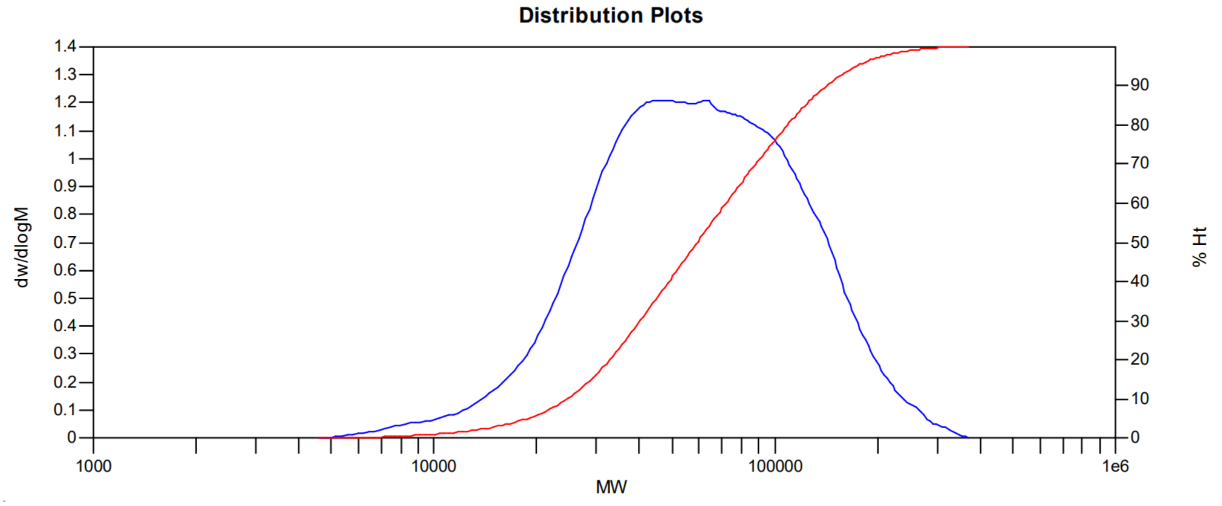

MW Averages：

Peak No	Mp	Mn	Mw	Mz	Mz+1	Mv	PD
1	41331	50900	77303	108563	141061	73075	1.51872

Processed Peaks：

Peak No	Name	Start RT (mins)	Max RT (mins)	End RT (mins)	Pk Height (mV)	%Height	Area %	Area (mV.secs)
1		5.52	7.52	8.80	301.081	0	28383.1	100

5. Scanning Electron Microscopy (SEM).

Figure S12. SEM of PET resin powder and TFA-treated PET.

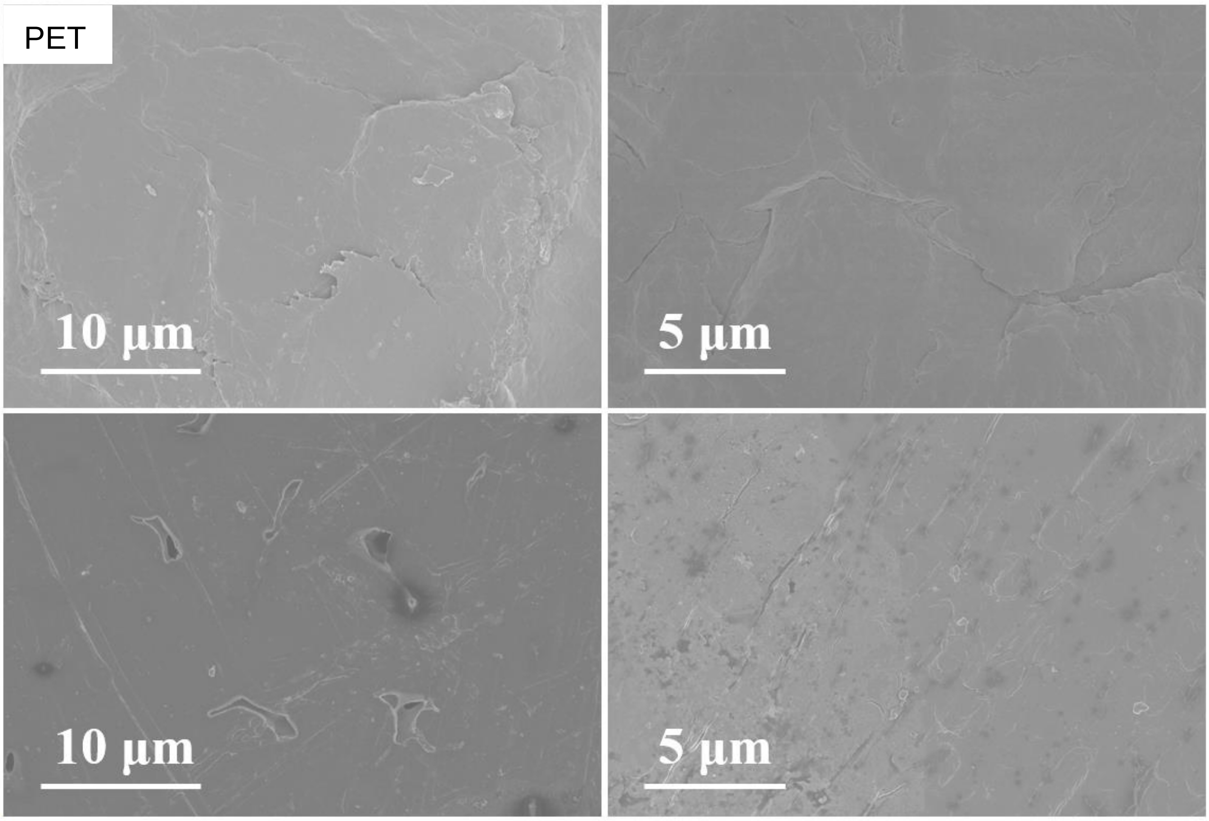
PET
10 µm
5 µm
10 µm
5 µm

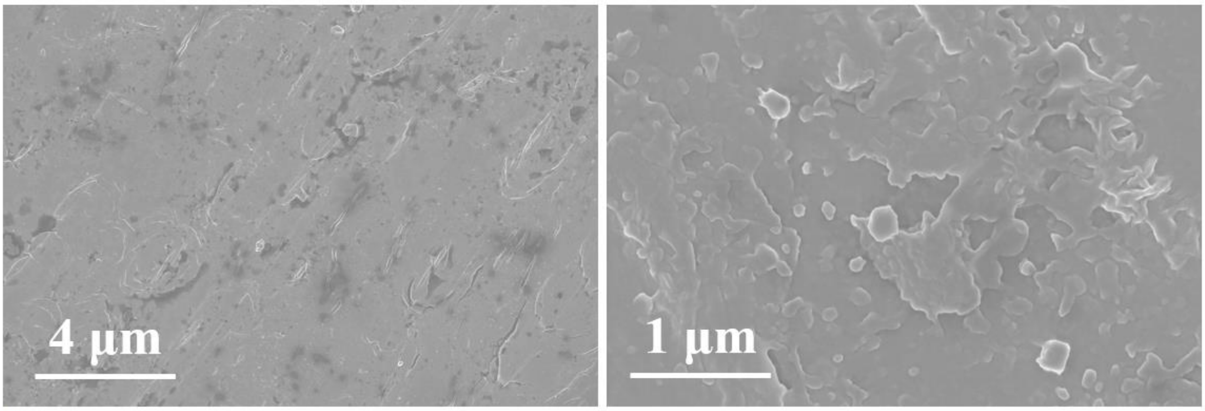
4 µm
1 µm

6. Energy Dispersive Spectrometer (EDS).

Figure S13. EDS of TFA-treated PET.

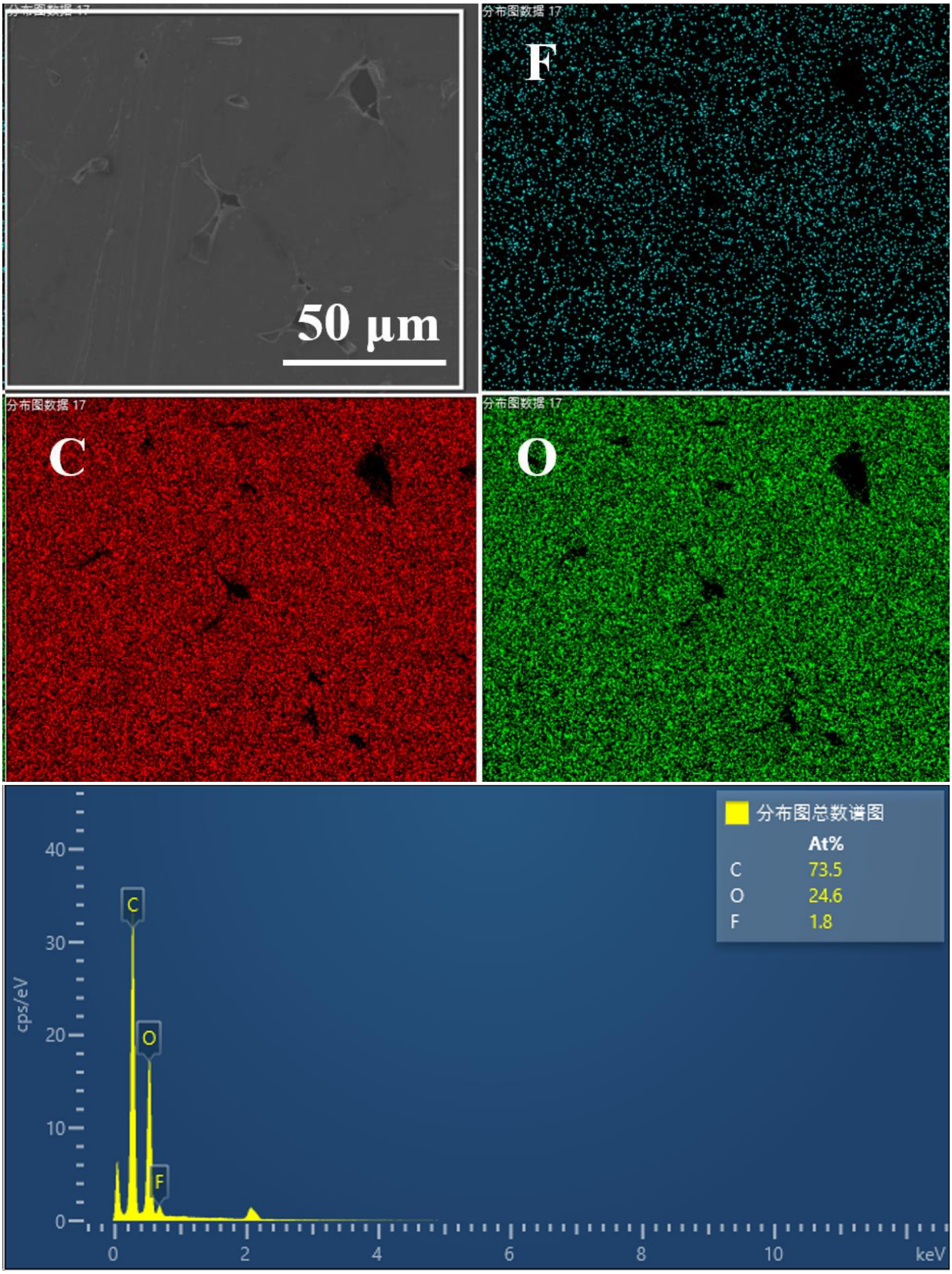

7. Thermal Gravimetric Analysis (TGA).

Figure S14. TGA curves

a). TGA curve of PET resin powder.

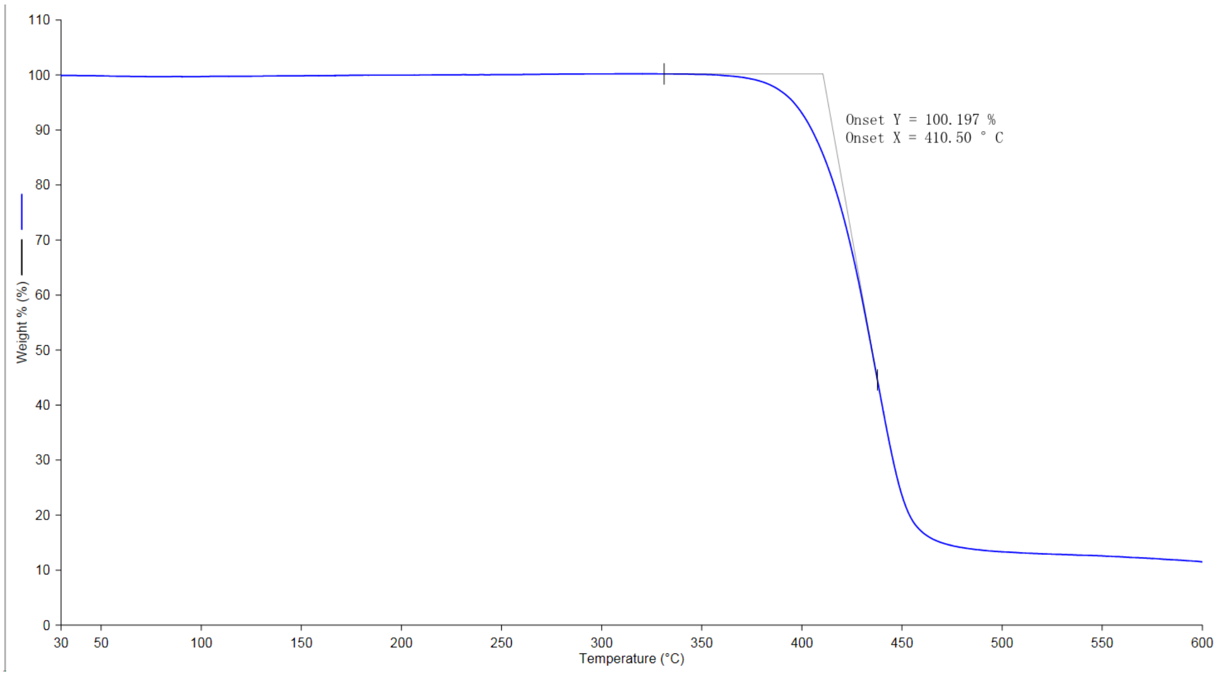

Notes: Sample Weight: 7.220 mg, Heat from 30.00°C to 620.00°C at 10.00°C/min, N_2.

b). TGA curve of TFA-treated PET.

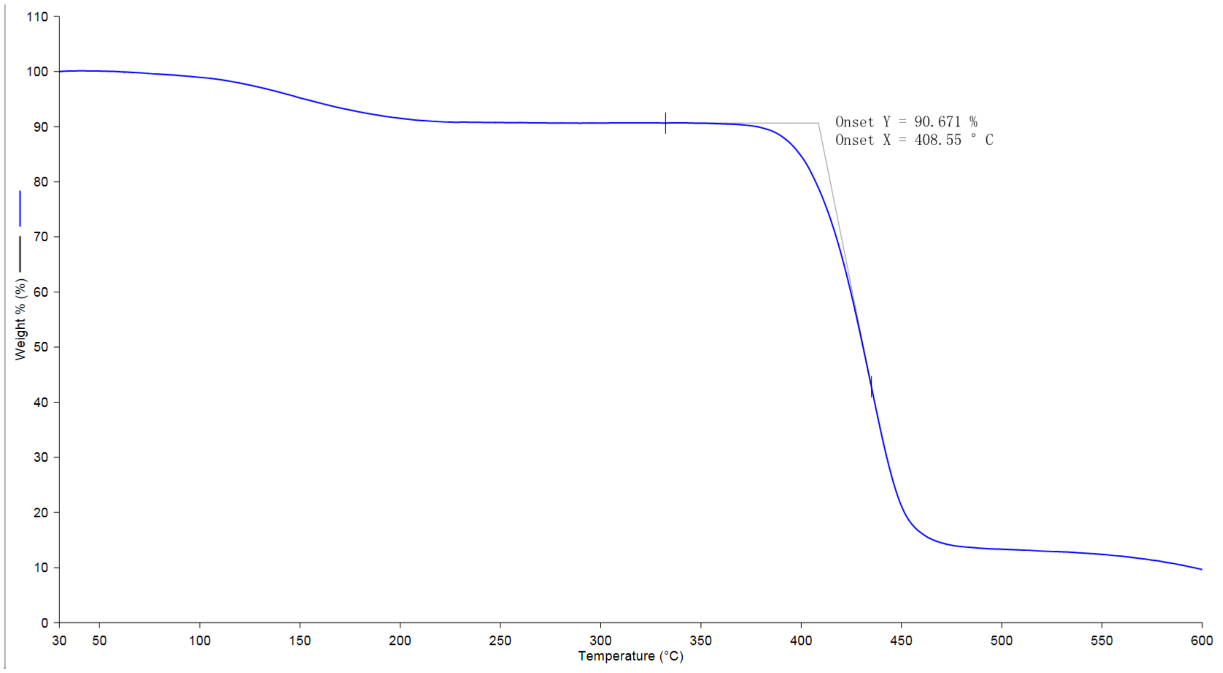

Notes: Sample Weight: 6.145 mg, Heat from 30.00°C to 620.00°C at 10.00°C/min, N_2.

8. Differential scanning calorimetry (DSC).

Figure S15. DSC curves.

a). DSC curve of PET resin powder.

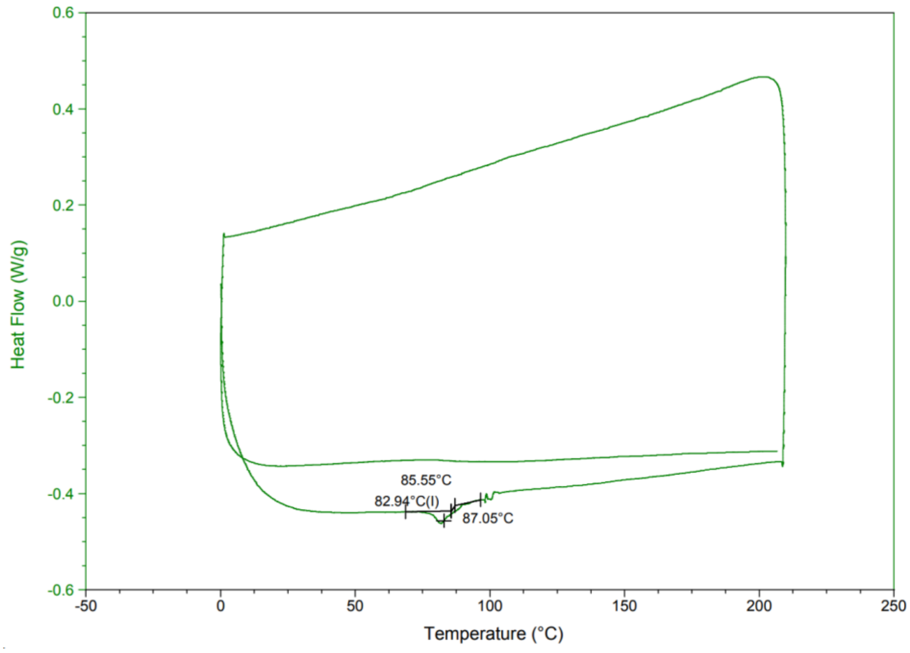

Notes: Sample Weight: 5.70000 mg, N_2 (50.0 mL/min). Method: 1). Equilibrate at 0.00°C; 2). Ramp 10.00°C/min to 210.00°C; 3). Isothermal for 3.00 min; 4). Ramp 10.00°C/min to 0.00°C; 5). Isothermal for 3.00 min; 6). Ramp 10.00°C/min to 210.00°C.

b). DSC curve of TFA-treated PET.

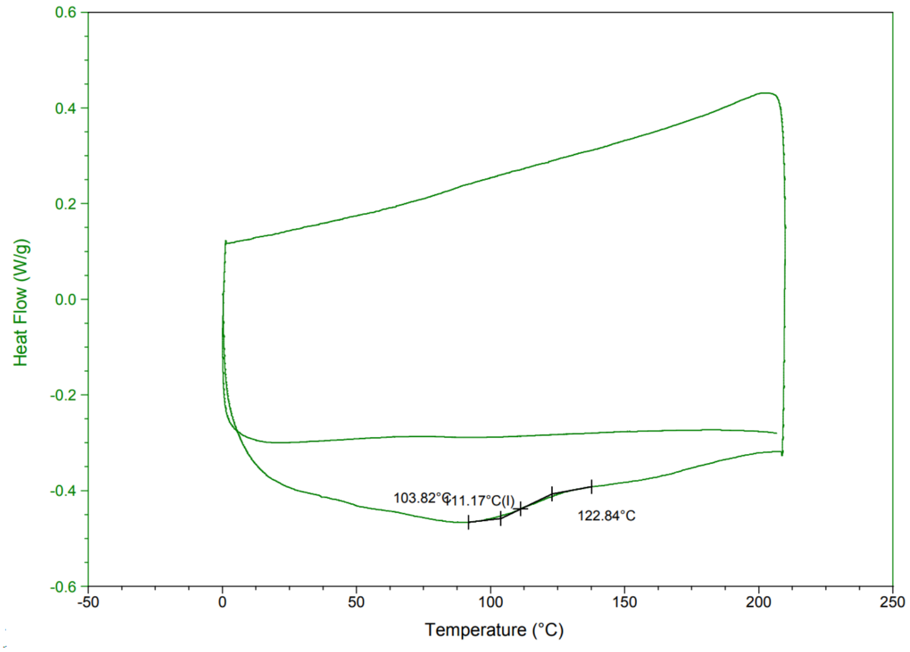

Notes: Sample Weight: 7.10000 mg, N_2 (50.0 mL/min). Method: 1). Equilibrate at 0.00°C; 2). Ramp 10.00°C/min to 210.00°C; 3). Isothermal for 3.00 min; 4). Ramp 10.00°C/min to 0.00°C; 5). Isothermal for 3.00 min; 6). Ramp 10.00°C/min to 210.00°C.

9. X-ray Diffraction (XRD).

Figure S16. XRD curves of PET resin powder and TFA-treated PET.

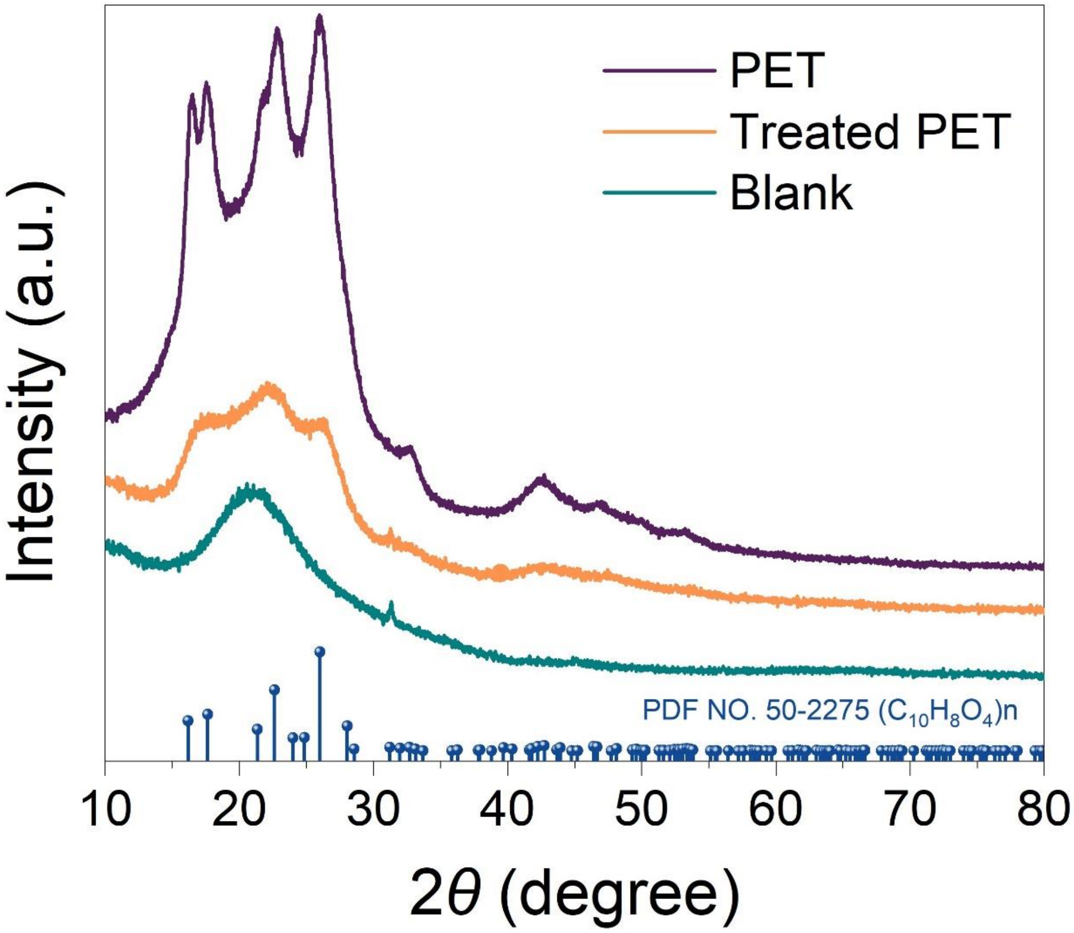

10. Fourier Transform infrared spectroscopy (FT-IR).

Figure S17. FT-IR curves

a). FT-IR curve of PET.

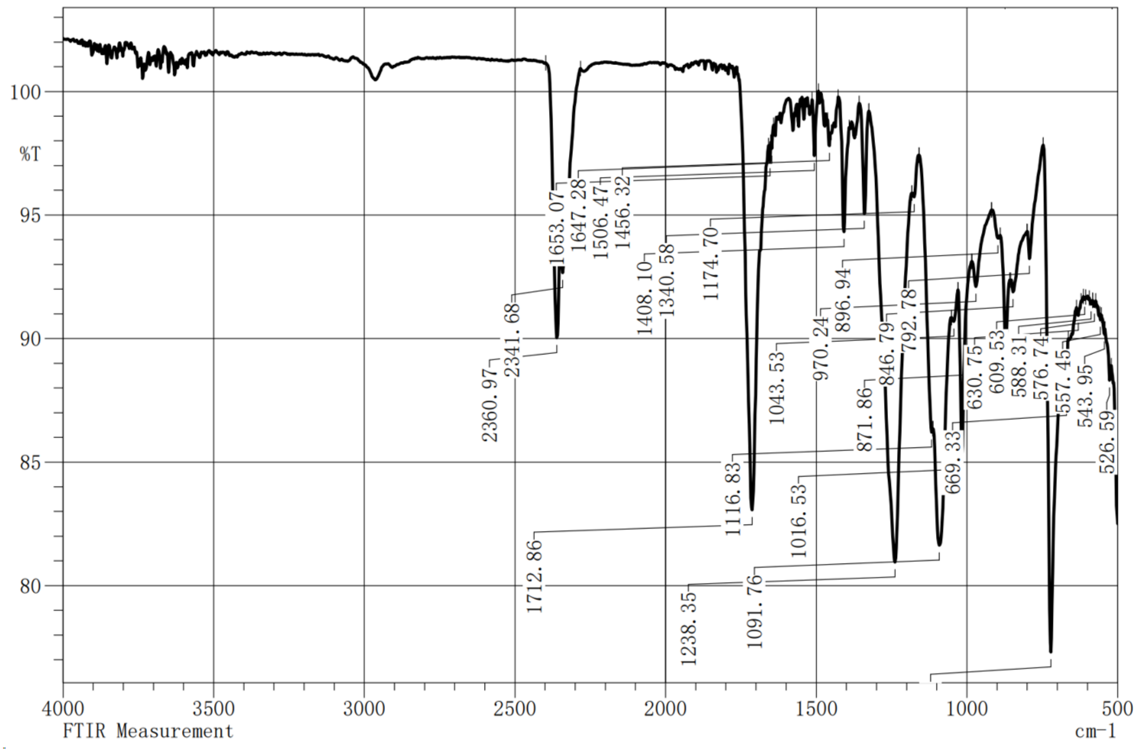

b). FT-IR curve of TFA-treated PET.

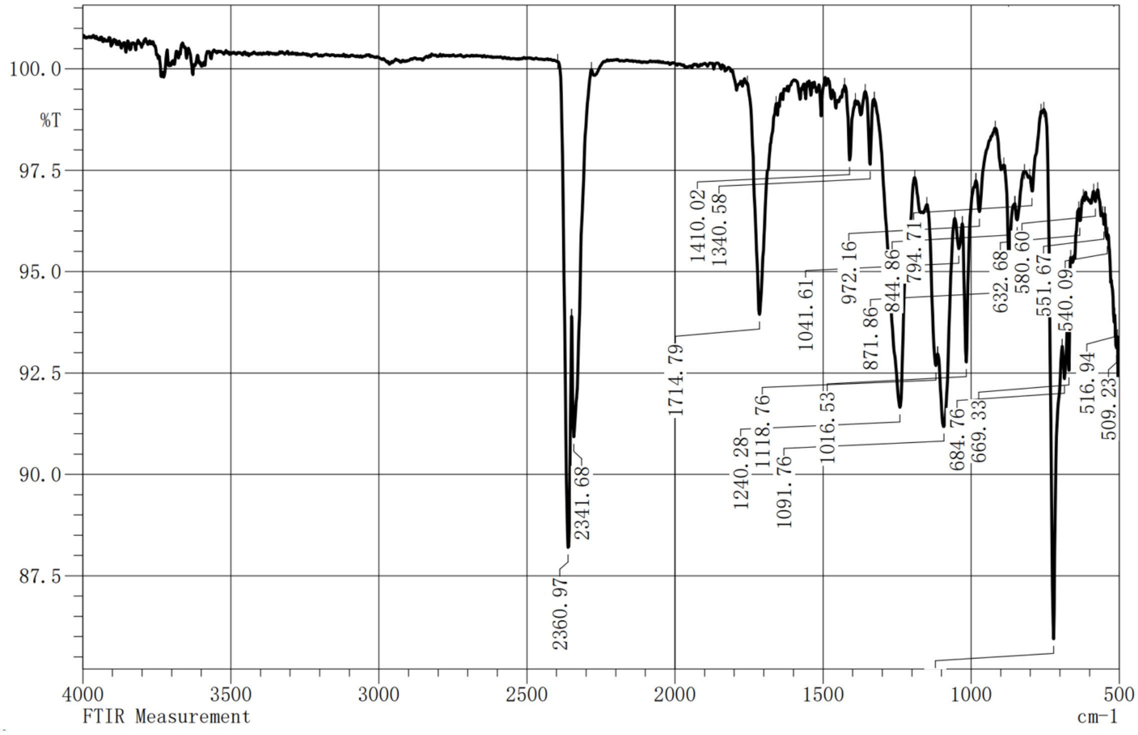

V. Mechanistic studies.

1). The effect of temperature on the reaction time.

Table S6. Depolymerization efficiency with different temperature and time.

Entry	Temperature (°C)	Pretreatment (min)	Depolymerization (min)	Yield (%)
1	20	120	350	97
2	30	70	170	98
3	40	40	80	98
4	50	30	50	98
5	60	20	40	99

2). Studies for the hydrolysis of polyester blends.

Figure S18. The morphology of PA_6 resin powder in reaction phenomena.

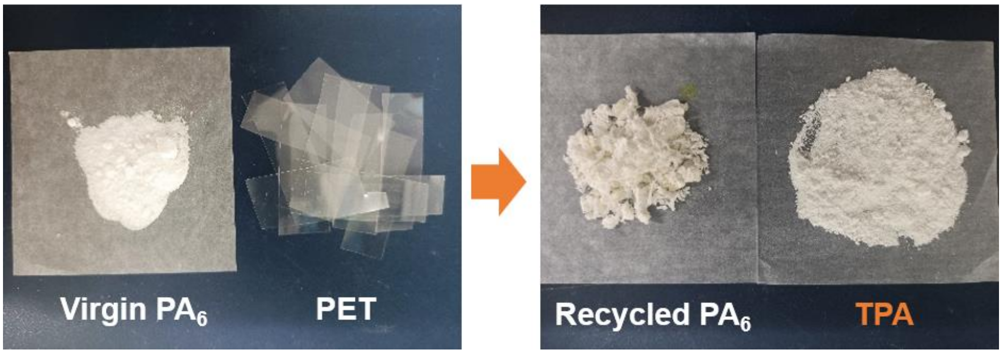

Figure S19. The ^1H NMR and ^{13}C NMR of recycled PA_6 and PA_6 virgin resin powder.

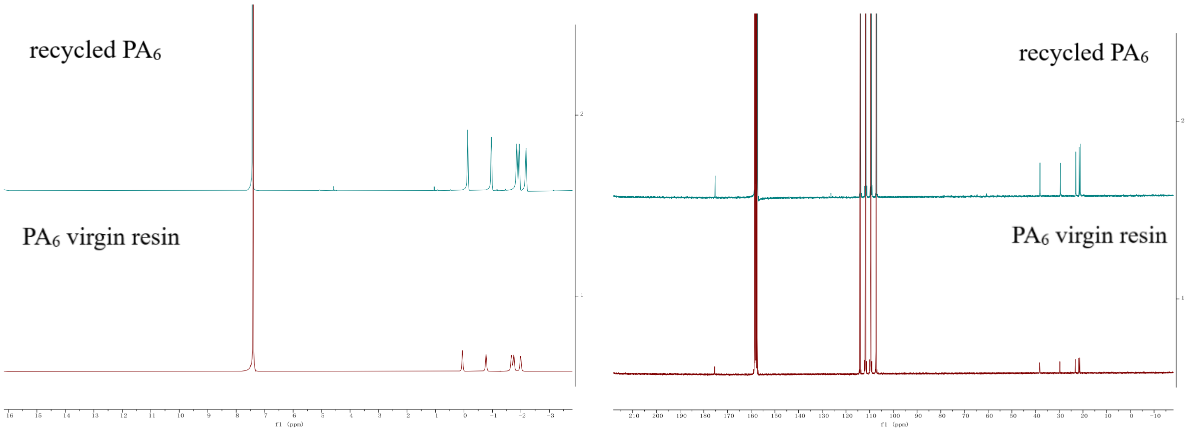

Figure S20. The ^{1}H NMR and ^{13}C NMR spectra of the recycled BPA from PET/PC and PBT/PC blends.

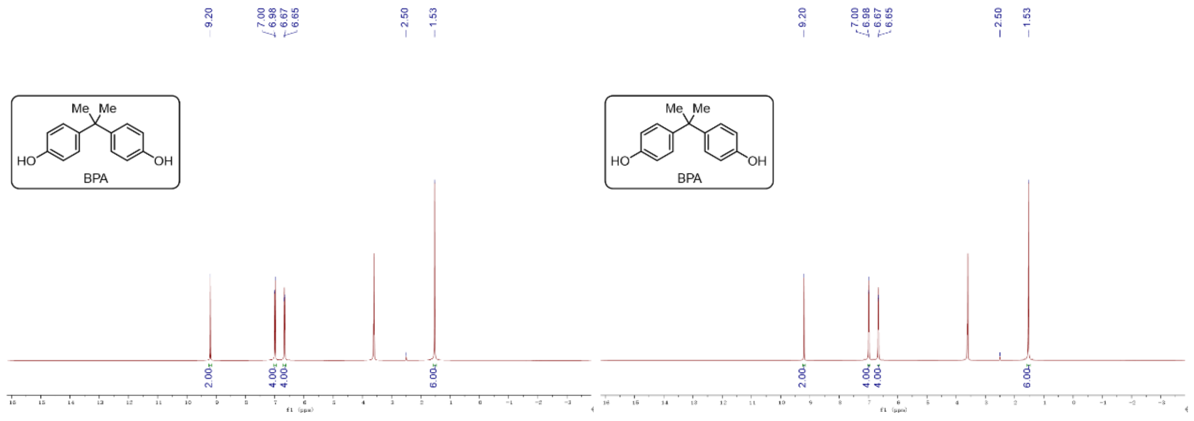

Figure S21. The XRD studies of the recycled PE/virgin PE resin (left) and the recycled PP/virgin PP resin (right).

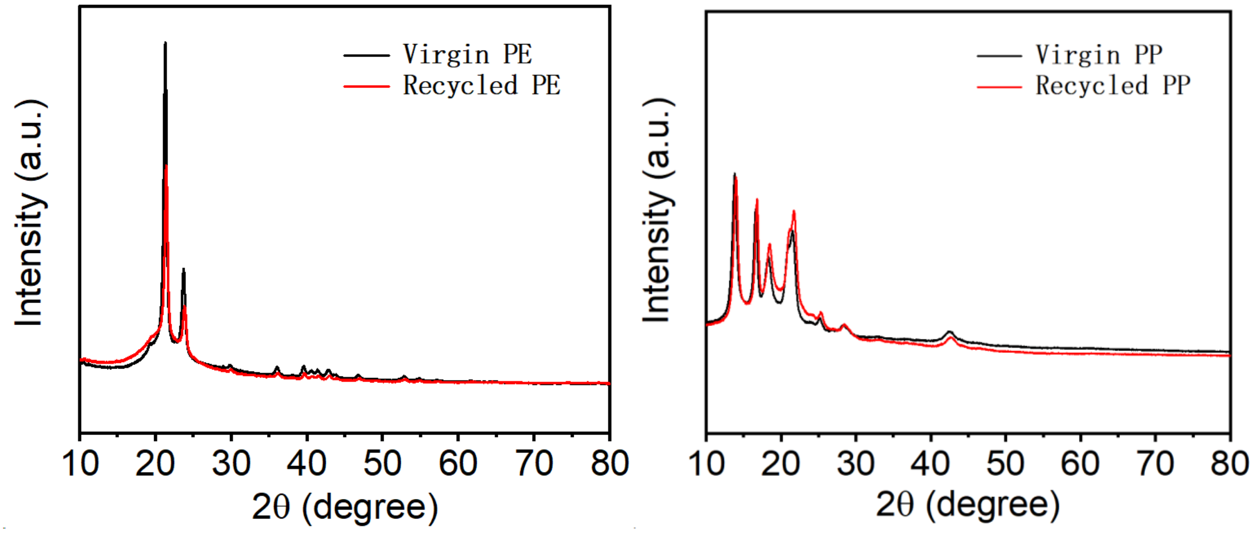

Figure S22. The changes in reaction phenomena of the PET/PE and PET/PP blends.

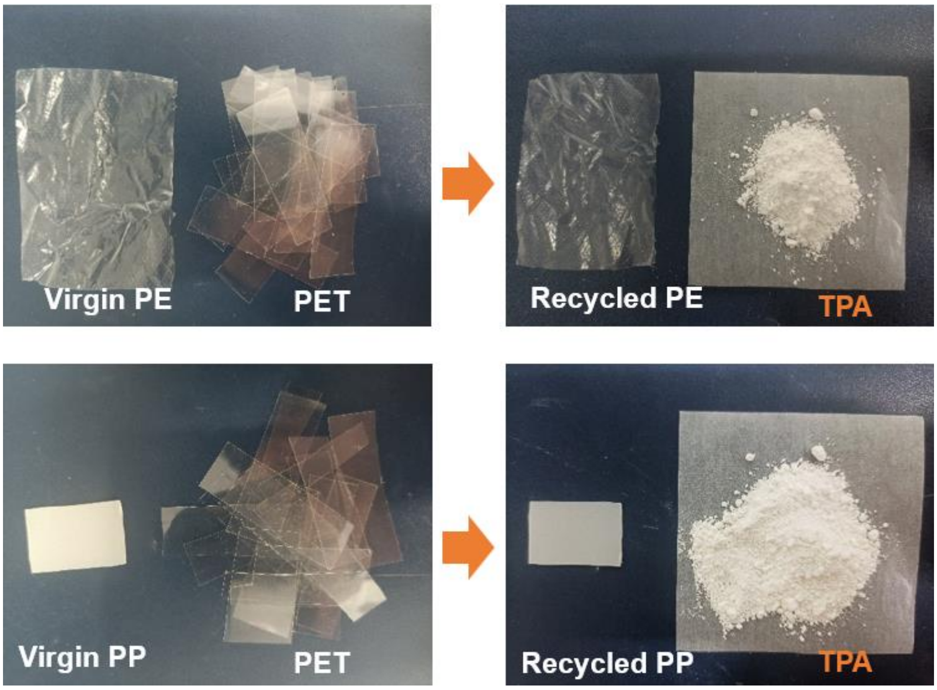

Figure S23. The depolymerization processes of PET for hydrolysis and aminolysis.

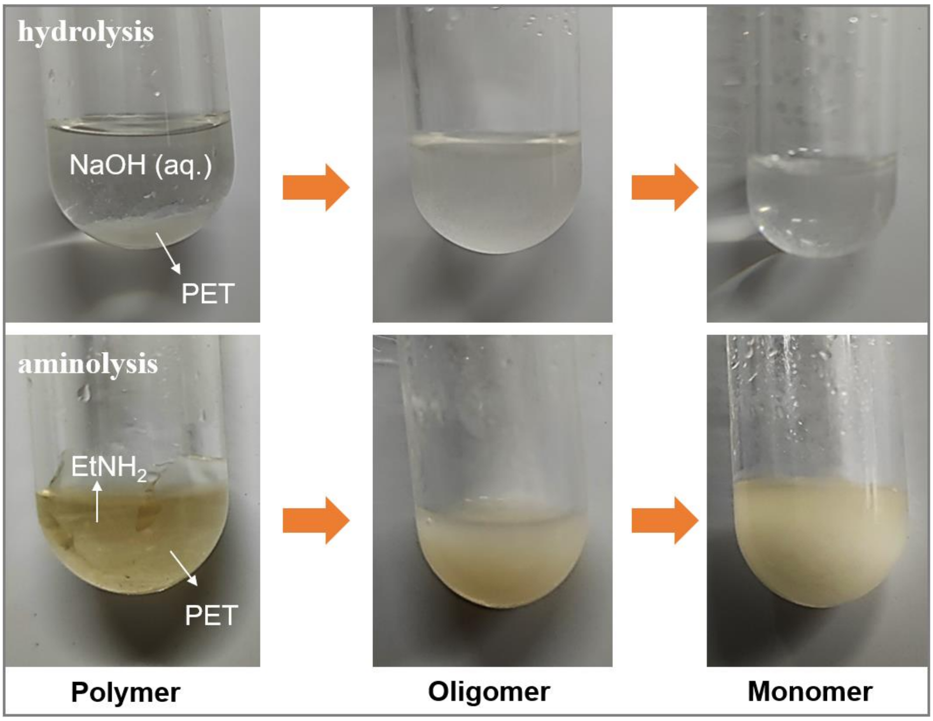

Table S7. The yields of the salt and **5d** with different amounts of TBD.

**Entry**	**TBD** (equiv.)	**Time** (h)	**Yield** of TBD/TFA salt (%)	**Yield** of **5d** (%)
1	-	24	-	trace
2	-	72	-	trace
3	10 mol%	24	65	trace
4	50 mol%	24	80	trace
5	1.0	24	93	trace
6	2.0	24	74	< 10
7	3.0	24	45	46
8	3.0	72	46	76

Table S8. The yields of the salt and **5g** with different amounts of TBD.

**Entry**	**TBD** (equiv.)	**Time** (h)	**Yield** of TBD/TFA salt (%)	**Yield** of **5g** (%)
1	-	24	-	trace
2	-	72	-	trace
3	10 mol%	24	60	trace
4	50 mol%	24	77	trace
5	1.0	24	86	trace
6	2.0	24	68	22
7	3.0	24	37	52
8	3.0	72	35	87

Figure S24. ^{1}H NMR (left), ^{19}F NMR (middle), and ^{13}C NMR (right) spectra of TFA-TBD salt.

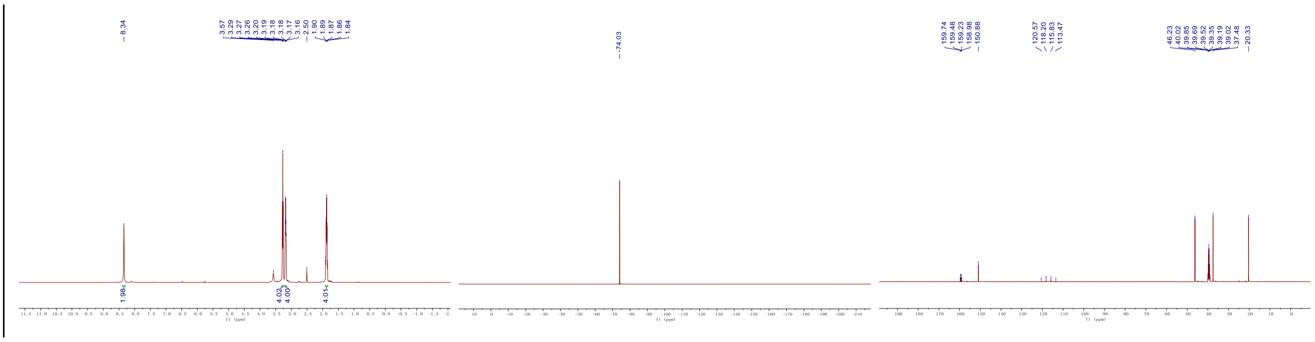

Table S9. The experiments of methanolysis and glycolysis of PET resin powder.

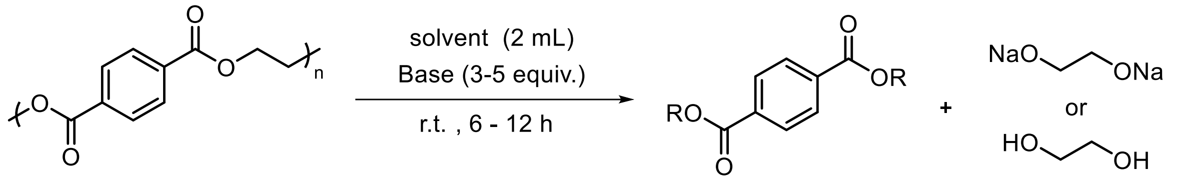

PET film (48 mg)

**Entry**	**PET source**	**Base** (equiv.)	**Solvent**	**Yield** (%)
1	TFA-treated PET	MeONa (3.0)	DCM	N.D.
2	TFA-treated PET	MeONa (3.0)	MeOH	N.D.
3	TFA-treated PET	MeONa (3.0)	DCM+MeOH	< 10
4	TFA-treated PET	EtONa (3.0)	DCM+EtOH	< 10
5	TFA-treated PET	EtONa (3.0)	EG	N.D.

DFT calculation and computational details.

a). DFT calculation.

All calculations were performed with the Gaussian 16 package[1]. Geometry optimizations were performed with B3LYP-D3(BJ)[2] set with the PCM[3] (water) solvation model. The 6-31+G(d,p)[4] basis set was used for all atoms. All optimized structures were verified by frequency calculations and only one imaginary frequency was found in the transition states, while the other structures had no imaginary frequency. Besides, the thermal correction to Gibbs free energy were obtained after frequency calculations. Intrinsic Reaction Coordinate (IRC)[5] was utilized to confirm the reaction pathway. In addition, the single point calculations considering solvation effect were performed with M06-2X-D3[6] and 6-311+G(d,p)[7] basis set with the SMD[8](water) solvation model. All optimized structures were visualized using VMD 1.9.3[9] program. The weak interactions were visualized using Multiwfn_3.8[10] with independent gradient model based on Hirshfeld partition (IGMH)[11] method. The binding energies were performed with M06-2X-D3 and 6-311+G(d,p).

The PET crystal structure was referred to the literature (*ACS Omega* **2018**, *3*, 4824-4835),[12] The simple model used dimethyl terephthalate (DMT) to investigate the interaction between TFA and PET. Using the genmer program to sample TFA surrounding DMT, and the structures were optimized using GFN0-xTB[13] method and B3LYP-D3(BJ) method via the molclus program.[14] In molecular dynamics simulations of TFA dissolving PET, a 3*3*3 PET model was used with a system composed of 500 TFA molecules. The sobtop program[15] was employed to construct topology files, selecting GAFF (General AMBER Force Field) atom types and force field parameters. MMFF94 charges were calculated using the OpenBabel program, and the gromos54a7 force field was chosen. A 100 ns constant temperature and pressure dynamics simulation using the gromacs software[16] under the NPT ensemble. To explore the environment in which TFA exists within the complex, a 1:2 complex by constructing 10 PET chains, each consisting of 10 repeating units, interacting with 200 TFAs. The system was equilibrated under the NVT ensemble.

Figure S25. Visualization of weak interactions between molecular chains in the PET crystalline region.

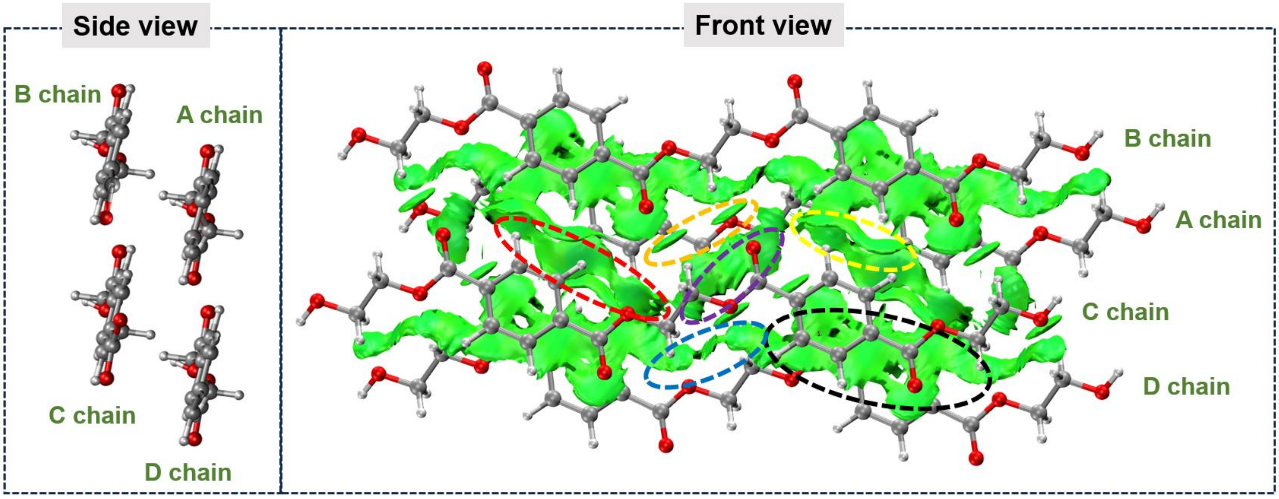

Notes：**Red region**：between A and C chain, H --- H interaction (Ar-H --- H-Ar, van der Waals) and H --- O interaction (Ar-H --- OCH_2, weak Hydrogen-bonding); **Orange region**：between A and D chain, H --- O interaction (C*sp*^{3}-H --- O=C, weak Hydrogen-bonding); **Yellow region**：between A and D chain, H --- H interaction (Ar-H --- H-Ar, van der Waals); **Purple region**：between A and C chain, H --- O interaction (C*sp*^{3}-H --- O=C, weak Hydrogen-bonding); **Blue region**：between A and B chain, H --- H interaction (C*sp*^{3}-H --- H- C*sp*^{3}, van der Waals) and C*sp*^{3}-H --- OCH_2 (weak Hydrogen-bonding); **Black region**：between A and B chain, π-π stock.

Figure S26. The strength of repeated unit interactions between PET molecular chains.

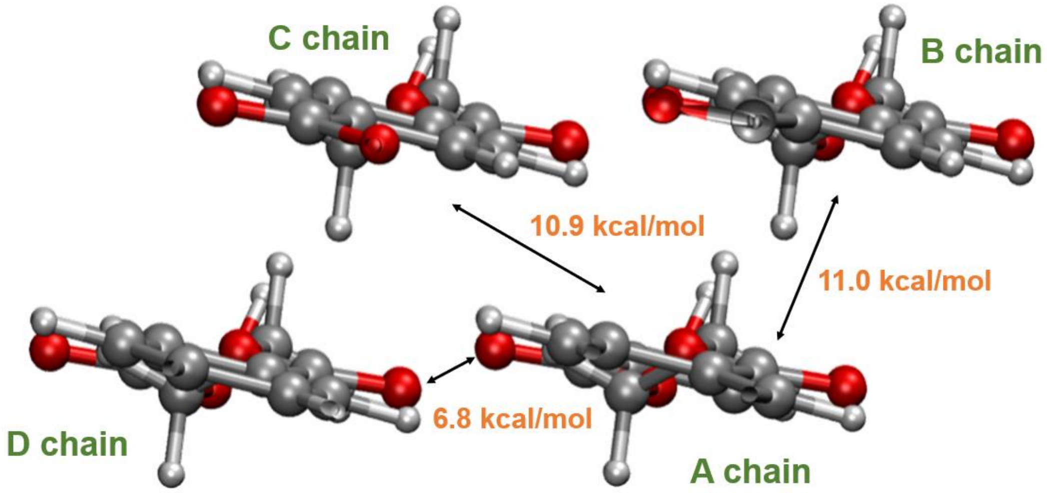

Notes: The binding energy between the repeated unit of PET chains is calculated according to the following formula.

BE (A --- B chain) = BE (PET94-B) - BE (PET50-B) = 11.0 kcal/mol

BE (A --- C chain) = BE (PET94-C) - BE (PET50-C) = 10.9 kcal/mol

BE (A --- D chain) = BE (PET94-D) - BE (PET50-D) = 6.8 kcal/mol

Table S10. The binding energy between PET chain and TFA.

	E(kcal/mol)	BE(kcal/mol)
**PET25**	-763.152172	/
**PET50-B**	-1526.321685	10.9
**PET50-C**	-1526.313905	6.0
**PET50-D**	-1526.311879	4.7
**PET47**	-1449.886745	/
**PET94-B**	-2899.808440	21.9
**PET94-C**	-2899.800415	16.9
**PET94-D**	-2899.791922	11.6

Figure S27. The possible binding sites of PET and TFA by DFT calculation.

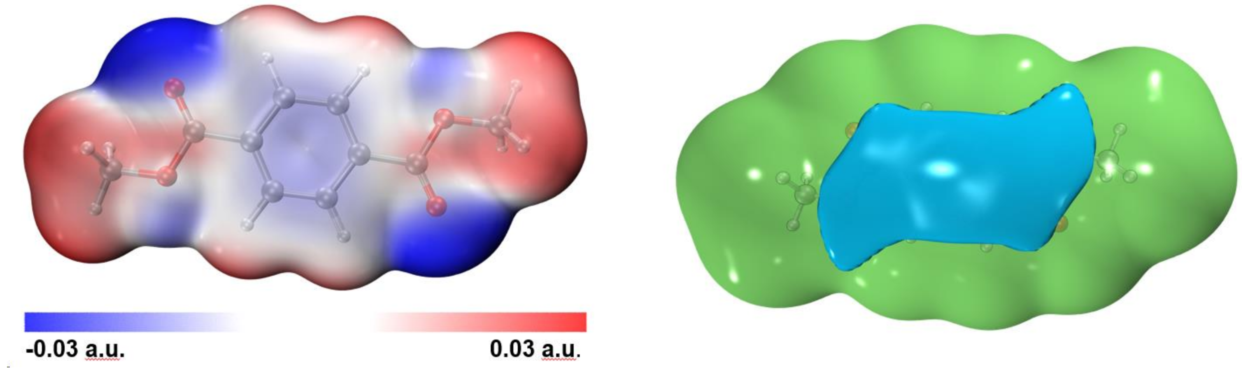

Notes: The molecular surface electrostatic potential of DMT (**Left**, the blue region are possible hydrogen bonding sites); The molecular surface van der Waals potential of DMT (**Right**, the cyan region are possible van der Waals binding sites).

Figure S28. Interaction pattern and strength between PET chain and TFA (1:1 model).

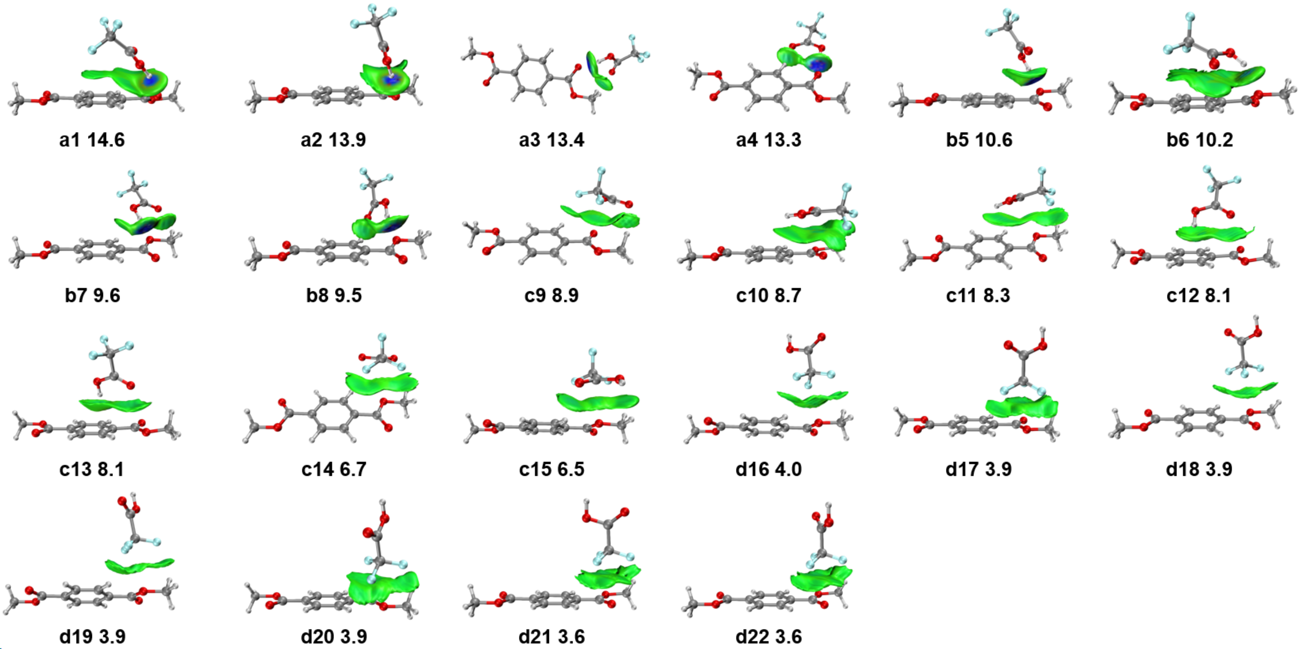

Notes: The binding energy between DMT and TFA is calculated according to formula (1)

$$BE = E(DMT\bullet TFA) - E(DMT\text{---}TFA).$$

BE is the binding energy between DMT and TFA; E(DMT•TFA) is the single point energy near the distance between DMT and TFA; E(DMT---TFA) is the single point energy with a long distance between DMT and TFA.

Table S11. The binding energy between PET chain and TFA (1:1 model).

	**E(DMT•TFA) (kcal/mol)**	**E(DMT---TFA) (kcal/mol)**	**BE (kcal/mol)**
**a1**	-1214.710177	-1214.733442	14.6
**a2**	-1214.711063	-1214.733263	13.9
**a3**	-1214.711476	-1214.732868	13.4
**a4**	-1214.711730	-1214.732880	13.3
**b5**	-1214.711457	-1214.728311	10.6
**b6**	-1214.709729	-1214.725936	10.2
**b7**	-1214.711662	-1214.726946	9.6
**b8**	-1214.711729	-1214.726850	9.5
**c9**	-1214.712344	-1214.726499	8.9
**c10**	-1214.712615	-1214.726557	8.7
**c11**	-1214.712700	-1214.725986	8.3
**c12**	-1214.712964	-1214.725909	8.1

**c13**	-1214.713099	-1214.725958	8.1
**c14**	-1214.712834	-1214.723549	6.7
**c15**	-1214.712580	-1214.722960	6.5
**d16**	-1214.713540	-1214.719927	4.0
**d17**	-1214.713548	-1214.719805	3.9
**d18**	-1214.713535	-1214.719775	3.9
**d19**	-1214.713499	-1214.719663	3.9
**d20**	-1214.713499	-1214.719663	3.9
**d21**	-1214.713364	-1214.719156	3.6
**d22**	-1214.713348	-1214.719066	3.6

Figure S29. Molecular dynamics simulation of TFA dissolving PET.

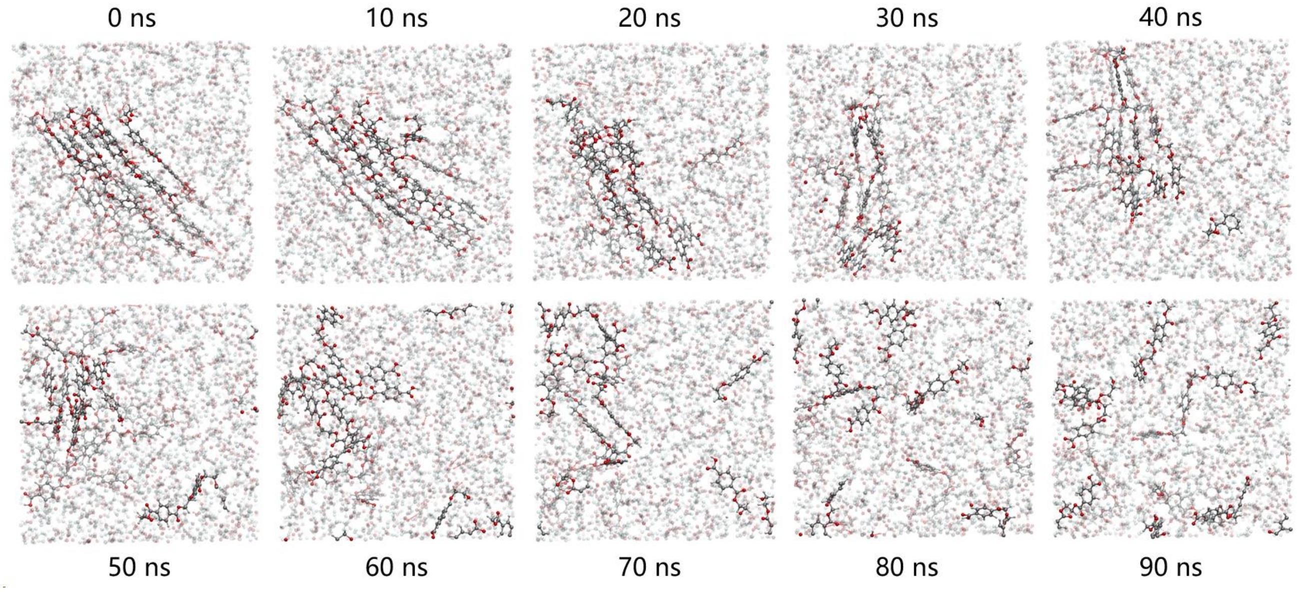

Figure S30. The number variation of hydrogen bonds formed between PET and TFA over time.

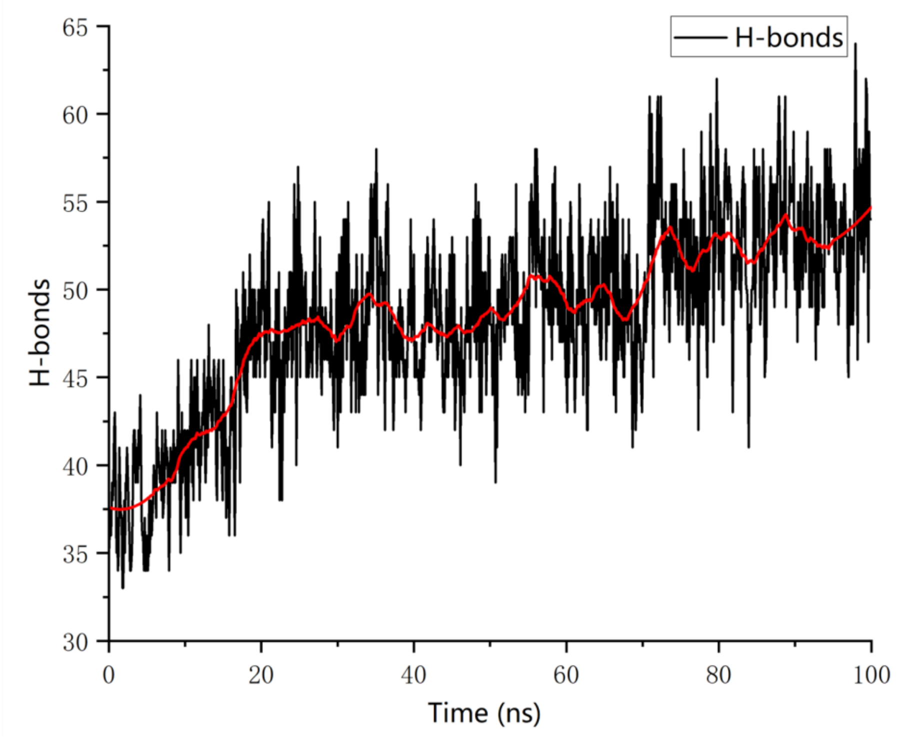

Figure S31. The variation of RMSD (Root Mean Square Deviation) of PET over time.

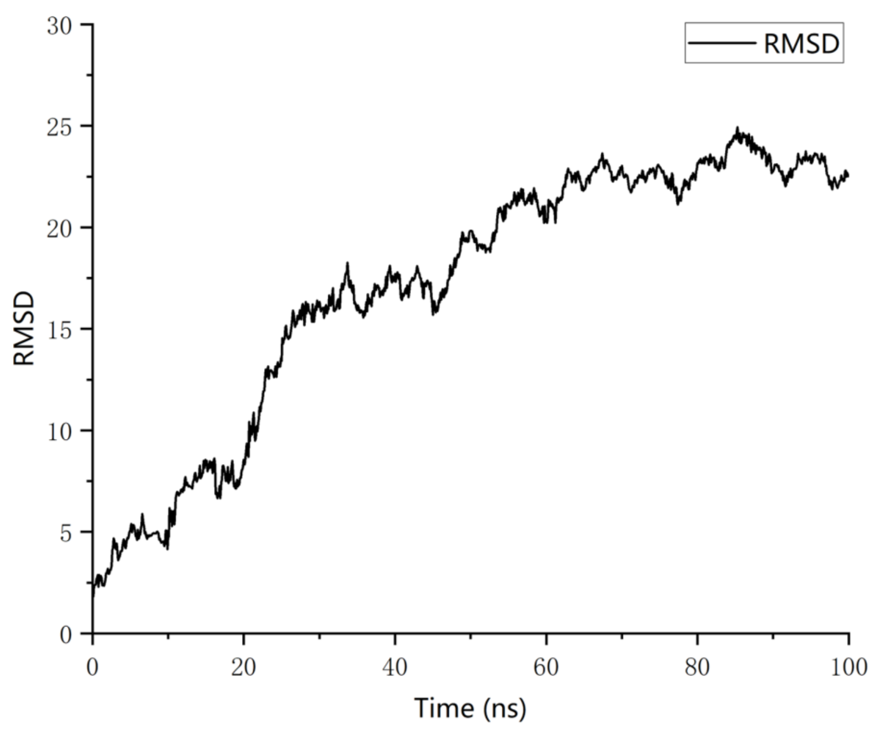

Figure S32. PET chains and TFA interact in three ways in PET/TFA complex.

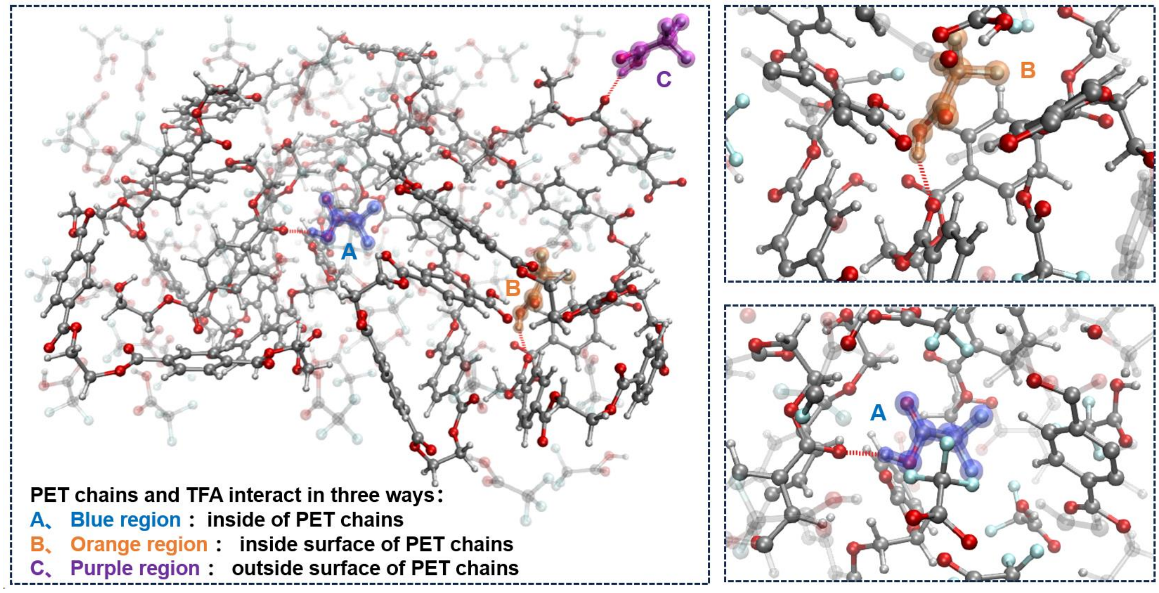

Figure S33. DFT calculation for conjugated base activate the nucleophile.

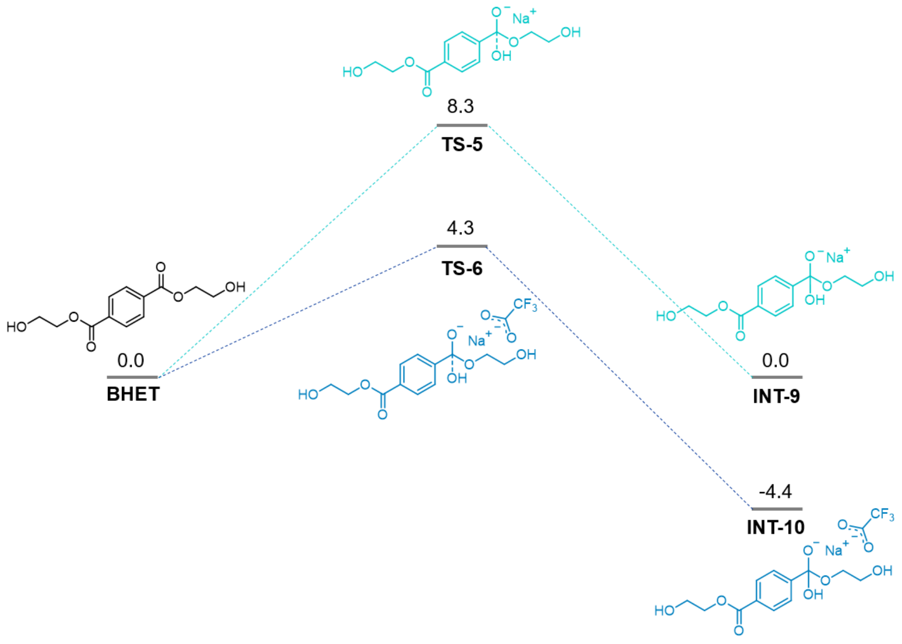

Figure S34. DFT calculations for dynamics in the depolymerization process.

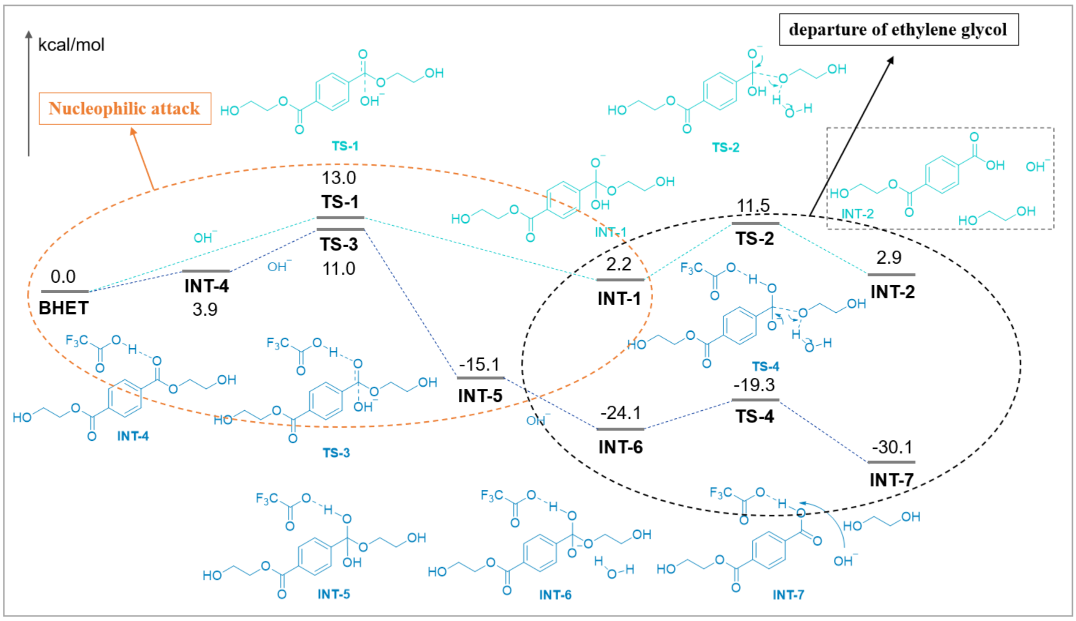

Figure S35. Hydrogen bonding was visualized.

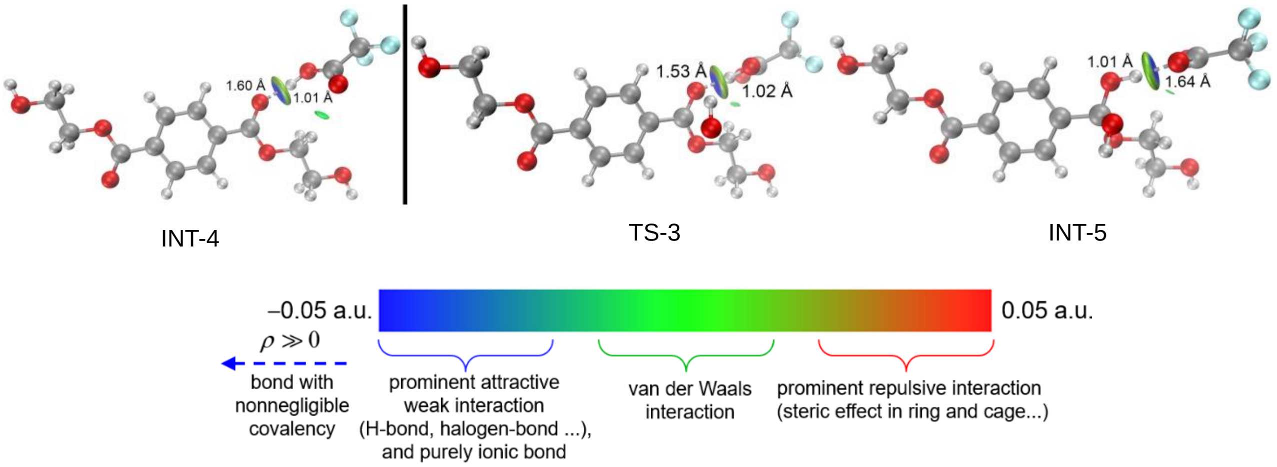

Table S12. Hydrogen bonding energy.

	INT-4	TS-3	INT-5
Bonding Energy	-7.6 kcal/mol	-7.4 kcal/mol	-6.5 kcal/mol

Table S13. Proposed classification of intermolecular hydrogen bonding.[17]

Type of complex	Strength	BE	Major nature
Neutral	Very weak	> −2.5 kcal/mol	Dispersion + Electrostatics
	Weak to medium	−2.5 to −14.0 kcal/mol	Electrostatics
Charged	Medium	−11.0 to −15.0 kcal/mol	Electrostatics
	Strong	< −15.0 kcal/mol	Electrostatics + Induction

Table S14. Calculated energy data and imaginary frequencies for all structures.

	Imaginary frequency	H（T）(Hartree）	G（T）(Hartree)	ΔG（kcal/mol）
**BHET+H_2O+OH^-**	none	-1069.059206	-1069.169937	0.0
**TS-1**	-105.1	-1069.054125	-1069.149207	13.0
**INT-1**	none	-1069.071561	-1069.166394	2.2
**TS-2**	-225.77	-1069.071523	-1069.151535	11.5
**INT-2**	none	-1069.052901	-1069.165327	2.9
**INT-3**	none	-1069.095334	-1069.209793	-25.0
**BHET+CF_3COOH+2*HO^-**	none	-1595.327699	-1595.475323	0.0
**INT-4**	none	-1595.337303	-1595.469161	3.9
**TS-3**	-118.27	-1595.340917	-1595.457769	11.0

**INT-5**	none	-1595.385286	-1595.499464	-15.1
**INT-6**	none	-1595.412299	-1595.513797	-24.1
**TS-4**	-247.94	-1595.403982	-1595.506097	-19.3
**INT-7**	none	-1595.389873	-1595.523304	-30.1
**INT-8**	none	-1595.422243	-1595.574943	-62.5

Figure S36. Calculated reaction pathways.

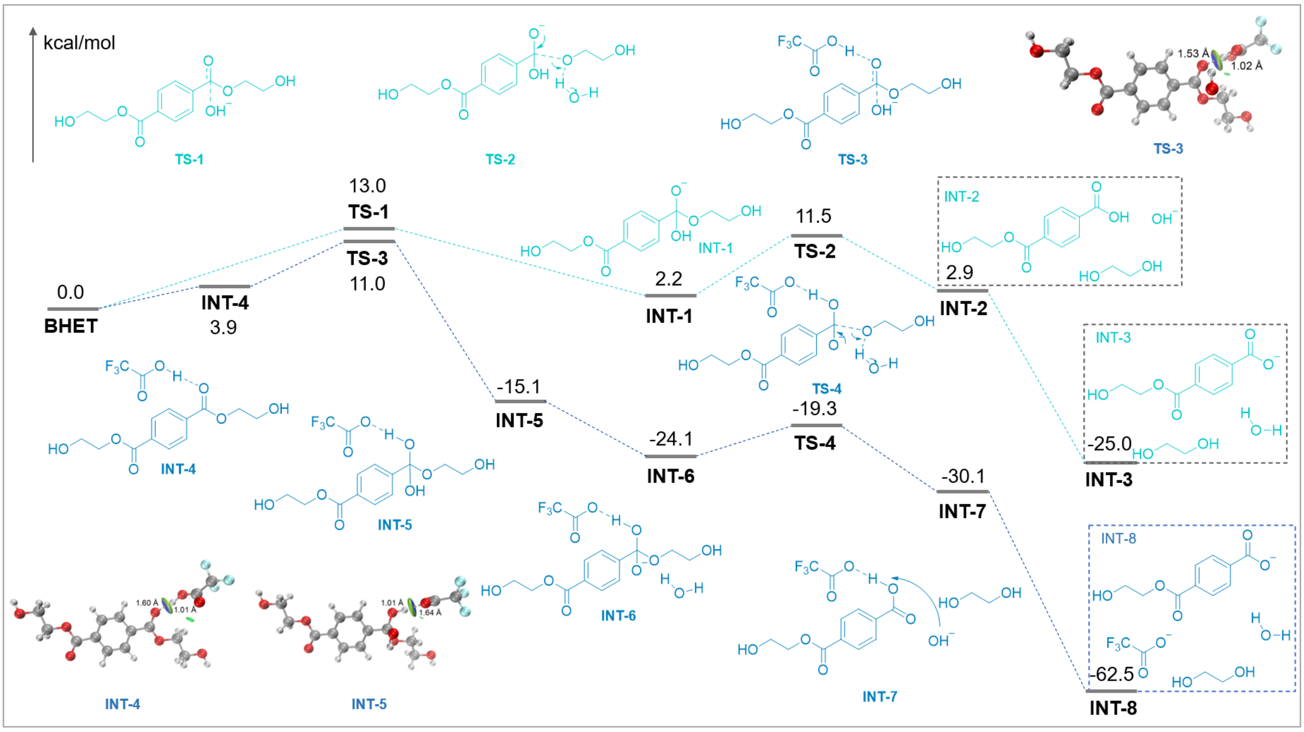

b). Cartesian coordinates for optimized structure.

BHET

C	1.04540200	0.93302400	-0.00000100
C	-0.27549100	1.36997600	-0.00000200
C	-1.32570700	0.44161500	-0.00000100
C	-1.04540200	-0.93302400	0.00000000
C	0.27549100	-1.36997600	0.00000000
C	1.32570700	-0.44161500	0.00000000
H	1.85652100	1.65047200	-0.00000200

H	-0.50536300	2.42906700	-0.00000300
H	-1.85652100	-1.65047200	0.00000000
H	0.50536300	-2.42906700	0.00000100
C	-2.72317200	0.96084000	-0.00000200
C	2.72317200	-0.96084000	0.00000100
O	-3.63925000	-0.02163000	0.00000000
O	3.63925000	0.02163000	0.00000200
O	3.01417400	-2.14713700	0.00000000
O	-3.01417400	2.14713700	-0.00000200
C	-5.02586400	0.38228500	0.00000100
H	-5.22962000	0.98351300	-0.88986900
H	-5.22961900	0.98351500	0.88987100
C	-5.84102000	-0.89856300	0.00000300
H	-5.60175100	-1.49225700	0.89112600
H	-5.60175300	-1.49225900	-0.89112000
O	-7.21287700	-0.49898500	0.00000400
H	-7.76599400	-1.29132600	0.00000500
C	5.02586400	-0.38228500	0.00000200
H	5.22962000	-0.98351300	0.88987300
H	5.22962000	-0.98351500	-0.88986800
C	5.84102000	0.89856300	0.00000100
H	5.60175300	1.49225700	-0.89112300
H	5.60175100	1.49226000	0.89112300
O	7.21287700	0.49898500	0.00000300
H	7.76599400	1.29132600	-0.00000100

H_2O

O	0.00000000	0.00000000	0.11209100
H	0.00000000	0.76677900	-0.47677300
H	0.00000000	-0.76677900	-0.47677300

$HOCH_2CH_2OH$

O	1.78946400	0.25481700	0.00000000
C	0.57282400	-0.49910800	0.00000000
H	0.50655100	-1.13713700	-0.89084100
H	0.50655100	-1.13713700	0.89084100
C	-0.57282400	0.49910800	0.00000000
H	-0.50655100	1.13713700	0.89084100
H	-0.50655100	1.13713700	-0.89084100
O	-1.78946400	-0.25481700	0.00000000
H	-2.53448100	0.36020300	0.00000000
H	2.53448100	-0.36020300	0.00000000

OH^-

O	0.00000000	0.00000000	0.10464300
H	0.00000000	0.00000000	-0.86310100

CF_3COOH

C	-0.59550900	-0.00081900	0.00000100
C	0.94737500	0.15762300	0.00000300
O	1.49221600	1.23309500	0.00000500
O	1.52166000	-1.04005400	-0.00000300
H	2.49167500	-0.94082400	-0.00000500
F	-1.19190900	1.19791800	0.00002200
F	-1.00492400	-0.67964500	-1.09179500
F	-1.00492600	-0.67968400	1.09177100

CF_3COO^{--}

C	-0.52532200	0.01363200	-0.00374200
C	1.04686400	0.00991200	-0.00974200

O	1.59236000	1.13356700	-0.00736000
O	1.53679500	-1.14158000	-0.00184700
F	-1.07938400	1.24318700	-0.13921300
F	-1.03969400	-0.75071800	-1.00690800
F	-1.01171900	-0.50070000	1.16320100

INT-1

C	1.17459700	-0.88314800	-0.08647100
C	-0.15391300	-1.30710500	-0.05765000
C	-1.17119300	-0.45444300	0.37939100
C	-0.83491100	0.84391300	0.79259200
C	0.48662600	1.27721800	0.76776800
C	1.50576600	0.41599900	0.32741900
H	1.95429900	-1.55379600	-0.42866200
H	-0.42492200	-2.30763300	-0.37622500
H	-1.61750900	1.51319400	1.13118900
H	0.74578200	2.28117300	1.08644000
C	-2.62468100	-0.96825800	0.42410300
C	2.90050300	0.91738800	0.31606500
O	-3.36911100	0.12898500	-0.23612600
O	3.78280600	0.00162900	-0.13537500
O	3.24211400	2.03783900	0.67221300
O	-2.84846200	-2.15000600	-0.05893800
C	-4.75436600	-0.12280200	-0.38816000
H	-5.22257200	-0.33222200	0.58379700
H	-4.93053400	-0.98543400	-1.04343800
C	-5.37344400	1.12444300	-0.99686400
H	-4.89235300	1.34581600	-1.95885300
H	-5.22158000	1.98230700	-0.32872200
O	-6.77433900	0.86937400	-1.17984200

H	-7.18539500	1.66126000	-1.55034400
C	5.16647000	0.40299400	-0.18258300
H	5.27331400	1.27907700	-0.82786900
H	5.50848500	0.66135600	0.82336800
C	5.93483800	-0.78371300	-0.73633800
H	5.79651400	-1.65511400	-0.08408900
H	5.56247000	-1.03372800	-1.73769900
O	7.30785800	-0.38835800	-0.78224000
H	7.82890800	-1.12112200	-1.13609200
O	-3.01347900	-0.88003800	1.85749500
H	-3.43135600	-1.74034100	2.00770200

INT-2

C	0.32516700	-0.83503000	0.00000000
C	1.65918500	-1.22921100	0.00000000
C	2.67815200	-0.26626600	0.00000000
C	2.35427700	1.09880500	0.00000000
C	1.01978500	1.49255800	0.00000000
C	0.00102400	0.53007400	0.00000000
H	-0.46244100	-1.57811800	0.00000000
H	1.92335300	-2.28026100	0.00000000
H	3.14096600	1.84304000	0.00000000
H	0.75542600	2.54352800	0.00000000
C	-1.41324500	1.00353900	0.00000000
O	-2.29624300	-0.00826500	0.00000000
O	-1.74207700	2.17968600	0.00000000
C	-3.69540400	0.35049100	0.00000000
H	-3.91817200	0.94496800	0.88985600
H	-3.91817200	0.94496800	-0.88985600
C	-4.46897100	-0.95584600	0.00000000

H	-4.21095300	-1.54156700	-0.89118200
H	-4.21095300	-1.54156700	0.89118200
O	-5.85289100	-0.60020600	0.00000000
H	-6.38023600	-1.40990300	0.00000000
C	4.09009000	-0.73535600	0.00000000
O	4.98589000	0.27302300	0.00000000
H	5.87794800	-0.11525800	0.00000000
O	4.42770400	-1.90862000	0.00000000

INT-3

C	0.44720300	-0.79051700	-0.04491400
C	1.81296600	-1.06606400	-0.03234700
C	2.75617300	-0.02938400	0.00457900
C	2.30168600	1.29724200	0.02882400
C	0.93914200	1.58311400	0.01653800
C	0.00072300	0.54040000	-0.02045800
H	-0.27317200	-1.59952600	-0.07351100
H	2.16785000	-2.09008500	-0.05100700
H	3.03394700	2.09586600	0.05734800
H	0.58805000	2.60911800	0.03529400
C	-1.44315200	0.88803700	-0.03244200
O	-2.24099100	-0.19746500	-0.06847900
O	-1.88294000	2.02945200	-0.01225100
C	-3.66297000	0.04323500	-0.08260800
H	-3.94971200	0.59619300	0.81596800
H	-3.92511000	0.63715100	-0.96236300
C	-4.32510000	-1.32240300	-0.12322100
H	-4.00672400	-1.86427000	-1.02265800
H	-4.03136500	-1.90529900	0.75877900
O	-5.73456100	-1.08518400	-0.13725400

H	-6.19066600	-1.93658200	-0.16314600
C	4.25712200	-0.33808800	0.01826000
O	5.03944800	0.65458500	0.05196500
O	4.58748700	-1.55852100	-0.00527600

INT-4

C	2.90396800	-0.55128800	-0.13806600
C	1.51531900	-0.52344800	-0.21439900
C	0.83157300	0.69712500	-0.12144900
C	1.54491400	1.89335600	0.04917200
C	2.93318400	1.86351900	0.12517800
C	3.61794800	0.64400700	0.03186500
H	3.43428300	-1.49247700	-0.20996800
H	0.95266200	-1.44001600	-0.34618900
H	1.01501500	2.83491000	0.12115300
H	3.49718800	2.77936600	0.25681300
C	-0.64964600	0.68474600	-0.20996300
C	5.10738700	0.66526400	0.11867000
O	-1.19675400	1.89090500	-0.11274700
O	5.64646900	-0.56021900	0.02057200
O	5.77010000	1.68059100	0.26297300
O	-1.29833300	-0.35229000	-0.36137000
C	-2.63963400	1.99524900	-0.18657900
H	-3.08431100	1.41393000	0.62403600
H	-2.97821000	1.60647200	-1.15045500
C	-2.96566900	3.47164900	-0.04873500
H	-2.48689300	4.03673100	-0.85791400
H	-2.59074500	3.84631800	0.91168700
O	-4.38812900	3.56611900	-0.12192900
H	-4.64161400	4.49397400	-0.03001600

C	7.08685500	-0.64445900	0.09212000
H	7.52588000	-0.05616600	-0.71777500
H	7.42693200	-0.24142300	1.04958600
C	7.43370800	-2.11630900	-0.04351000
H	6.96621200	-2.68599600	0.76932200
H	7.05938600	-2.50020300	-1.00076000
O	8.85852300	-2.19786400	0.02284600
H	9.11955200	-3.12391600	-0.06589800
C	-5.84590800	-1.91094300	0.06707300
C	-4.49672900	-1.20388500	0.36275000
O	-4.28239000	-0.66212900	1.42521300
O	-3.69749400	-1.29684900	-0.67624600
H	-2.80555500	-0.85953100	-0.50258000
F	-6.69138700	-1.78774800	1.10052200
F	-6.43839600	-1.38226500	-1.02579800
F	-5.65389300	-3.22875400	-0.16329800

INT-5

C	3.09493100	-0.66900500	-0.27631300
C	1.71307100	-0.80274100	-0.39505000
C	0.86532900	0.25064200	-0.03624500
C	1.41669300	1.44327000	0.45061300
C	2.79659300	1.58020100	0.57251800
C	3.64632600	0.52571300	0.20939200
H	3.74575600	-1.48708200	-0.56056400
H	1.28407000	-1.72266100	-0.77309800
H	0.76519000	2.26532800	0.72292100
H	3.22713600	2.50288700	0.94501400
C	-0.64914600	0.06720100	-0.10360900
C	5.11312500	0.71827800	0.34980800

O	-1.18723900	1.34042400	-0.41515600
O	5.81808900	-0.36470300	-0.02842800
O	5.64074200	1.74166300	0.76183700
O	-0.96904600	-0.90261600	-1.01892600
C	-2.60547100	1.39395600	-0.61250900
H	-3.12821600	0.93798400	0.23430100
H	-2.88405100	0.86143500	-1.52759900
C	-2.96908400	2.86405800	-0.72664700
H	-2.41066700	3.32117300	-1.55373200
H	-2.70663700	3.38742700	0.20164900
O	-4.37937200	2.92672000	-0.96674600
H	-4.63904600	3.85463700	-1.03607000
C	7.25443800	-0.27173700	0.07050700
H	7.61238700	0.54387400	-0.56356900
H	7.53724800	-0.06583800	1.10624000
C	7.79923600	-1.61054000	-0.39414600
H	7.41124600	-2.41279700	0.24590600
H	7.48320000	-1.80322400	-1.42705900
O	9.22246500	-1.51990700	-0.29996800
H	9.60483600	-2.36014700	-0.58546400
C	-5.64277400	-2.21579800	-1.47637100
C	-4.19453700	-1.69811700	-1.77127900
O	-4.01048300	-1.14090300	-2.86517900
O	-3.39844900	-1.91234200	-0.81672800
H	-1.91047900	-1.23425200	-0.90508200
F	-6.48409800	-2.09937000	-2.52903500
F	-5.64712000	-3.52709700	-1.11655100
F	-6.20650600	-1.52458600	-0.44667400
O	-1.14828700	-0.36824200	1.16340000
H	-0.96837000	0.32851800	1.81250300

INT-6

C	1.13806300	-0.72971300	0.08475300
C	-0.25069500	-0.67731100	-0.01171000
C	-0.93454000	-1.45090700	-0.96187500
C	-0.19202900	-2.26957800	-1.82170600
C	1.19681700	-2.31949800	-1.74055200
C	1.87619400	-1.55175000	-0.78197900
H	1.65181200	-0.13156900	0.82837000
H	-0.80894900	-0.03422100	0.65714200
H	-0.72662600	-2.87335900	-2.54659200
H	1.76758000	-2.95501900	-2.40921900
C	-2.47296700	-1.41638200	-1.12370000
C	3.35396200	-1.64151200	-0.71825900
O	-2.93619800	-1.19395600	0.32630000
O	3.88338600	-0.87468200	0.25776800
O	4.04415700	-2.33039200	-1.45864400
O	-2.81822200	-0.18611400	-1.81011300
C	-4.34668200	-1.20645300	0.47381300
H	-4.74639300	-2.22489500	0.37719200
H	-4.81354400	-0.58097700	-0.29624800
C	-4.66693400	-0.65344500	1.85349100
H	-4.30770200	0.38036600	1.93188500
H	-4.16473600	-1.25342600	2.62384600
O	-6.09167500	-0.70979800	2.02250300
H	-6.31304600	-0.34538500	2.88941700
C	5.31788100	-0.89931600	0.39557600
H	5.65077500	-1.92175100	0.59406000
H	5.78197600	-0.55032800	-0.53095000
C	5.64871900	0.02068200	1.55723400

H	5.29330200	1.03615000	1.34190500
H	5.15301600	-0.33764500	2.46818200
O	7.07081900	-0.00714400	1.70057500
H	7.31837500	0.56734100	2.43694500
O	-3.01120000	-2.44473100	-1.67895300
O	-1.71326700	-2.98725200	1.98624000
H	-0.77654200	-2.90190200	1.76619300
H	-2.16364900	-2.34768600	1.37919600
H	-2.41481200	0.58838500	-1.36006200
O	-1.69815700	2.24652600	-0.96911000
C	-1.22345600	2.61783700	0.13215800
C	-0.00225900	3.59115100	-0.02350400
O	-1.54571600	2.32484700	1.29862500
F	0.42387600	4.12737600	1.14524100
F	1.06891900	2.94520100	-0.56594600
F	-0.28372900	4.63706800	-0.84757100

INT-7

C	1.18182700	-0.72145900	0.11621700
C	-0.19829400	-0.61618400	-0.04065700
C	-0.86099700	-1.41650200	-0.98084300
C	-0.13264000	-2.33139100	-1.75508300
C	1.24767500	-2.42246400	-1.61313600
C	1.91251800	-1.61788000	-0.67602700
H	1.69155800	-0.10732300	0.84839900
H	-0.75188700	0.08357100	0.57483200
H	-0.65392100	-2.95756400	-2.47037900
H	1.82148000	-3.11340200	-2.21996300
C	-2.35103900	-1.35449300	-1.15137000
C	3.39100700	-1.74959900	-0.55589000

O	3.91135500	-0.91648900	0.36190500
O	4.06911600	-2.52390300	-1.21455100
O	-2.94466400	-0.17600600	-1.01608900
C	5.34197700	-0.96615300	0.54841700
H	5.63516500	-1.97391800	0.85406100
H	5.84075100	-0.72120600	-0.39302300
C	5.66475300	0.05367800	1.62586500
H	5.34168100	1.05132200	1.30319900
H	5.13563800	-0.20320500	2.55206000
O	7.08087400	0.00586400	1.81146600
H	7.32471100	0.64474500	2.49410600
O	-3.00572400	-2.36403300	-1.39303100
H	-2.32700500	0.64785400	-1.04281000
O	-1.57400900	1.94690600	-1.27469000
C	-1.16554600	2.59781300	-0.26928500
C	-0.24665100	3.80037400	-0.65857600
O	-1.35130500	2.39549900	0.93876200
F	-0.02349100	4.65275300	0.36593300
F	0.97142800	3.34959800	-1.07002400
F	-0.75980900	4.53422300	-1.67742300

TS-1

C	1.20442900	-0.85797500	-0.21394400
C	-0.11986200	-1.27992400	-0.26632100
C	-1.16867100	-0.36686700	-0.08666200
C	-0.86909600	0.99025100	0.10858700
C	0.45332200	1.41691000	0.15479900
C	1.50309200	0.49739000	0.00115000
H	2.00861400	-1.57224600	-0.34413800
H	-0.35751400	-2.32352800	-0.43795100

H	-1.67545000	1.70169100	0.23601000
H	0.68798900	2.46396400	0.31141300
C	-2.57646600	-0.87177000	-0.18148500
C	2.89775000	0.99656900	0.06545900
O	-3.47819300	0.15068300	-0.17513900
O	3.81029000	0.01520800	-0.08581800
O	3.21109100	2.16705600	0.23837000
O	-2.86082100	-1.99116000	-0.61347400
C	-4.85075400	-0.22567700	-0.31796200
H	-5.11924800	-0.94115500	0.46451600
H	-5.01630500	-0.69325400	-1.29389800
C	-5.66206000	1.05153300	-0.19009700
H	-5.34479100	1.77263800	-0.95451900
H	-5.50086400	1.49888800	0.79878900
O	-7.03549600	0.68925900	-0.36920700
H	-7.57942100	1.48076800	-0.26405800
C	5.19747900	0.40463800	-0.03166400
H	5.40986000	1.11801600	-0.83258100
H	5.40759800	0.87980700	0.93030900
C	6.00355800	-0.87062500	-0.20294100
H	5.75810000	-1.57674900	0.60015800
H	5.76189700	-1.33806500	-1.16573500
O	7.37950100	-0.48727500	-0.14832100
H	7.92518000	-1.27755600	-0.25481200
O	-2.58036800	-1.11891000	2.03806300
H	-2.00999400	-1.89883300	2.09718700

TS-2

C	1.09979100	-0.12157300	-0.84887600
C	-0.24077000	-0.29077100	-1.18517000

C	-1.07702100	-1.08861100	-0.39389700
C	-0.54841200	-1.72555400	0.73480600
C	0.79165300	-1.56095900	1.07702900
C	1.62585200	-0.75437700	0.28895500
H	1.73982600	0.50118700	-1.46238100
H	-0.64723600	0.20364200	-2.05901300
H	-1.20316100	-2.34383000	1.33854700
H	1.20286800	-2.05165100	1.95226000
C	-2.54717800	-1.26454300	-0.70880900
C	3.04718300	-0.60161900	0.69043000
O	-2.99506100	0.47016100	-0.22577900
O	3.74021300	0.20065100	-0.14129300
O	3.55408800	-1.13653500	1.66688100
O	-2.75203700	-1.18572700	-2.09696700
C	-4.38608200	0.56230100	-0.09576500
H	-4.76569800	-0.07813200	0.71883100
H	-4.89741100	0.24059000	-1.02082000
C	-4.77838100	2.00815500	0.18914900
H	-4.44303800	2.65131800	-0.63540500
H	-4.29407200	2.34962700	1.11341400
O	-6.20935600	2.06728500	0.32217200
H	-6.46504000	2.98316700	0.49288100
C	5.13243100	0.41196100	0.17039200
H	5.22174400	0.85455700	1.16606000
H	5.65951100	-0.54588200	0.15987000
C	5.66925700	1.34766700	-0.89811000
H	5.55081100	0.88974100	-1.88815500
H	5.11117600	2.29212700	-0.88042500
O	7.04892100	1.55937700	-0.59059300
H	7.42395900	2.15902000	-1.24892200

O	-3.26017400	-2.06724500	-0.06127200
O	-1.75922800	1.15473700	2.01662800
H	-1.03622800	0.51938200	2.10122300
H	-2.22862900	0.88762000	1.16750400
H	-3.63600000	-1.55611300	-2.24269600

TS-3

C	2.93976300	-0.58718200	0.03081700
C	1.54974800	-0.56628600	0.07939900
C	0.85957200	0.65043300	-0.00186500
C	1.57184000	1.84999200	-0.14475300
C	2.96123700	1.82969100	-0.19332400
C	3.65348600	0.61316500	-0.10537700
H	3.47239900	-1.52778600	0.09639200
H	0.98910900	-1.48757500	0.18377200
H	1.03790500	2.78977800	-0.21025800
H	3.52169600	2.75109700	-0.30101000
C	-0.62608600	0.62698300	0.02327200
C	5.14079300	0.64254400	-0.16735900
O	-1.17642000	1.82024300	-0.16711400
O	5.68622000	-0.58409400	-0.09328100
O	5.80525800	1.66242300	-0.27543400
O	-1.26497000	-0.43207400	0.05513100
C	-2.61464600	1.91384100	-0.12031400
H	-2.95793500	1.53612700	0.84553600
H	-3.04671500	1.32112700	-0.93065200
C	-2.95210800	3.38481400	-0.28348700
H	-2.54453400	3.75776900	-1.23156800
H	-2.51144800	3.96146300	0.53896600
O	-4.37831300	3.46869600	-0.26714100

H	-4.63396400	4.39699800	-0.34683600
C	7.12693600	-0.65847100	-0.14758700
H	7.47890500	-0.22543000	-1.08758300
H	7.55288600	-0.09310600	0.68541800
C	7.48055700	-2.13196100	-0.05250600
H	7.09926100	-2.54658900	0.88901000
H	7.02390400	-2.67922900	-0.88665700
O	8.90657600	-2.20519000	-0.10757500
H	9.17094600	-3.13243400	-0.04535400
C	-5.82644700	-1.84599800	-0.05911700
C	-4.45331700	-1.25859400	-0.48244400
O	-3.63111200	-1.24454700	0.53784600
O	-4.25253200	-0.87808000	-1.61676900
H	-2.71842700	-0.85501500	0.29424600
F	-5.68907200	-3.10113200	0.42330800
F	-6.39087400	-1.09113300	0.91023100
F	-6.67588500	-1.89085800	-1.09674300
O	-0.71460800	0.89988500	2.75228100
H	-0.84097700	-0.04299900	2.58102500

TS-4

C	1.51850500	-0.55822900	-0.23599300
C	0.14980800	-0.51370900	-0.48791700
C	-0.50030100	-1.60282700	-1.08593400
C	0.25013200	-2.73401600	-1.43074800
C	1.62031200	-2.78456600	-1.18314700
C	2.26685700	-1.69570600	-0.58104300
H	2.00922200	0.28538100	0.23532700
H	-0.41215100	0.36275500	-0.18777600
H	-0.26154800	-3.57329700	-1.88793300

H	2.19834900	-3.66267500	-1.44982500
C	-2.00418000	-1.61313800	-1.36542400
C	3.72499800	-1.79011800	-0.32545500
O	-2.48204900	-1.39707100	0.38123700
O	4.22628500	-0.68103100	0.25497400
O	4.41915100	-2.75967400	-0.60119900
O	-2.48224100	-0.41376200	-1.89960600
C	-3.86178300	-1.59834700	0.52912000
H	-4.12841300	-2.67000800	0.52284900
H	-4.41826600	-1.12067600	-0.29275400
C	-4.31720100	-0.98715600	1.84914400
H	-4.10304100	0.08973400	1.84743300
H	-3.76837000	-1.44716300	2.68204900
O	-5.72957100	-1.22051100	1.99219200
H	-6.02307900	-0.83206400	2.82660400
C	5.63787900	-0.68394000	0.54830200
H	5.86905000	-1.50730200	1.22957900
H	6.20483100	-0.82036300	-0.37655000
C	5.94163200	0.65976700	1.18665200
H	5.68521400	1.46900800	0.49148400
H	5.34588900	0.78132700	2.09997700
O	7.34047300	0.65836600	1.48119900
H	7.56986500	1.50099000	1.89472100
O	-2.52166500	-2.65357100	-1.84332000
O	-1.10697000	-3.17111900	1.77179500
H	-0.35475200	-3.38237500	1.20351500
H	-1.63139500	-2.49076600	1.24510000
H	-2.24570800	0.37121000	-1.35340200
O	-2.21161500	2.10173400	-0.87075100
C	-1.71416900	2.72142200	0.10254600

C	-1.91068100	4.27433400	-0.00302900
O	-1.09597700	2.30885500	1.10014000
F	-1.46848300	4.95680400	1.07927100
F	-1.24414500	4.78043200	-1.07922700
F	-3.22014800	4.60877000	-0.16363100

a1

O	3.42192100	-2.43831600	0.20506800
C	3.00724600	-1.42234900	-0.31575000
C	1.56193000	-1.08423900	-0.46311800
C	0.62601100	-1.99152600	0.04823600
H	0.98738100	-2.89492700	0.52548600
C	-0.73213300	-1.71987800	-0.04729700
H	-4.99879500	-0.18962900	0.39270700
H	-1.46303700	-2.40480600	0.36401400
O	-3.41426400	-1.20560700	-0.50255800
C	-4.81861100	-0.89225100	-0.42316100
O	3.81097700	-0.46760100	-0.82860800
C	1.13191400	0.09527000	-1.08315500
H	1.86209700	0.79097900	-1.47634900
O	-3.02258300	0.97090400	-0.88433700
C	-2.59910000	-0.16803000	-0.69651800
C	-1.16246700	-0.53283200	-0.65551900
C	-0.22679000	0.36584600	-1.18436100
H	-0.57083900	1.27832000	-1.65514300
H	-5.31582200	-1.84229600	-0.23549700
C	5.21857200	-0.72849900	-0.70911000
H	5.71499400	0.12950600	-1.16042900
H	5.49987500	-0.82925800	0.34153500
H	5.48241800	-1.64896900	-1.23495700

H	-5.16207200	-0.45217600	-1.36117000
C	0.11576000	2.67523700	2.15630800
C	-1.15058100	2.05985300	1.52784900
O	-1.68792400	1.08068600	1.98925000
O	-1.48206400	2.71576500	0.42498200
H	-2.16869400	2.18433400	-0.06461700
F	1.18172600	2.37392500	1.38407000
F	0.02673200	4.01291800	2.24365100
F	0.32623000	2.17964400	3.37850400

a2

O	3.78690000	-2.27461100	-0.80863300
C	3.40302000	-1.12732000	-0.70073000
C	1.97794200	-0.70387500	-0.82603400
C	1.02398100	-1.69849100	-1.07457900
H	1.35785700	-2.72588400	-1.16138000
C	-0.31766100	-1.36272900	-1.19827000
H	-4.62606000	-0.10443800	-0.47339500
H	-1.06435600	-2.12568200	-1.37916600
O	-2.93949800	-0.60515900	-1.57546300
C	-4.35178100	-0.32397000	-1.50682600
O	4.22628400	-0.08738000	-0.45295700
C	1.58304500	0.63412200	-0.70203600
H	2.32710600	1.39675600	-0.50943500
O	-2.55623200	1.50192300	-0.90198400
C	-2.13766000	0.37604400	-1.16931100
C	-0.71332300	-0.02381500	-1.07420300
C	0.24104600	0.97146200	-0.82689500
H	-0.08046200	2.00213900	-0.73620200
H	-4.84313800	-1.23023500	-1.85642800

C	5.61524500	-0.42917000	-0.31786700
H	6.13038800	0.51025500	-0.12189000
H	5.75759300	-1.12800500	0.50950900
H	5.98627800	-0.88944500	-1.23644400
H	-4.60241900	0.52627000	-2.14333600
C	-1.70190400	0.98923900	3.68455400
C	-2.12120000	0.91329000	2.20112800
O	-2.56729600	-0.09811000	1.70807700
O	-1.91051100	2.07501200	1.60356700
H	-2.16936600	1.98795900	0.64409200
F	-1.92641000	-0.17633500	4.29666900
F	-0.39139300	1.28061800	3.78409100
F	-2.39602400	1.94955800	4.32177800

a3

O	4.97991200	-0.47979400	-2.43013800
C	4.49008000	-0.01780000	-1.41924000
C	3.02681100	-0.00079300	-1.12815600
C	2.16799700	-0.55308000	-2.08631700
H	2.59851100	-0.96635900	-2.99101600
C	0.79623500	-0.56483700	-1.86927800
H	-3.72742800	0.41870200	-1.06022500
H	0.12480200	-0.99057300	-2.60458100
O	-1.90934900	-0.55665200	-1.38117900
C	-3.33911000	-0.59548200	-1.18284300
O	5.21587100	0.54121300	-0.42875500
C	2.50617500	0.54183700	0.05375900
H	3.17853300	0.96614500	0.78888100
O	-1.65722300	0.46239700	0.62055000
C	-1.18237700	-0.01079200	-0.40699600

C	0.27547700	-0.02142100	-0.68652100
C	1.13420700	0.53077000	0.27267700
H	0.70820100	0.94407600	1.17932200
H	-3.58508100	-1.19228600	-0.30317500
C	6.63594500	0.55576600	-0.64817800
H	7.06336800	1.03552900	0.23123800
H	7.01578800	-0.46268900	-0.75614800
H	6.87655200	1.11989200	-1.55207800
H	-3.74053000	-1.04711400	-2.08815100
C	-5.68467400	0.05367400	3.31842500
C	-4.56199500	-0.25600700	2.30431900
O	-4.41493800	-1.35855100	1.82677300
O	-3.84389900	0.82735200	2.07243100
H	-3.07819300	0.61785700	1.46999300
F	-6.46910000	-1.01353400	3.49209100
F	-5.15664600	0.39579100	4.50795400
F	-6.44391600	1.07674800	2.88455000

a4

O	4.11488300	-2.78069800	0.28316300
C	3.58645600	-1.68780900	0.23226100
C	2.11084400	-1.46986200	0.19640100
C	1.28662100	-2.60231300	0.22337900
H	1.75055900	-3.58073800	0.26889700
C	-0.09332700	-2.45879100	0.19229000
H	-4.59882800	-1.41636300	0.98002400
H	-0.73762900	-3.32885000	0.21279500
O	-2.80988900	-2.13756700	0.18253500
C	-4.24431600	-2.02362500	0.14475200
O	4.27622300	-0.52905700	0.20197100

C	1.54507800	-0.19062200	0.13850200
H	2.18693200	0.68121300	0.11938900
O	-2.70529800	0.08974600	-0.00507400
C	-2.12930200	-0.98729000	0.09404700
C	-0.65952400	-1.17549200	0.13210300
C	0.16282900	-0.04133800	0.10599800
H	-0.27286900	0.95035200	0.06364900
H	-4.61729600	-3.04349100	0.22231100
C	5.70545900	-0.66959000	0.23493800
H	6.10025000	0.34511500	0.20782500
H	6.01777300	-1.18199800	1.14774000
H	6.05274800	-1.24289200	-0.62765900
H	-4.56437000	-1.56356500	-0.79234000
C	-2.21754100	4.92752000	-0.65918000
C	-1.86243800	3.45135900	-0.37834600
O	-0.73389700	3.10284500	-0.11367000
O	-2.93900900	2.69553300	-0.47727000
H	-2.71791200	1.74520000	-0.29175500
F	-1.13614700	5.70230900	-0.53951400
F	-2.70461000	5.06129700	-1.90671300
F	-3.15244600	5.35946300	0.20655500

b5

O	3.67748200	-1.92799500	-0.40679600
C	3.37408800	-0.75337300	-0.34629900
C	2.00697100	-0.22368200	-0.61985000
C	1.00778800	-1.14561800	-0.95214600
H	1.26709100	-2.19666300	-1.00229400
C	-0.28606600	-0.70917100	-1.20876800
H	-4.51624700	1.38495500	-0.69004700

H	-1.05722500	-1.42266200	-1.47226400
O	-2.91477400	0.21205300	-1.32426400
C	-4.26592900	0.69300300	-1.49633200
O	4.24600400	0.22411700	-0.02121500
C	1.70806900	1.14367000	-0.55449800
H	2.48738700	1.84929400	-0.29526000
O	-2.22338900	2.34659400	-1.60258900
C	-1.94977600	1.19131700	-1.38064200
C	-0.58634600	0.65790600	-1.13647000
C	0.41669600	1.58179100	-0.81747200
H	0.15920900	2.63326100	-0.76947100
H	-4.89756700	-0.19370900	-1.46239000
C	5.58298500	-0.22104900	0.25819500
H	6.14843200	0.67948200	0.49413600
H	5.58623300	-0.91200900	1.10427500
H	6.00692900	-0.72717000	-0.61208000
H	-4.36146400	1.20277600	-2.45544400
C	-1.80969700	-0.87588900	3.21792600
C	-2.22364900	-0.25196800	1.86982400
O	-2.18380300	0.93607300	1.65801700
O	-2.59200900	-1.20024300	1.01401100
H	-2.76660000	-0.76356800	0.14505700
F	-1.55336700	0.07822900	4.11534300
F	-2.78553400	-1.66764100	3.69637700
F	-0.70192300	-1.62212200	3.05343300

b6

O	3.46154900	-1.51163700	0.35998100
C	2.94060000	-0.54264600	0.87383300
C	1.46741300	-0.33100600	0.96490600

C	0.63735100	-1.29884900	0.38807300
H	1.09191000	-2.16056000	-0.08596400
C	-0.74175900	-1.13114400	0.40372300
H	-5.25457100	-0.59174600	0.84062600
H	-1.38458900	-1.86351200	-0.06784600
O	-3.38478100	-0.37216500	-0.05945800
C	-4.81917900	-0.21803600	-0.08773100
O	3.63896100	0.47171100	1.42696800
C	0.91051100	0.79510100	1.58466900
H	1.56194600	1.53603600	2.03080700
O	-3.35341200	1.00151900	1.73840600
C	-2.75754400	0.27820900	0.97247800
C	-1.29684700	0.01106000	0.99535700
C	-0.46878300	0.96018200	1.60722400
H	-0.92158200	1.82862000	2.07178800
H	-5.15875300	-0.80820300	-0.93776800
C	5.06702900	0.33186700	1.36080900
H	5.47153700	1.22257300	1.83982900
H	5.38760100	-0.56991900	1.88751400
H	5.39613900	0.26856800	0.32115600
H	-5.08693900	0.83345100	-0.20883300
C	0.56852400	0.48263800	-2.75601500
C	-0.79255000	-0.19793300	-2.49989100
O	-0.97862700	-1.37342200	-2.68746200
O	-1.68363300	0.68789000	-2.05507700
H	-2.45650700	0.19396000	-1.70924800
F	1.53550800	-0.43743400	-2.80161000
F	0.54237700	1.14322600	-3.92881800
F	0.86110100	1.36161000	-1.78241600

b7

O	4.04040600	-0.85487900	-1.30448200
C	3.80665000	-0.17583600	-0.32511300
C	2.45723100	0.35486500	0.02575300
C	1.39303300	0.03801400	-0.82596500
H	1.59098800	-0.57082200	-1.70035400
C	0.11319100	0.50130900	-0.54671000
H	-4.00980400	1.72732800	1.57463900
H	-0.70472900	0.25953400	-1.21305000
O	-2.46593900	1.22523300	0.25802000
C	-3.78890200	1.76520900	0.50704500
O	4.74751900	0.18386600	0.57297000
C	2.23732500	1.14834200	1.15948300
H	3.06739200	1.39176500	1.81088000
O	-1.65135700	2.69906500	1.76240000
C	-1.44782700	1.82637900	0.94918200
C	-0.10956300	1.28729700	0.59245600
C	0.95959200	1.61648200	1.43686400
H	0.76562500	2.23763800	2.30363000
H	-4.46328500	1.12940400	-0.06216100
C	6.07185600	-0.29787200	0.29211400
H	6.69664000	0.07232100	1.10383500
H	6.08249900	-1.38972000	0.26267600
H	6.42102000	0.08431700	-0.66979200
H	-3.82725200	2.80105500	0.16675000
C	-3.67134600	-3.23646300	-1.35528200
C	-3.67764200	-1.77119800	-0.87062200
O	-4.64108100	-1.05141900	-0.98413800
O	-2.50033500	-1.45806900	-0.34394000
H	-2.50512300	-0.50771600	-0.07034500

F	-4.86683400	-3.57157100	-1.84413200
F	-2.74892500	-3.39784200	-2.32156700
F	-3.37102700	-4.06601200	-0.34104100

b8

O	2.23701000	-2.98756400	0.10605900
C	1.88102700	-1.84978100	-0.10064000
C	0.47473100	-1.39241100	-0.22210800
C	-0.52770100	-2.28098900	0.19100200
H	-0.23825700	-3.25191500	0.57634300
C	-1.86324400	-1.91052600	0.10636100
H	-6.06791800	0.14057700	0.43701400
H	-2.64490400	-2.58680400	0.42920000
O	-4.49413500	-1.12956800	-0.07749500
C	-5.87828200	-0.75371200	-0.16090400
O	2.78266300	-0.82386500	-0.23939200
C	0.13481800	-0.13495900	-0.74124200
H	0.90132600	0.54941200	-1.08475600
O	-3.96178000	0.88431500	-0.95157900
C	-3.62186200	-0.19703500	-0.51597400
C	-2.20491000	-0.64977900	-0.40189300
C	-1.20371700	0.23067700	-0.82738400
H	-1.49302000	1.19658000	-1.22480300
H	-6.43691500	-1.60372100	0.22871900
C	4.17228300	-1.21625500	-0.19400000
H	4.73743400	-0.29083800	-0.29339200
H	4.39510800	-1.71122300	0.75247800
H	4.39184800	-1.89577000	-1.01860400
H	-6.15694100	-0.55025100	-1.19736100
C	2.66300000	3.90536800	1.11385500

C	2.44034100	2.58803300	0.34118500
O	2.05496800	2.56043500	-0.80306100
O	2.72956900	1.54823000	1.11419200
H	2.60025100	0.71401200	0.60061900
F	2.39447500	4.95436000	0.33447100
F	1.86280200	3.95281900	2.19358500
F	3.93992800	3.99213900	1.52950100

c9

O	4.01719400	-2.80038900	0.09934800
C	3.58838600	-1.74877700	0.53145700
C	2.15777200	-1.33523500	0.47268500
C	1.25052900	-2.21637700	-0.12861400
H	1.62354400	-3.15489800	-0.52212700
C	-0.09479300	-1.88088700	-0.21477200
H	-4.57460100	-0.56160800	0.38540900
H	-0.80316800	-2.55382700	-0.68198000
O	-2.73454600	-1.12136200	-0.42986100
C	-4.11615900	-0.74306800	-0.58884300
O	4.36891200	-0.81824600	1.12319100
C	1.71072600	-0.11229200	0.99031600
H	2.41992700	0.56466700	1.45025200
O	-2.39864000	0.78976900	0.72226900
C	-1.96838800	-0.24282100	0.23548600
C	-0.54176100	-0.65921800	0.30571700
C	0.36491300	0.22220800	0.90729000
H	-0.00585200	1.16457600	1.29193400
H	-4.59014200	-1.58712900	-1.08792400
C	5.76123200	-1.15879300	1.20700300
H	6.24067700	-0.31472900	1.70128000

H	5.89714400	-2.07437000	1.78731000
H	6.17890000	-1.30995100	0.20890100
H	-4.19038200	0.16172900	-1.19332500
C	-1.82473400	2.59703100	-1.67868800
C	-3.03547400	2.98649100	-0.80689500
O	-4.16214400	2.66065300	-1.08098600
O	-2.66634300	3.78845800	0.19392000
H	-3.47147500	3.99923100	0.69680700
F	-2.02446300	1.39133900	-2.23206600
F	-1.70076300	3.49903200	-2.67202800
F	-0.67604000	2.56879100	-0.99246000

c10

O	4.12800000	-1.86205900	-1.22465900
C	3.65623400	-0.80617200	-0.85315000
C	2.19934000	-0.48883500	-0.85549900
C	1.31743000	-1.47873200	-1.30604900
H	1.72777700	-2.42807600	-1.63046900
C	-0.05169800	-1.23968300	-1.32511900
H	-4.41793800	-0.41171900	-0.10428500
H	-0.74273700	-2.00346900	-1.65922800
O	-2.75787100	-0.70703000	-1.32143500
C	-4.17828800	-0.53186200	-1.16161100
O	4.40296900	0.21789000	-0.38705300
C	1.70383700	0.75045000	-0.42689600
H	2.39320500	1.51267900	-0.08495700
O	-2.46718300	1.33536600	-0.40637200
C	-2.00603800	0.29940000	-0.85245600
C	-0.54726100	-0.00194400	-0.89691100
C	0.33485400	0.99253100	-0.45188900

H	-0.07680700	1.94072700	-0.12594300
H	-4.62902800	-1.43811700	-1.56320400
C	5.81902400	-0.02340500	-0.36426500
H	6.26544800	0.88796900	0.03132800
H	6.05109000	-0.87729000	0.27632300
H	6.18770200	-0.22850300	-1.37186500
H	-4.51793100	0.34749900	-1.71287300
C	-3.28690700	1.19926000	2.66575400
C	-2.11572600	0.28745200	2.24980300
O	-2.26425000	-0.84019800	1.85181200
O	-0.94729700	0.90495400	2.44722300
H	-0.24691100	0.30660400	2.13213700
F	-4.43265300	0.74095100	2.14648100
F	-3.39644500	1.18079100	4.01064300
F	-3.10394400	2.46257500	2.27469000

c11

O	4.11944900	-0.85878600	-0.96373100
C	3.59731100	0.20383500	-0.69141500
C	2.12421700	0.43533300	-0.67771100
C	1.29345200	-0.63445400	-1.04030600
H	1.75246900	-1.57454700	-1.32502700
C	-0.08853900	-0.47769300	-1.03897800
H	-4.57517700	0.43422200	0.17962600
H	-0.73559400	-1.30344500	-1.30731900
O	-2.81194300	-0.18487900	-0.74461100
C	-4.24557300	-0.05156700	-0.73910800
O	4.29359700	1.31218900	-0.36327700
C	1.56324900	1.66757200	-0.31986100
H	2.21195900	2.49009300	-0.04518600

O	-2.65051800	2.04650000	-0.42033200
C	-2.12343400	0.96751800	-0.59437200
C	-0.64870200	0.75170100	-0.65937700
C	0.18121300	1.82272900	-0.31254400
H	-0.27825900	2.76338500	-0.03167600
H	-4.62830900	-1.06845200	-0.79531500
C	5.72179000	1.15449400	-0.35736000
H	6.12315000	2.12991000	-0.08579700
H	6.01953700	0.39749200	0.37180100
H	6.07636300	0.85151700	-1.34507300
H	-4.57064700	0.53867200	-1.59848300
C	-3.07965300	-1.68804100	2.20672700
C	-1.75718600	-1.98445500	1.47186000
O	-1.63758900	-2.85864700	0.65411300
O	-0.80097600	-1.15742000	1.90433400
H	-0.00458200	-1.32037700	1.36660700
F	-3.02835300	-2.17651700	3.45804600
F	-3.31025500	-0.36472200	2.27889400
F	-4.10245600	-2.26314500	1.56485300

c12

O	3.51352900	-1.84419700	1.33321800
C	3.12828200	-0.70073200	1.19272200
C	1.69204500	-0.30362100	1.12598400
C	0.73014900	-1.31741100	1.25012700
H	1.06892500	-2.33685000	1.39650400
C	-0.62485100	-1.00684100	1.18245600
H	-5.04353600	0.75832700	1.31888800
H	-1.37785600	-1.78061600	1.26510500
O	-3.28698800	-0.33561800	1.03746100

C	-4.66924400	-0.08463700	0.73445500
O	3.95725000	0.35648800	1.07759800
C	1.28928300	1.02375800	0.93847100
H	2.03822700	1.79935000	0.83828000
O	-2.83309000	1.81623600	0.51350000
C	-2.46053800	0.70189300	0.82035700
C	-1.02557700	0.32398600	0.98600500
C	-0.06526800	1.33312800	0.86578200
H	-0.40275500	2.34943700	0.69839200
H	-5.19691500	-1.00130700	0.99424300
C	5.35841300	0.04046000	1.12764500
H	5.87713300	0.99134800	1.01430000
H	5.61007900	-0.42613300	2.08270500
H	5.62387200	-0.64408900	0.31900000
H	-4.77873700	0.13482000	-0.32979400
C	-1.18091000	0.44833200	-3.78585200
C	-1.21490700	-0.28512300	-2.42901500
O	-2.23739100	-0.59263600	-1.86985500
O	0.02781900	-0.50608700	-2.00034200
H	-0.02261100	-0.92043700	-1.11639100
F	-2.42141600	0.68233500	-4.21693500
F	-0.53599500	1.62113800	-3.66176300
F	-0.53641800	-0.29469500	-4.70324700

c13

O	3.37318200	-2.23135200	0.42221000
C	2.97965300	-1.20003000	0.92816200
C	1.54607500	-0.79351900	0.99717800
C	0.59795700	-1.64494800	0.41741600
H	0.94373600	-2.55829100	-0.05284400

C	-0.75102200	-1.30921000	0.43683000
H	-5.20461200	0.13691500	1.22761500
H	-1.48945600	-1.95072100	-0.02719200
O	-3.39781100	-0.56264800	0.44830000
C	-4.75574100	-0.12862400	0.26822500
O	3.79710600	-0.28742000	1.49319700
C	1.13594600	0.39883500	1.60936100
H	1.87518200	1.05189000	2.05650200
O	-2.96123500	1.39681400	1.48774700
C	-2.58657200	0.33633500	1.03034800
C	-1.16278500	-0.11532300	1.04271900
C	-0.21535600	0.73443100	1.63286600
H	-0.56252300	1.65135800	2.09600400
H	-5.27547000	-0.97291900	-0.18254600
C	5.19555800	-0.61681700	1.45488600
H	5.70536200	0.21271500	1.94289100
H	5.38187900	-1.55240400	1.98698000
H	5.53335500	-0.72462500	0.42176100
H	-4.78067000	0.73830700	-0.39543000
C	-0.62317000	1.27376300	-3.77586000
C	-0.91772400	1.20740200	-2.26249200
O	-2.02523800	1.04436000	-1.81566300
O	0.21556300	1.34027800	-1.57461600
H	0.01472100	1.25464500	-0.62149500
F	-1.75974800	1.21425700	-4.47275800
F	0.01441300	2.41589100	-4.08397000
F	0.15945000	0.24065000	-4.13632000

c14

O	4.19550300	-0.49844000	-1.03285600

C	3.69172500	0.43326900	-0.43701100
C	2.22419700	0.66115300	-0.31683700
C	1.36968100	-0.26558500	-0.92631400
H	1.80815600	-1.10379500	-1.45537900
C	-0.00722800	-0.10448000	-0.84452000
H	-4.50974700	0.61908600	0.45061300
H	-0.67145000	-0.82319200	-1.30451700
O	-2.74474200	0.23249000	-0.59315800
C	-4.16943600	0.45471500	-0.57183900
O	4.41330600	1.38451700	0.19517800
C	1.69091500	1.75542700	0.37675600
H	2.35828000	2.46756800	0.84604800
O	-2.50359800	2.19183700	0.50325300
C	-2.00744800	1.22050100	-0.02949600
C	-0.54112500	0.98829900	-0.15013300
C	0.31317800	1.91694400	0.45678100
H	-0.12527600	2.75491800	0.98619400
H	-4.60936300	-0.45188800	-0.98074500
C	5.83730600	1.21911800	0.11650500
H	6.26101500	2.05290100	0.67499000
H	6.13695500	0.26607100	0.55848200
H	6.16866300	1.24363200	-0.92427000
H	-4.41878300	1.32670600	-1.18026100
C	-2.53145000	-2.10693700	1.55084600
C	-2.79633700	-2.66187700	0.13646400
O	-3.89782400	-2.75812600	-0.33865800
O	-1.65773100	-3.07856300	-0.43263800
H	-1.89760200	-3.46813100	-1.29132900
F	-3.53316000	-1.30440800	1.92412900
F	-2.46563000	-3.13859000	2.41348900

F	-1.38095500	-1.42512900	1.62023500

c15

O	3.91876500	-1.58186400	1.23118500
C	3.46561100	-0.47636000	1.45212400
C	2.04901800	-0.08179400	1.21187100
C	1.18268400	-1.04877900	0.68817400
H	1.57377800	-2.03700700	0.47618800
C	-0.14899900	-0.73657600	0.44440200
H	-4.64170800	0.43053900	0.72985600
H	-0.81529900	-1.48072700	0.03047700
O	-2.77820500	-0.02737400	-0.08512600
C	-4.17139300	0.29702600	-0.24674300
O	4.20497600	0.53989700	1.94997800
C	1.57482400	1.20681600	1.49230200
H	2.25138700	1.94942700	1.89665500
O	-2.50127500	2.03373800	0.79174400
C	-2.03625400	0.95312900	0.49228500
C	-0.62225800	0.55185400	0.72333500
C	0.24355300	1.51943500	1.24932600
H	-0.14911800	2.50781700	1.45824200
H	-4.60684200	-0.54749300	-0.77694900
C	5.58151000	0.22107000	2.20278200
H	6.02835800	1.13555200	2.59113700
H	5.65962500	-0.58721300	2.93371800
H	6.07819900	-0.09009000	1.28081900
H	-4.28399200	1.21182100	-0.82868900
C	-1.75454000	0.10606900	-3.09986900
C	-2.11503900	-1.33475700	-2.68484900
O	-1.30321400	-2.12829400	-2.28744700

O	-3.41346600	-1.58653300	-2.90542000
H	-3.55693800	-2.52299400	-2.68355100
F	-0.68189000	0.52470200	-2.42617100
F	-1.46833200	0.11423100	-4.41549900
F	-2.76352100	0.96472500	-2.88629500

d16

O	4.05238100	-0.77709400	1.45803800
C	3.67418400	0.05958300	0.66192800
C	2.25831200	0.49491100	0.50457300
C	1.29954700	-0.09389300	1.33828900
H	1.62486500	-0.83666600	2.05756600
C	-0.03623900	0.27361700	1.23534400
H	-4.19747800	2.46005100	1.02965100
H	-0.78490400	-0.18235200	1.86969100
O	-2.66915400	1.04063200	0.99338800
C	-4.05613400	1.39003600	0.86344000
O	4.50726900	0.70261200	-0.18627500
C	1.87194700	1.45715500	-0.43800000
H	2.61938700	1.90753600	-1.07937400
O	-2.21814700	2.48630300	-0.68187300
C	-1.83920000	1.66624300	0.13008400
C	-0.42234300	1.23494600	0.29234900
C	0.53576500	1.82365000	-0.54166300
H	0.20901400	2.56403000	-1.26275500
H	-4.57626500	0.80710200	1.62194900
C	5.88863600	0.32694300	-0.08166800
H	6.41444400	0.93270800	-0.81880300
H	6.26558200	0.52893700	0.92364800
H	6.01393900	-0.73694900	-0.29649500

H	-4.41902000	1.13083200	-0.13273500
C	-2.86324000	-1.84980400	-0.61050100
C	-2.97712700	-3.11024300	-1.49392500
O	-3.99090900	-3.74209600	-1.62990300
O	-1.79493300	-3.37285900	-2.06534600
H	-1.90941100	-4.16949400	-2.61161000
F	-4.03904600	-1.58563000	-0.03540400
F	-1.94111500	-2.03656200	0.34742400
F	-2.50059600	-0.79419600	-1.36103000

d17

O	3.94290800	-0.38576300	2.13917600
C	3.53529100	0.51410000	1.43164100
C	2.10395000	0.72525800	1.07646900
C	1.16600100	-0.18070600	1.58677100
H	1.51755400	-0.99279100	2.21268600
C	-0.18264300	-0.03213800	1.28841800
H	-4.61936400	1.39830700	0.68537100
H	-0.91285600	-0.73273500	1.67216100
O	-2.84281200	0.30571100	0.66500900
C	-4.23374700	0.43883400	0.33394500
O	4.34526300	1.44262700	0.87605500
C	1.68391600	1.78532900	0.26199300
H	2.41565300	2.48113800	-0.12947700
O	-2.44031900	2.14568200	-0.58110300
C	-2.03364900	1.23986300	0.11854800
C	-0.60265800	1.02810900	0.47466200
C	0.33494300	1.93417000	-0.03561800
H	-0.01734100	2.74512000	-0.66266900
H	-4.73367200	-0.38768700	0.83651300

C	5.73971800	1.29228600	1.18152100
H	6.24347500	2.10742200	0.66327100
H	5.90539900	1.35948600	2.25932200
H	6.10705000	0.32553700	0.82928700
H	-4.37320000	0.37084100	-0.74634600
C	-2.21080900	-2.08127900	-1.53676400
C	-2.02564500	-3.09122400	-2.68899500
O	-1.11954400	-3.87862300	-2.74958100
O	-3.01409100	-2.94831000	-3.58228500
H	-2.85848200	-3.59815400	-4.28914400
F	-1.23057300	-2.21510900	-0.64581400
F	-2.19852600	-0.82545500	-2.02015100
F	-3.39118400	-2.28984600	-0.92487500

d18

O	4.12354400	-0.29809500	-1.63060100
C	3.60165800	0.62447700	-1.03645200
C	2.13200300	0.78630500	-0.85406400
C	1.29610800	-0.19467200	-1.40174300
H	1.74938300	-1.02576700	-1.92964500
C	-0.08266300	-0.09451300	-1.26344300
H	-4.49132900	0.21179500	0.26023000
H	-0.73499400	-0.85271700	-1.67609800
O	-2.80875600	0.15583900	-0.96717400
C	-4.23231900	0.24094100	-0.79961900
O	4.30457900	1.62674500	-0.46332000
C	1.57898200	1.87256300	-0.16279800
H	2.23220400	2.62670400	0.25842500
O	-2.62432200	2.07243100	0.21261700
C	-2.10472900	1.15311700	-0.38765700

C	-0.63528400	0.99161900	-0.57244700
C	0.20012500	1.97237400	-0.02418600
H	-0.25405600	2.80159000	0.50595500
H	-4.63913000	-0.62749700	-1.31548700
C	5.72940800	1.52687600	-0.60573900
H	6.13736000	2.39965000	-0.09716700
H	6.09676600	0.60601100	-0.14672600
H	6.01011400	1.52916200	-1.66157900
H	-4.61328400	1.16658700	-1.23636700
C	-2.24842600	-2.16505700	1.32821100
C	-2.02189500	-3.14352300	2.50016900
O	-1.22502400	-4.04309200	2.47581000
O	-2.83964900	-2.83454300	3.51540200
H	-2.66561000	-3.47234800	4.22889700
F	-1.41763800	-2.44886300	0.32653300
F	-3.51648200	-2.26302300	0.88564900
F	-2.03663600	-0.90106300	1.73442500

d19

O	4.01656100	-1.55804500	0.75267400
C	3.67500900	-0.39214700	0.72635400
C	2.26235100	0.07432500	0.80359600
C	1.26060200	-0.89794000	0.91565000
H	1.55219500	-1.94167700	0.93938900
C	-0.07430400	-0.52097700	0.99231400
H	-4.24722500	1.34610300	2.11877300
H	-0.85441700	-1.26679100	1.06714300
O	-2.70088000	0.28828400	1.20356000
C	-4.08184000	0.67627800	1.27208200
O	4.55050200	0.63248100	0.62186000

C	1.91985100	1.43247200	0.76603300
H	2.70007300	2.17847300	0.67876700
O	-2.17210700	2.46734000	0.93875600
C	-1.82914700	1.30506700	1.02526800
C	-0.41621100	0.83726100	0.95471700
C	0.58450700	1.80950100	0.83991100
H	0.29159200	2.85268600	0.80955200
H	-4.63887900	-0.25104200	1.39690500
C	5.93040500	0.24517300	0.54305700
H	6.49221800	1.17483000	0.46003800
H	6.22572400	-0.30533600	1.43937100
H	6.10117000	-0.38857100	-0.33032100
H	-4.38142400	1.18141000	0.35233800
C	-2.74319700	-1.15467800	-1.75023900
C	-2.69869700	-1.79340100	-3.15474100
O	-2.40810700	-2.94202500	-3.35773700
O	-3.03334600	-0.88371500	-4.07922100
H	-2.99524200	-1.32585200	-4.94480400
F	-2.36923200	-2.04503100	-0.83105300
F	-1.92346800	-0.09214600	-1.69745900
F	-3.99504500	-0.74232300	-1.47252800

d20

O	3.89303500	-1.75967900	0.93518500
C	3.54415100	-0.61035800	1.11960600
C	2.12875100	-0.14610600	1.10299500
C	1.13308700	-1.10045000	0.85961100
H	1.43135600	-2.12912300	0.69274000
C	-0.20413700	-0.72482100	0.83340600
H	-4.51509100	0.92625900	1.75957600

H	-0.97793400	-1.45422600	0.63435900
O	-2.84443500	0.06688100	0.85505600
C	-4.22628000	0.45591500	0.81709600
O	4.41309300	0.39574400	1.36449100
C	1.77812900	1.19321900	1.31875900
H	2.55374800	1.92553800	1.50585000
O	-2.31086600	2.24065700	1.15171600
C	-1.96893700	1.08161800	1.02642200
C	-0.55416800	0.61459000	1.04923800
C	0.44085400	1.56953400	1.28981700
H	0.14219600	2.59914700	1.44999100
H	-4.78383400	-0.46565700	0.65698500
C	5.79530200	0.00965800	1.39252900
H	6.35178500	0.92423300	1.59418700
H	5.97082600	-0.72975300	2.17745900
H	6.09268400	-0.41972300	0.43295800
H	-4.40224300	1.15628900	-0.00104300
C	-2.46113900	-0.69069800	-2.32110500
C	-2.21545900	-1.00590800	-3.81203000
O	-1.88564400	-2.08705800	-4.22108700
O	-2.43040600	0.09074700	-4.55049200
H	-2.26782700	-0.15101700	-5.47854900
F	-2.20675500	-1.76806300	-1.57810100
F	-1.66956800	0.31883600	-1.92365100
F	-3.74427300	-0.32623800	-2.13396600

d21

O	3.47481600	-3.03370500	0.17169400
C	3.25315900	-1.94355800	-0.31756400
C	1.89186200	-1.39218800	-0.56651400

C	0.79362600	-2.18848000	-0.21888200
H	0.97865800	-3.16540600	0.21274400
C	-0.49926700	-1.72340200	-0.42571800
H	-4.44063100	0.76270400	-0.31637800
H	-1.35442300	-2.33060900	-0.15567000
O	-3.03911400	-0.70726300	-0.76755200
C	-4.36660200	-0.16974600	-0.87954700
O	4.23093700	-1.09181900	-0.69871400
C	1.68926400	-0.12315000	-1.12534800
H	2.54378200	0.48767800	-1.38903600
O	-2.27682700	1.18066200	-1.74182600
C	-2.06100300	0.10410100	-1.22021200
C	-0.70094600	-0.45600000	-0.98687300
C	0.39651300	0.34062500	-1.33425600
H	0.21004100	1.31988100	-1.75895300
H	-5.02640500	-0.92896800	-0.46133400
C	5.56805900	-1.56831400	-0.48550400
H	6.22317700	-0.77602200	-0.84588500
H	5.74204100	-1.75984700	0.57598500
H	5.73852700	-2.49302600	-1.04171600
H	-4.61567500	0.02584800	-1.92485100
C	-2.04335000	2.40389500	1.36041600
C	-1.89318600	3.06303800	2.74810400
O	-2.70953800	3.80285400	3.22973900
O	-0.74388800	2.67403300	3.31627400
H	-0.69775800	3.09933200	4.18981700
F	-3.13684400	2.86139100	0.75058400
F	-0.96976000	2.65780300	0.59953400
F	-2.15924100	1.06766800	1.50745400

d22

O	3.90328800	-2.06969800	-1.56747300
C	3.52214600	-0.95284900	-1.27745400
C	2.09202000	-0.54084400	-1.21136900
C	1.12208400	-1.50990200	-1.49612900
H	1.44998000	-2.51116700	-1.75072900
C	-0.22745000	-1.18274300	-1.44652200
H	-4.45635500	0.18202100	-0.05753300
H	-0.98434800	-1.92746200	-1.65947000
O	-2.88991400	-0.49873400	-1.24445500
C	-4.27999700	-0.18108300	-1.07197600
O	4.36413600	0.06069000	-0.97635700
C	1.70353000	0.76344100	-0.87689600
H	2.45965900	1.50662000	-0.65587900
O	-2.41948900	1.65951200	-0.78071700
C	-2.04450300	0.53071000	-1.03194900
C	-0.61495200	0.12184500	-1.11507200
C	0.35430700	1.09117200	-0.83065800
H	0.02489400	2.09015600	-0.57047300
H	-4.81750800	-1.11168200	-1.24994000
C	5.75898600	-0.27485700	-1.02435800
H	6.29007100	0.63852200	-0.75887000
H	5.98579400	-1.07350700	-0.31418300
H	6.03904200	-0.60641300	-2.02698200
H	-4.59051100	0.58719400	-1.78362700
C	-2.27561500	0.66557100	2.41265900
C	-2.18322700	0.33959700	3.91858700
O	-2.19516900	-0.77722400	4.36389800
O	-2.10348600	1.47384800	4.62712100
H	-2.06010700	1.22276800	5.56584000

F	-2.25993600	-0.46643500	1.70069200
F	-3.42310000	1.31776300	2.15665600
F	-1.24426200	1.43316900	2.03362800

NaOH

O	2.46511500	1.21158700	1.65754200
H	2.28149800	0.37934200	2.10481500
Na	2.77930600	3.08361500	0.73623900

TS-5

C	-1.39020000	0.63146800	-0.40527700
C	-0.04964800	0.97564600	-0.55316000
C	0.96087100	0.08116500	-0.17920800
C	0.61129900	-1.17925100	0.32719900
C	-0.72726100	-1.52828900	0.47388900
C	-1.73921900	-0.62556600	0.11289100
H	-2.16629600	1.33070300	-0.69246000
H	0.22888100	1.94324200	-0.95499500
H	1.39001300	-1.87697900	0.61082300
H	-1.00259100	-2.50028300	0.86773200
C	2.39428000	0.50580900	-0.34433600
C	-3.15341100	-1.04128200	0.28993300
O	3.22845600	-0.57946400	-0.33348600
O	-4.02780000	-0.09127700	-0.09464700
O	-3.50737500	-2.12352700	0.73713600
O	2.71129600	1.55233100	-0.94597300
C	4.62141200	-0.28289400	-0.47826500
H	4.90333500	0.48697600	0.24555000
H	4.82836000	0.08459700	-1.48822900
C	5.36863800	-1.57794200	-0.21431600

H	5.02918700	-2.35412700	-0.91240100
H	5.17123500	-1.92011200	0.80953100
O	6.76017800	-1.29758300	-0.40037300
H	7.26336700	-2.10339200	-0.22478900
C	-5.42895300	-0.40262200	0.04943000
H	-5.66554300	-1.30259200	-0.52420500
H	-5.65616700	-0.58438100	1.10343000
C	-6.18807600	0.80201800	-0.47759800
H	-5.91538700	1.69546700	0.09797500
H	-5.93118200	0.97221000	-1.53065500
O	-7.57676100	0.49835600	-0.32923400
H	-8.09339200	1.24709900	-0.65504200
O	2.59682900	1.10370100	1.58211800
H	2.19191100	0.44467600	2.15891800
Na	2.73718000	3.12616700	0.75756100

TS-6

C	2.01392200	-0.22343800	-0.25159700
C	0.72373500	0.28919800	-0.34742600
C	0.48400000	1.65338800	-0.13481800
C	1.55851600	2.50295300	0.16673400
C	2.84990100	1.99302400	0.26220300
C	3.08925600	0.62645700	0.05335900
H	2.19185000	-1.27969200	-0.41435600
H	-0.10995700	-0.36681500	-0.57034300
H	1.37756700	3.55879700	0.32746100
H	3.68365800	2.64645100	0.49393600
C	-0.92696200	2.16235800	-0.25738100
C	4.48367000	0.12775600	0.15560800
O	-0.94658600	3.52935200	-0.34872500

O	4.58352200	-1.19295900	-0.09229600
O	5.45173200	0.82304800	0.43201900
O	-1.83595100	1.47368000	-0.76288700
C	-2.24623100	4.11517300	-0.47231400
H	-2.89547800	3.72263300	0.31528600
H	-2.68175800	3.87229700	-1.44679000
C	-2.05963400	5.61497400	-0.32715200
H	-1.35438100	5.97859200	-1.08578700
H	-1.65260800	5.84588400	0.66548100
O	-3.34933900	6.21161800	-0.50202600
H	-3.26388100	7.16785000	-0.39433200
C	5.90341300	-1.76974000	-0.02030800
H	6.56210700	-1.26746900	-0.73380400
H	6.30639800	-1.63777700	0.98751500
C	5.74313000	-3.24047800	-0.36192400
H	5.06718900	-3.71942600	0.35750900
H	5.31492400	-3.34398400	-1.36680600
O	7.05137600	-3.81254900	-0.29847900
H	6.98863300	-4.75179900	-0.51622500
O	-1.34721800	1.95847600	1.71590700
H	-0.56717400	2.22259400	2.21967800
Na	-2.56169900	0.22207800	1.06589200
O	-1.74285500	-2.02425700	0.47712700
C	-2.93767700	-2.36684400	0.32139900
C	-3.13859100	-3.79705600	-0.27582400
F	-2.90970400	-3.78241000	-1.62012100
F	-2.28297200	-4.70485800	0.25692900
F	-4.38734400	-4.28656700	-0.09807100
O	-3.97873700	-1.70593900	0.54122500

INT-9

C	-1.42362500	0.65771200	-0.32098000
C	-0.08507900	1.03488300	-0.40237200
C	0.93003300	0.18455400	0.04989700
C	0.58051700	-1.06365200	0.58591100
C	-0.75459000	-1.45042200	0.66688900
C	-1.76952100	-0.59181400	0.21776400
H	-2.20070100	1.32532200	-0.67443800
H	0.19536500	1.99283300	-0.82573100
H	1.35844700	-1.73487000	0.93117000
H	-1.02432100	-2.41749200	1.07717300
C	2.38538900	0.67840000	-0.01539100
C	-3.17834100	-1.04269900	0.32731700
O	3.18256400	-0.52976000	-0.16674500
O	-4.05674100	-0.12722700	-0.12943600
O	-3.53022100	-2.12346500	0.78119000
O	2.63719700	1.63154000	-0.85695200
C	4.56186500	-0.28996500	-0.41279800
H	4.98893900	0.35931600	0.36240500
H	4.70508100	0.19709000	-1.38420200
C	5.25643800	-1.64078700	-0.39684700
H	4.80532400	-2.29894100	-1.15097200
H	5.13951900	-2.11126300	0.58809700
O	6.64206900	-1.41166100	-0.68733300
H	7.10335600	-2.26037200	-0.67552100
C	-5.45368800	-0.47796100	-0.06089900
H	-5.63452500	-1.38104400	-0.65011800
H	-5.73272200	-0.67186400	0.97830300
C	-6.21740500	0.70772700	-0.62321000
H	-6.00261800	1.60471300	-0.02890500

H	-5.90827100	0.89239900	-1.65965800
O	-7.60303600	0.36385900	-0.55315600
H	-8.12232400	1.10012400	-0.90235900
O	2.70322300	1.18828500	1.37468900
H	2.71698500	0.44116900	1.98973800
Na	3.51841200	3.18508600	0.51974300

INT-10

C	2.39451200	-0.15202000	-0.50861800
C	1.08092300	0.29272800	-0.63673200
C	0.68420300	1.52560900	-0.10625200
C	1.63409800	2.31556100	0.55770500
C	2.95083500	1.88058900	0.68638200
C	3.34245700	0.64140900	0.15750600
H	2.68960400	-1.10888100	-0.92304300
H	0.34151900	-0.30596000	-1.15694500
H	1.34055600	3.27619300	0.96523700
H	3.68660000	2.49352700	1.19562000
C	-0.79449600	1.93402700	-0.23121200
C	4.75382400	0.21549800	0.32059900
O	-0.78256200	3.38872700	-0.24621200
O	4.99954500	-0.99577100	-0.21889700
O	5.61968700	0.87383200	0.88178100
O	-1.47291300	1.36459500	-1.17692500
C	-2.05122300	3.97246200	-0.51191400
H	-2.81065100	3.57943200	0.17578400
H	-2.37252500	3.75417200	-1.53697600
C	-1.89920000	5.47148100	-0.31808400
H	-1.11309600	5.85436400	-0.98216600
H	-1.61246900	5.68861700	0.71912000

O	-3.16340400	6.07299400	-0.63026100
H	-3.09075200	7.02755900	-0.50083600
C	6.34751400	-1.49488000	-0.10540500
H	7.03602200	-0.80872300	-0.60603700
H	6.62460800	-1.56668700	0.94980900
C	6.35230200	-2.86044200	-0.76909200
H	5.64012200	-3.52327700	-0.26182000
H	6.05355700	-2.76502700	-1.82053000
O	7.68770300	-3.35787800	-0.65674900
H	7.72808600	-4.22981600	-1.07108700
O	-1.42855500	1.55617100	1.08932600
H	-1.01892600	2.07181900	1.79837000
Na	-2.91628700	0.10678400	0.03470100
O	-2.69616200	-2.33043700	0.36087200
C	-3.94472700	-2.40111400	0.27490700
C	-4.52143900	-3.84521300	0.43183200
F	-3.97393600	-4.69939400	-0.47326500
F	-4.24457700	-4.34931100	1.66545800
F	-5.86217700	-3.91619100	0.27373900
O	-4.77198500	-1.47924300	0.09157100

PET25

O	5.65755700	16.94398900	-15.07077000
C	5.11284900	17.91518600	-14.57581100
C	4.15106300	17.84826200	-13.43543100
C	3.80716300	16.59440200	-12.92238400
H	1.14013900	14.01987200	-8.42253000
H	4.19311800	15.70429600	-13.40172200
C	2.96394600	16.49839900	-11.82146900
H	-0.59277400	15.73684100	-9.05397300

H	2.70283300	15.53133800	-11.41366200
O	1.27698600	16.31897800	-9.70186000
C	0.36048100	16.04941900	-8.62150000
O	5.31743200	19.18700400	-14.96388500
O	0.06806200	14.64587800	-6.74197000
C	0.98456700	14.91543700	-7.82233000
C	3.63047200	19.00758400	-12.84427600
H	3.89158600	19.97464400	-13.25208300
O	0.93686100	18.56199300	-9.59497500
C	1.48157000	17.59079600	-10.08993400
C	2.44335600	17.65772000	-11.23031400
C	2.78725500	18.91158000	-11.74336100
H	2.40130100	19.80168700	-11.26402400
H	1.93782200	15.22801500	-7.38985700
H	0.20490900	16.94498400	-8.02130000
H	5.95285300	19.20377600	-15.68330100
H	0.41328100	13.93274300	-6.19987900

PET50-B

O	5.65755700	16.94398900	-15.07077000
C	5.11284900	17.91518600	-14.57581100
C	4.15106300	17.84826200	-13.43543100
C	3.80716300	16.59440200	-12.92238400
H	1.14013900	14.01987200	-8.42253000
H	4.19311800	15.70429600	-13.40172200
C	2.96394600	16.49839900	-11.82146900
H	-0.59277400	15.73684100	-9.05397300
H	2.70283300	15.53133800	-11.41366200
O	1.27698600	16.31897800	-9.70186000
C	0.36048100	16.04941900	-8.62150000

O	5.31743200	19.18700400	-14.96388500
O	10.10755700	16.94398900	-15.07077000
C	9.56284900	17.91518600	-14.57581100
C	8.60106300	17.84826200	-13.43543100
C	8.25716300	16.59440200	-12.92238400
H	5.59013900	14.01987200	-8.42253000
H	8.64311800	15.70429600	-13.40172200
C	7.41394600	16.49839900	-11.82146900
H	3.85722600	15.73684100	-9.05397300
H	7.15283300	15.53133800	-11.41366200
O	0.06806200	14.64587800	-6.74197000
C	0.98456700	14.91543700	-7.82233000
C	3.63047200	19.00758400	-12.84427600
H	3.89158600	19.97464400	-13.25208300
O	5.72698600	16.31897800	-9.70186000
O	0.93686100	18.56199300	-9.59497500
C	4.81048100	16.04941900	-8.62150000
C	1.48157000	17.59079600	-10.08993400
C	2.44335600	17.65772000	-11.23031400
C	2.78725500	18.91158000	-11.74336100
H	2.40130100	19.80168700	-11.26402400
H	1.93782200	15.22801500	-7.38985700
H	0.20490900	16.94498400	-8.02130000
O	9.76743200	19.18700400	-14.96388500
O	4.51806200	14.64587800	-6.74197000
C	5.43456700	14.91543700	-7.82233000
C	8.08047200	19.00758400	-12.84427600
H	8.34158600	19.97464400	-13.25208300
O	5.38686100	18.56199300	-9.59497500
C	5.93157000	17.59079600	-10.08993400

C	6.89335600	17.65772000	-11.23031400
C	7.23725500	18.91158000	-11.74336100
H	6.85130100	19.80168700	-11.26402400
H	6.38782200	15.22801500	-7.38985700
H	4.65490900	16.94498400	-8.02130000
H	10.40285300	19.20377600	-15.68330100
H	5.95285300	19.20377600	-15.68330100
H	0.41328100	13.93274300	-6.19987900
H	4.86328100	13.93274300	-6.19987900

PET50-C

O	7.94598000	11.47333800	-15.07077000
C	7.40127200	12.44453600	-14.57581100
C	6.43948500	12.37761100	-13.43543100
C	6.09558600	11.12375200	-12.92238400
H	3.42856200	8.54922100	-8.42253000
H	6.48154000	10.23364500	-13.40172200
C	5.25236900	11.02774800	-11.82146900
H	1.69564900	10.26619000	-9.05397300
H	4.99125500	10.06068800	-11.41366200
O	3.56540900	10.84832700	-9.70186000
C	2.64890400	10.57876800	-8.62150000
O	7.60585500	13.71635400	-14.96388500
O	2.35648500	9.17522700	-6.74197000
C	3.27299000	9.44478600	-7.82233000
O	10.10755700	16.94398900	-15.07077000
C	9.56284900	17.91518600	-14.57581100
C	8.60106300	17.84826200	-13.43543100
C	8.25716300	16.59440200	-12.92238400
C	5.91889500	13.53693300	-12.84427600

H	5.59013900	14.01987200	-8.42253000
H	8.64311800	15.70429600	-13.40172200
H	6.18000900	14.50399300	-13.25208300
O	3.22528400	13.09134200	-9.59497500
C	3.76999200	12.12014500	-10.08993400
C	4.73177900	12.18706900	-11.23031400
C	5.07567800	13.44092900	-11.74336100
C	7.41394600	16.49839900	-11.82146900
H	3.85722600	15.73684100	-9.05397300
H	4.68972400	14.33103600	-11.26402400
H	7.15283300	15.53133800	-11.41366200
H	4.22624500	9.75736500	-7.38985700
H	2.49333200	11.47433300	-8.02130000
O	5.72698600	16.31897800	-9.70186000
C	4.81048100	16.04941900	-8.62150000
O	9.76743200	19.18700400	-14.96388500
O	4.51806200	14.64587800	-6.74197000
C	5.43456700	14.91543700	-7.82233000
C	8.08047200	19.00758400	-12.84427600
H	8.34158600	19.97464400	-13.25208300
O	5.38686100	18.56199300	-9.59497500
C	5.93157000	17.59079600	-10.08993400
C	6.89335600	17.65772000	-11.23031400
C	7.23725500	18.91158000	-11.74336100
H	6.85130100	19.80168700	-11.26402400
H	6.38782200	15.22801500	-7.38985700
H	4.65490900	16.94498400	-8.02130000
H	10.40285300	19.20377600	-15.68330100
H	8.24127500	13.73312500	-15.68330100
H	2.70170400	8.46209300	-6.19987900

H	4.86328100	13.93274300	-6.19987900

PET50-D

O	12.39598000	11.47333800	-15.07077000
C	11.85127200	12.44453600	-14.57581100
C	10.88948500	12.37761100	-13.43543100
C	10.54558600	11.12375200	-12.92238400
H	7.87856200	8.54922100	-8.42253000
H	10.93154000	10.23364500	-13.40172200
C	9.70236900	11.02774800	-11.82146900
H	6.14564900	10.26619000	-9.05397300
H	9.44125500	10.06068800	-11.41366200
O	10.10755700	16.94398900	-15.07077000
C	9.56284900	17.91518600	-14.57581100
C	8.60106300	17.84826200	-13.43543100
C	8.25716300	16.59440200	-12.92238400
H	5.59013900	14.01987200	-8.42253000
H	8.64311800	15.70429600	-13.40172200
O	8.01540900	10.84832700	-9.70186000
C	7.09890400	10.57876800	-8.62150000
C	7.41394600	16.49839900	-11.82146900
H	3.85722600	15.73684100	-9.05397300
H	7.15283300	15.53133800	-11.41366200
O	5.72698600	16.31897800	-9.70186000
C	4.81048100	16.04941900	-8.62150000
O	12.05585500	13.71635400	-14.96388500
O	6.80648500	9.17522700	-6.74197000
C	7.72299000	9.44478600	-7.82233000
O	9.76743200	19.18700400	-14.96388500
C	10.36889500	13.53693300	-12.84427600

H	10.63000900	14.50399300	-13.25208300
O	7.67528400	13.09134200	-9.59497500
C	8.21999200	12.12014500	-10.08993400
C	9.18177900	12.18706900	-11.23031400
C	9.52567800	13.44092900	-11.74336100
H	9.13972400	14.33103600	-11.26402400
O	4.51806200	14.64587800	-6.74197000
C	5.43456700	14.91543700	-7.82233000
H	8.67624500	9.75736500	-7.38985700
H	6.94333200	11.47433300	-8.02130000
C	8.08047200	19.00758400	-12.84427600
H	8.34158600	19.97464400	-13.25208300
O	5.38686100	18.56199300	-9.59497500
C	5.93157000	17.59079600	-10.08993400
C	6.89335600	17.65772000	-11.23031400
C	7.23725500	18.91158000	-11.74336100
H	6.85130100	19.80168700	-11.26402400
H	6.38782200	15.22801500	-7.38985700
H	4.65490900	16.94498400	-8.02130000
H	12.69127500	13.73312500	-15.68330100
H	10.40285300	19.20377600	-15.68330100
H	7.15170400	8.46209300	-6.19987900
H	4.86328100	13.93274300	-6.19987900

PET47

O	5.65755700	16.94398900	-15.07077000
C	5.11284900	17.91518600	-14.57581100
C	4.15106300	17.84826200	-13.43543100
C	3.80716300	16.59440200	-12.92238400
H	1.14013900	14.01987200	-8.42253000

H	4.19311800	15.70429600	-13.40172200
C	2.96394600	16.49839900	-11.82146900
H	-0.59277400	15.73684100	-9.05397300
H	2.70283300	15.53133800	-11.41366200
O	0.40818700	12.40286300	-6.84885500
C	-0.13652100	13.37406000	-6.35389600
C	-1.09830700	13.30713600	-5.21351600
C	-1.44220700	12.05327600	-4.70046900
H	-4.10923100	9.47874600	-0.20061500
H	-1.05625200	11.16316900	-5.17980600
C	-2.28542400	11.95727200	-3.59955400
H	-5.84214400	11.19571400	-0.83205800
H	-2.54653700	10.99021200	-3.19174700
O	1.27698600	16.31897800	-9.70186000
C	0.36048100	16.04941900	-8.62150000
O	-3.97238400	11.77785200	-1.47994500
C	-4.88888800	11.50829300	-0.39958500
O	5.31743200	19.18700400	-14.96388500
O	0.06806200	14.64587800	-6.74197000
C	0.98456700	14.91543700	-7.82233000
O	-5.18130800	10.10475200	1.47994500
C	-4.26480300	10.37431100	0.39958500
C	3.63047200	19.00758400	-12.84427600
H	3.89158600	19.97464400	-13.25208300
O	0.93686100	18.56199300	-9.59497500
C	1.48157000	17.59079600	-10.08993400
C	2.44335600	17.65772000	-11.23031400
C	2.78725500	18.91158000	-11.74336100
H	2.40130100	19.80168700	-11.26402400
C	-1.61889800	14.46645800	-4.62236100

H	1.93782200	15.22801500	-7.38985700
H	-1.35778400	15.43351800	-5.03016800
O	-4.31250900	14.02086700	-1.37306000
C	-3.76780000	13.04967000	-1.86801900
C	-2.80601400	13.11659400	-3.00839900
C	-2.46211500	14.37045400	-3.52144600
H	0.20490900	16.94498400	-8.02130000
H	-2.84806900	15.26056000	-3.04210900
H	-3.31154800	10.68688900	0.83205800
H	-5.04446100	12.40385700	0.20061500
H	5.95285300	19.20377600	-15.68330100
H	-4.83608900	9.39161700	2.02203600

PET94-B

O	9.95447100	2.93865100	0.22805200
C	8.54479600	3.15579700	0.01478300
O	-8.47904900	-1.14164100	-2.02483200
C	-8.18102500	-1.79785500	-1.04224300
C	-6.77885600	-2.11273500	-0.63634700
C	-5.73142200	-1.67368700	-1.45106600
H	0.06175200	-1.03120100	-1.57856500
H	-5.96165300	-1.17534600	-2.38362400
C	-4.41257000	-1.89461900	-1.07150900
H	-0.11207500	-3.41402300	-0.77754600
H	-3.59795500	-1.54786200	-1.69268100
O	2.25019700	-0.34430600	-2.18338500
C	2.54822100	-1.00051900	-1.20079600
C	3.95039000	-1.31540000	-0.79490000
C	4.99782400	-0.87635200	-1.60961900
H	10.79099800	-0.23386500	-1.73711800

H	4.76759300	-0.37801000	-2.54217700
C	6.31667600	-1.09728300	-1.23006200
H	10.61717100	-2.61668700	-0.93609900
H	7.13129100	-0.75052700	-1.85123400
O	-1.84063000	-2.37436700	-0.34643400
C	-0.43095500	-2.59151300	-0.13316500
O	8.88861600	-1.57703200	-0.50498700
C	10.29829100	-1.79417700	-0.29171800
O	-9.06583000	-2.30975100	-0.16756700
O	-10.91723900	2.51209000	-1.31210700
C	-10.61921500	1.85587700	-0.32951800
C	-9.21704600	1.54099600	0.07637800
C	-8.16961200	1.98004400	-0.73834100
H	-2.37643800	2.62253100	-0.86584000
H	-8.39984300	2.47838600	-1.67089900
C	-6.85076000	1.75911200	-0.35878400
H	-2.55026500	0.23970900	-0.06482100
H	-6.03614500	2.10586900	-0.97995600
O	1.66341600	-1.51241600	-0.32612000
O	-0.18799300	3.30942600	-1.47066000
C	0.25374000	-1.29527000	-0.53938900
C	0.11003100	2.65321200	-0.48807100
C	1.51220000	2.33833200	-0.08217500
C	2.55963400	2.77737900	-0.89689400
H	8.35280800	3.41986600	-1.02439300
H	2.32940200	3.27572100	-1.82945200
C	3.87848600	2.55644800	-0.51733700
H	8.17898100	1.03704400	-0.22337400
H	4.69310100	2.90320500	-1.13850900
O	12.39266100	-0.71508000	-0.48467400

C	10.98298600	-0.49793500	-0.69794200
C	-6.49389000	-2.78949900	0.55750800
H	-7.30850500	-3.13625600	1.17867900
O	-4.27882000	1.27936400	0.36629100
O	-2.42741100	-3.54247700	1.51083100
C	-2.86914500	1.06221800	0.57956000
C	-2.72543500	-2.88626400	0.52824200
C	-4.12760400	-2.57138300	0.12234600
C	-5.17503800	-3.01043100	0.93706500
H	-4.94480700	-3.50877300	1.86962300
C	4.23535600	-1.99216400	0.39895500
H	-0.06514000	-0.47276000	0.10499200
H	3.42074100	-2.33892100	1.02012600
O	6.45042600	2.07670000	0.20773800
O	8.30183400	-2.74514200	1.35227800
C	7.86010100	1.85955400	0.42100700
C	8.00381000	-2.08892800	0.36968900
C	6.60164200	-1.77404800	-0.03620700
C	5.55420800	-2.21309500	0.77851200
H	-0.23896600	-2.85558200	0.90601100
H	5.78443900	-2.71143700	1.71107000
H	10.66410600	0.32457500	-0.05356100
H	10.49028000	-2.05824700	0.74745800
O	-11.50402000	1.34398000	0.54515800
O	-0.77477400	2.14131500	0.38660500
C	-2.18445000	2.35846100	0.17333600
C	-8.93208000	0.86423200	1.27023300
H	-9.74669500	0.51747500	1.89140400
O	-4.86560100	0.11125400	2.22355600
C	-5.16362600	0.76746800	1.24096700

C	-6.56579400	1.08234800	0.83507100
C	-7.61322800	0.64330000	1.64979000
H	-7.38299700	0.14495900	2.58234800
C	1.79716600	1.66156700	1.11168000
H	-2.50333000	3.18097100	0.81771700
H	0.98255100	1.31481100	1.73285100
O	5.86364400	0.90858900	2.06500300
C	5.56562000	1.56480300	1.08241400
C	4.16345200	1.87968300	0.67651800
C	3.11601800	1.44063600	1.49123700
H	-2.67715600	0.79814900	1.61873600
H	3.34624900	0.94229400	2.42379500
H	8.22591600	3.97830600	0.65916400
H	8.05209000	1.59548400	1.46018300
H	-12.39462100	1.57672500	0.27265200
H	-9.95643100	-2.07700600	-0.44007300
H	10.44039300	3.73051800	-0.01366400
H	12.87858300	0.07678700	-0.72638900

PET94-C

O	7.57139100	5.00737200	-0.60190400
C	7.40814800	3.90650600	-0.10555600
C	6.07247200	3.31305200	0.20064800
C	4.92797500	4.07567900	-0.04870000
H	-0.88004500	3.70810700	-0.39754900
H	5.03829600	5.09502400	-0.39479400
C	3.66681800	3.53179000	0.16556900
H	-0.48505900	2.55583300	1.80833700
H	2.77755900	4.11353200	-0.03564700
O	-3.15384900	4.18720800	-0.87420700

C	-3.31709300	3.08634200	-0.37786000
C	-4.65276800	2.49288700	-0.07165600
C	-5.79726500	3.25551500	-0.32100300
H	-11.60528600	2.88794300	-0.66985300
H	-5.68694400	4.27485900	-0.66709800
C	-7.05842200	2.71162600	-0.10673500
H	-11.21029900	1.73566800	1.53603400
H	-7.94768100	3.29336800	-0.30795100
O	1.20898300	2.47808700	0.63410500
C	-0.15829800	2.08868100	0.87644200
O	-9.51625800	1.65792200	0.36180100
C	-10.88353800	1.26851600	0.60413800
O	8.40117900	3.05655300	0.21294600
O	-2.32406100	2.23638800	-0.05935800
C	-0.95678000	2.62579400	-0.30169500
O	-13.04930100	1.41622400	-0.33166200
C	-11.68202100	1.80563000	-0.57399900
O	11.30342500	0.48509700	-1.07241600
C	11.14018200	-0.61576900	-0.57606900
C	9.80450700	-1.20922400	-0.26986500
C	8.66001000	-0.44659600	-0.51921200
C	5.94334400	2.00284900	0.68148200
H	2.85198900	-0.81416800	-0.86806200
H	8.77033000	0.57274900	-0.86530700
H	6.83260300	1.42110700	0.88269800
O	2.03877100	0.52726700	1.44895500
C	2.20201500	1.62813300	0.95260700
C	3.53769000	2.22158800	0.64640300
C	4.68218700	1.45896000	0.89575000
C	7.39885200	-0.99048500	-0.30494400

H	3.24697600	-1.96644300	1.33782500
H	4.57186600	0.43961600	1.24184500
H	6.50959400	-0.40874300	-0.50616000
O	0.57818500	-0.33506700	-1.34472000
C	0.41494200	-1.43593400	-0.84837200
C	-0.92073400	-2.02938800	-0.54216800
C	-2.06523100	-1.26676000	-0.79151600
C	-4.78189600	1.18268500	0.40917800
H	-7.87325100	-1.63433200	-1.14036600
H	-0.63001900	2.15864200	-1.23359000
H	-1.95491000	-0.24741600	-1.13761100
H	-3.89263700	0.60094300	0.61039400
O	-8.68646900	-0.29289700	1.17665100
C	-8.52322600	0.80796900	0.68030300
C	-7.18755000	1.40142300	0.37409900
C	-6.04305300	0.63879500	0.62344700
C	-3.32638800	-1.81064900	-0.57724800
H	-0.23503300	1.00636700	0.97229600
H	-7.47826500	-2.78660700	1.06552100
H	-6.15337400	-0.38054900	0.96954200
H	-4.21564600	-1.22890700	-0.77846400
H	-11.35525900	1.33847800	-1.50589400
H	-10.96027300	0.18620300	0.69999300
O	4.94101700	-2.04418800	0.16359200
C	3.57373700	-2.43359400	0.40592900
O	-5.78422300	-2.86435300	-0.10871100
C	-7.15150400	-3.25375900	0.13362500
O	12.13321400	-1.46572300	-0.25756700
O	1.40797400	-2.28588700	-0.52987000
C	2.77525400	-1.89648100	-0.77220700

O	-9.31726700	-3.10605200	-0.80217400
C	-7.94998600	-2.71664600	-1.04451100
C	9.67537900	-2.51942600	0.21097000
H	10.56463700	-3.10116800	0.41218600
O	5.77080600	-3.99500800	0.97844200
C	5.93404900	-2.89414200	0.48209400
C	7.26972400	-2.30068800	0.17589000
C	8.41422100	-3.06331500	0.42523800
H	8.30390100	-4.08266000	0.77133300
C	-1.04986100	-3.33959100	-0.06133400
H	3.10201500	-2.36363300	-1.70410300
H	-0.16060300	-3.92133300	0.13988200
O	-4.95443500	-4.81517300	0.70613800
C	-4.79119100	-3.71430600	0.20979000
C	-3.45551600	-3.12085200	-0.09641300
C	-2.31101900	-3.88348000	0.15293400
H	3.49700200	-3.51590800	0.50178400
H	-2.42134000	-4.90282400	0.49902900
H	-7.62322500	-3.18379700	-1.97640700
H	-7.22823900	-4.33607200	0.22948000
H	12.97818000	-1.06908200	-0.48186400
H	9.24614500	3.45319300	-0.01135100
H	-13.60370000	1.73105500	-1.04938300
H	-9.87166500	-2.79122000	-1.51989500

PET94-D

O	-11.86330200	2.90979900	-0.25229400
C	-10.48249700	3.31906600	-0.32777900
O	8.92825900	5.30344000	0.15245400
C	8.70972900	4.11940700	-0.03523200

C	7.34912300	3.50414100	-0.02196100
C	6.24978200	4.31663200	0.26951200
H	0.42153000	4.41342600	0.15988700
H	6.41538200	5.35120500	0.54016100
C	4.96355800	3.79105100	0.22906400
H	0.81606700	2.21430200	1.32517000
H	4.10840500	4.41754300	0.44333900
O	-1.83071800	5.15766700	0.18017600
C	-2.04924800	3.97363400	-0.00751100
C	-3.40985400	3.35836800	0.00576100
C	-4.50919500	4.17085900	0.29723400
H	-10.33744700	4.26765200	0.18760800
H	-4.34359500	5.20543200	0.56788200
C	-5.79541900	3.64527800	0.25678500
H	-9.94291000	2.06852900	1.35289200
H	-6.65057200	4.27177000	0.47106100
O	10.19949800	-0.48775900	0.25668600
C	9.98096800	-1.67179200	0.06900000
C	8.62036100	-2.28705800	0.08227100
C	7.52102100	-1.47456700	0.37374400
H	1.69276900	-1.37777300	0.26411900
H	7.68662100	-0.43999400	0.64439300
O	2.45907000	2.74356800	0.19598400
C	1.07826600	2.33430000	0.27146900
C	6.23479700	-2.00014800	0.33329600
H	2.08730600	-3.57689700	1.42940200
H	5.37964400	-1.37365600	0.54757100
O	-0.55947900	-0.63353200	0.28440800
C	-0.77800900	-1.81756500	0.09672200
C	-2.13861500	-2.43283100	0.10999300

C	-3.23795600	-1.62034000	0.40146600
H	-9.06620800	-1.52354700	0.29184000
H	-3.07235600	-0.58576700	0.67211500
O	-8.29990600	2.59779500	0.22370600
C	-9.68071100	2.18852700	0.29919100
C	-4.52418000	-2.14592200	0.36101800
H	-8.67167100	-3.72267000	1.45712400
H	-5.37933300	-1.51942900	0.57529300
O	3.73030900	-3.04763100	0.30021600
C	2.34950500	-3.45689900	0.37570100
O	-7.02866700	-3.19340400	0.32793800
C	-8.40947200	-3.60267200	0.40342300
O	9.65465200	3.20134500	-0.30773800
O	-1.10432500	3.05557200	-0.28001600
C	0.27648000	3.46484000	-0.35550100
O	10.92589100	-2.58985400	-0.20350500
C	7.15016500	2.15386200	-0.34081700
H	8.00531700	1.52737000	-0.55509300
O	3.18546300	0.64147300	-0.26420800
C	3.40399400	1.82550600	-0.07652100
C	4.76460000	2.44077200	-0.08979300
C	5.86394100	1.62828100	-0.38126600
H	5.69834000	0.59370800	-0.65191400
O	0.16691400	-2.73562700	-0.17578400
C	1.54771900	-2.32635900	-0.25126900
C	-3.60881200	2.00808900	-0.31309600
H	0.53867900	3.58483800	-1.40920200
H	-2.75366000	1.38159700	-0.52737100
O	-7.57351300	0.49570000	-0.23648600
C	-7.35498300	1.67973300	-0.04880000

C	-5.99437700	2.29499900	-0.06207100
C	-4.89503600	1.48250800	-0.35354400
H	0.93321600	1.38571400	-0.24391900
H	-5.06063700	0.44793500	-0.62419300
O	-10.59206300	-2.88140100	-0.14806200
C	-9.21125800	-2.47213300	-0.22354700
H	-10.22029800	3.43906400	-1.38148000
H	-9.82576100	1.23994100	-0.21619700
C	8.42140400	-3.63733700	-0.23658500
H	9.27655600	-4.26382900	-0.45086100
O	4.45670200	-5.14972600	-0.15997600
C	4.67523300	-3.96569300	0.02771100
C	6.03583900	-3.35042700	0.01443900
C	7.13518000	-4.16291800	-0.27703400
H	6.96957900	-5.19749100	-0.54768200
C	-2.33757300	-3.78311000	-0.20886400
H	1.80991800	-2.20636200	-1.30497000
H	-1.48242100	-4.40960200	-0.42313900
O	-6.30227400	-5.29549900	-0.13225400
C	-6.08374400	-4.11146600	0.05543300
C	-4.72313800	-3.49620000	0.04216100
C	-3.62379700	-4.30869100	-0.24931200
H	2.20445500	-4.40548500	-0.13968600
H	-3.78939800	-5.34326400	-0.51996000
H	-8.94905900	-2.35213500	-1.27724800
H	-8.55452200	-4.55125800	-0.11196500
H	11.78696700	-2.16598700	-0.22551900
H	10.51572800	3.62521200	-0.32975200
H	-12.42034200	3.59068100	-0.63661700
H	-11.14910300	-2.20051800	-0.53238500

Proposed mechanism.

Figure S37. Proposed possible mechanism

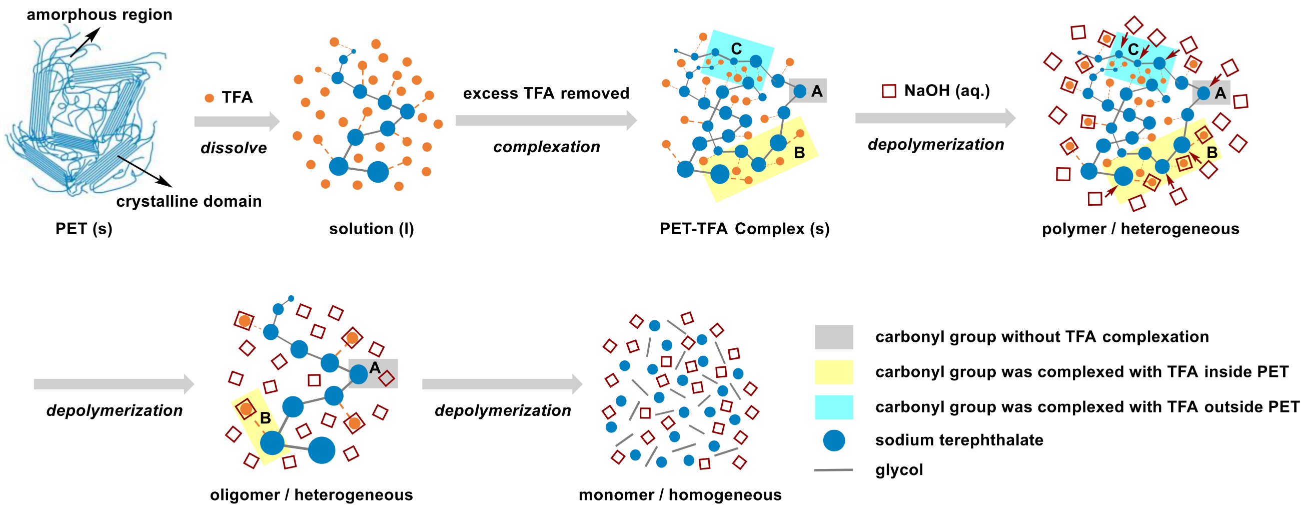

VI. Characterization of the polyesters depolymerization products.

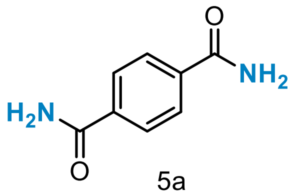
5a

Terephthalamide (5a). Prepared following separation and purification of **5a**, TFA-treated PET was obtained by general procedure B, then NH_4OH (2 mL) was added to depolymerize TFA-treated PET, depolymerization of TFA-treated PET takes approximately 72 h to obtain white suspension (**5a** saturated in NH_4OH). filtered under vacuum removed excess NH_4OH obtained the residue, the residue was dissolved in MeOH and then quenching with silica gel (note: the analysis of TLC before quenching). After evaporation of MeOH under vacuum, the residue was purified by column chromatography to yield the corresponding **5a** (yield 31%, white solid). **^{1}H NMR** (500 MHz, DMSO-d_6), δ 8.07 (s, 2H), 7.93 (s, 4H), 7.49, (s, 2H). **^{13}C NMR** (126 MHz, DMSO-d_6), δ 167.3, 136,6, 127.4. The spectral data reported in the literature.[18]

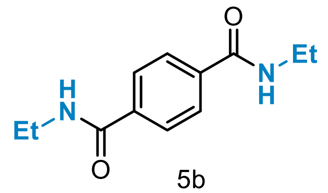
5b

***N^1,N^4*-Diethylterephthalamide (5b).** Prepared following General procedure B, depolymerization of TFA-treated PET takes approximately 24 h to obtain white suspension (**5b** saturated in ethylamine). Filtered under vacuum removed excess ethylamine obtained the residue, followed by quenching with silica gel (note: the analysis of TLC before quenching). After evaporation of solvent under vacuum, the residue was purified by column chromatography to yield the corresponding product **5b** (yield: 85%, white solid). **^{1}H NMR** (500 MHz, DMSO-d_6), δ 8.07 (t, J = 5.5 Hz, 2H), 7.88 (s, 4H), 3.31 - 3.26 (m, 4H), 1.12 (t, J = 7.0 Hz, 6H). **^{13}C NMR** (126 MHz, DMSO-d_6), δ 165.6, 136.9, 127.2, 34.3, 14.9. **HRMS** (ESI) m/z: $[M+H]^+$ Calcd for $C_{12}H_{17}N_2O_2$ 221.1285, found 258.1283. **IR** (neat) 3302, 2977, 1637, 1546, 1311, 1286, 855, 596 cm^{-1}.

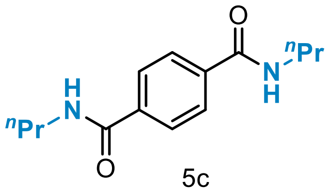
5c

***N^1,N^4*-Dipropylterephthalamide (5c).** Prepared following General procedure B, depolymerization of TFA-treated PET takes approximately 24 h to obtain white suspension (**5c**

saturated in *n*-Propylamine). Filtered under vacuum removed excess *n*-Propylamine obtained the residue, followed by quenching with silica gel (note: the analysis of TLC before quenching). After evaporation of solvent under vacuum, the residue was purified by column chromatography to yield the corresponding product **5c** (yield 93%, white solid). **^{1}H NMR** (500 MHz, DMSO-d_6), δ 8.55 (t, J = 6.0 Hz, 2H), 7.90 (s, 4H), 3.25 - 3.20 (m, 4H), 1.58 – 1.50 (m, 4H), 0.89 (t, J = 7.5 Hz, 6H). **^{13}C NMR** (126 MHz, DMSO-d_6), δ 165.5, 136.8, 127.1, 41.0, 22.4, 11.5. **HRMS** (ESI) m/z: $[M+H]^+$ Calcd for $C_{14}H_{21}N_2O_2$ 249.1598, found 249.1596. **IR** (neat) 3299, 2961, 1635, 1539, 1328, 1277, 861, 668 cm^{-1}.

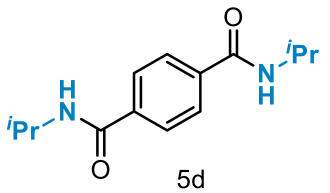

5d

***N^1,N^4*-Diisopropylterephthalamide (5d).** Prepared following separation and purification of **5d**, TFA-treated PET was obtained by general procedure B, then TBD (104 mg, 0.75 mmol, 3 equiv.) and *i*-propylamine (2 mL) was added to depolymerize TFA-treated PET, completely depolymerization of TFA-treated PET takes approximately 72 h to obtain white suspension (**5d** saturated in *i*-propylamine), followed petroleum ether was added and then filtered under vacuum removed excess *i*-propylamine obtained the residue, the residue was purified by recrystallization to yield the corresponding **5d** (yield 78%, recrystallization solvent, DCM/PE, white solid). **^{1}H NMR** (500 MHz, DMSO-d_6), δ 8.34 (d, J = 8.0 Hz, 2H), 7.90 (s, 4H), 4.15 - 4.06 (m, 2H), 1.17 (d, J = 7.0 Hz, 12H). **^{13}C NMR** (126 MHz, DMSO-d_6), δ 164.7, 136.8, 127.1, 41.1, 22.3. **HRMS** (ESI) m/z: $[M+H]^+$ Calcd for $C_{14}H_{21}N_2O_2$ 249.1598, found 249.1599. **IR** (neat) 3330, 1781, 1717, 1629, 1342, 1254, 1160, 1110, 730 cm^{-1}.

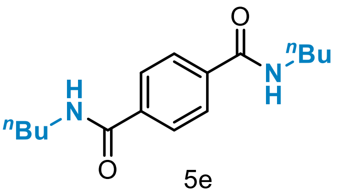

5e

***N^1,N^4*-Dibutylterephthalamide (5e).** Prepared following general procedure B, depolymerization of TFA-treated PET takes approximately 24 h to obtain white suspension (**5e** saturated in *n*-butylamine). Filtered under vacuum removed excess *n*-butylamine obtained the residue, followed by quenching with silica gel (note: the analysis of TLC before quenching). After evaporation of solvent under vacuum, the residue was

purified by column chromatography to yield the corresponding product **5e** (yield 83%, white solid). **^{1}H NMR** (400 MHz, DMSO-d_6), δ 8.56 (t, J = 5.6 Hz, 2H), 7.89 (s, 4H), 3.29 - 3.24 (m, 4H), 1.54 – 1.47 (m, 4H), 1.37 – 1.29 (m, 4H), 0.90 (t, J = 7.2 Hz, 6H). **^{13}C NMR** (101 MHz, DMSO-d_6), δ 165.5, 136.8, 127.1, 39.0, 31.3, 19.7, 13.8. **HRMS** (ESI) m/z: $[M+H]^+$ Calcd for $C_{16}H_{25}N_2O_2$ 277.1911, found 277.1912. **IR** (neat) 3297, 1654, 1637, 1550, 1340, 690, 668 cm^{-1}.

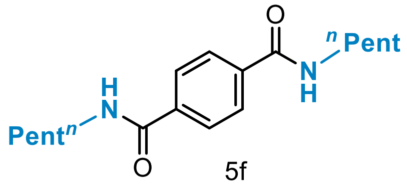

5f

***N^1,N^4*-Dipentylterephthalamide (5f).** Prepared following general procedure B, depolymerization of TFA-treated PET takes approximately 24 h to obtain white suspension (**5f** saturated in *n*-amylamine). Filtered under vacuum removed excess *n*-amylamine obtained the residue, followed by quenching with silica gel (note: the analysis of TLC before quenching). After evaporation of solvent under vacuum, the residue was purified by column chromatography to yield the corresponding product **5f** (yield 80%, white solid). **^{1}H NMR** (400 MHz, DMSO-d_6), δ 8.53 (t, J = 5.6 Hz, 2H), 7.89 (s, 4H), 3.28 - 3.23 (m, 4H), 1.56 – 1.49 (m, 4H), 1.34 – 1.26 (m, 8H), 0.87 (t, J = 6.8 Hz, 6H). **^{13}C NMR** (101 MHz, DMSO-d_6), δ 165.4, 136.7, 127.0, 39.2, 28.8, 28.7, 21.9, 13.9. The spectral data reported in the literature.[19]

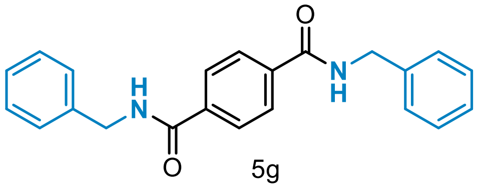

5g

***N^1,N^4*-Dibenzylterephthalamide (5g).** Prepared following separation and purification of **5g**, TFA-treated PET was obtained by general procedure B, then TBD (104 mg, 0.75 mmol, 3 equiv.) and *i*-propylamine (2 mL) was added to depolymerize TFA-treated PET, completely depolymerization of TFA-treated PET takes approximately 72 h to obtain white suspension (**5g** saturated in benzylamine), followed petroleum ether was added and then filtered under vacuum removed excess benzylamine obtained the residue, the residue was purified by recrystallization to yield the corresponding **5g** (yield 92%, recrystallization solvent, DCM/PE, white solid). **^{1}H NMR** (500 MHz, DMSO-d_6), δ 9.19 (t, J = 6.0 Hz, 2H), 7.99 (s, 4H), 7.35 –

7.31 (m, 8H), 7.26 - 7.23 (m, 2H), 4.50 (d, J = 4.0 Hz 4H). **^{13}C NMR** (126 MHz, DMSO-d_6), δ 165.6, 139.5, 136.6, 128.3, 127.3, 127.3, 126.8, 42.7. **HRMS** (ESI) m/z: $[M+H]^+$ Calcd for $C_{22}H_{21}N_2O_2$ 345.1598, found 345.1600. **IR** (neat) 3278, 1717, 1627, 1534, 1252, 1127, 864, 580 cm^{-1}.

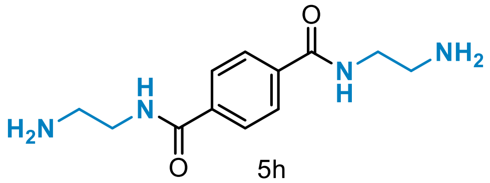

5h

***N*^1,*N*^4-Bis(2-aminoethyl)terephthalamide (5h).** Prepared following separation and purification of **5h**, TFA-treated PET was obtained by general procedure B, then ethylenediamine (2 mL) was added to depolymerize TFA-treated PET, completely depolymerization of TFA-treated PET to form 5h dissolved in ethylenediamine, followed excess ethylenediamine was removed by vacuum distillation, the residue was washed by 10 mL MeOH/DCM (v/v = 2:3), and then purified by recrystallization to yield the corresponding **5h** (yield 90%, recrystallization solvent, H_2O/CH_3CN, white solid). **^{1}H NMR** (400 MHz, D_2O), δ 7.92 (s, 4H), 3.76 (t, J = 6.0 Hz, 4H), 3.30 (t, J = 6.0 Hz, 4H). **^{13}C NMR** (101 MHz, D_2O), δ 170.6, 136.4, 127.6, 39.3, 37.5. **HRMS** (ESI) m/z: $[M+H]^+$ Calcd for $C_{12}H_{19}N_4O_2$ 251.1503, found 251.1502. **IR** (neat) 3238, 2901, 1717, 1635, 1555, 1251, 1176, 1104, 1022, 868, 700 cm^{-1}.

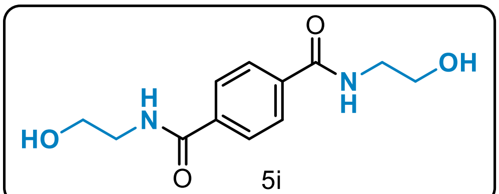

5i

***N*^1,*N*^4-Bis(2-hydroxyethyl)terephthalamide (5i).** Prepared following separation and purification of **5i,** TFA-treated PET was obtained by general procedure B, then ethanolamine (2 mL) was added to depolymerize TFA-treated PET, completely depolymerization of TFA-treated PET to form 5i dissolved in ethanolamine, followed DCM was added into the reaction solution, the residue was precipitated from the reaction solution, and then purified by recrystallization to yield the corresponding **5i** (yield 81%, recrystallization solvent, MeOH/DCM, white solid). **^{1}H NMR** (500 MHz, DMSO-d_6), δ 8.54 (t, J = 5.5 Hz, 2H), 7.91 (s, 4H), 4.74 (s, 2H), 3.52 (t, J = 6.0 Hz, 4H), 3.36 – 3.33 (m, 4H). **^{13}C NMR** (126 MHz, DMSO-d_6), δ 165.7, 136.7, 127.1, 59.7, 42.3. **HRMS** (ESI) m/z: $[M+H]^+$ Calcd for $C_{12}H_{17}N_2O_4$ 253.1183, found 253.1182. **IR** (neat)

3280, 2968, 1717, 1631, 1554, 1252, 1210, 1124, 1093, 839, 728 cm^{-1}.

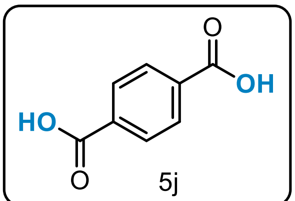

5j

Terephthalic acid (**5j**). Prepared following general procedure D, completely depolymerization of TFA-treated PET to sodium terephthalate dissolved in NaOH (aq.), it takes approximately 6 h to form yellowish clarified solution, TPA was precipitated from the clarified solution when used HCl (12 N) adjust pH = 3. Finally, TPA was separated by vacuum filtration and dried in 80°C oven. (yield 98%, white solid). **^{1}H NMR** (400 MHz, DMSO-d_6), δ 13.31 (s, 2H), 8.03 (s, 4H). **^{13}C NMR** (101 MHz, DMSO-d_6) δ 166.7, 134.5, 129.5. The spectral data reported in the literature.[20]

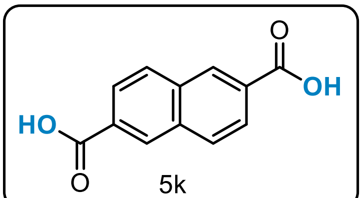

5k

2,6-Naphthalenedic arboxylic acid (**5k**). Prepared following general procedure F, completely depolymerization of TFA-treated PET to sodium naphthalene-2,6-dicarboxylate dissolved in NaOH (aq.), it takes approximately 12 h to form clarified solution, sodium naphthalene-2,6-dicarboxylate was precipitated from the clarified solution when used HCl (12 N) adjust pH = 3. Finally, naphthalene-2,6-dicarboxylic acid (NDA) was separated by vacuum filtration and dried in 80°C oven (yield 93%, white solid). **^{1}H NMR** (500 MHz, DMSO-d_6), δ 13.24 (s, 2H), 8.66 (s, 2H), 8.19 – 8.18 (m, 2H), 8.05 – 8.03 (m, 2H), **^{13}C NMR** (126 MHz, DMSO-d_6) δ 167.2, 134.2, 130.2, 130.1, 129.7, 125.9, The spectral data reported in the literature.[21]

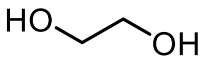

6a

Ethylene glycol (**6a**). Prepared following general procedure E, after TPA was precipitated from the clarified solution when used HCl (12 N) adjust pH = 3. filtered under vacuum separated TPA to obtain white clarified filtration solution. then water and TFA were separated by vacuum distillation with rotary evaporator to obtain 6a and NaCl (s), filtered under vacuum and then washed NaCl(s) with DCM/EtOH (v/v = 1:1) to obtain mixture filtration solution of 6a and DCM/EtOH, DCM/EtOH was removed by vacuum distillation to obtain crude **6a** (Contains a small

amount of TFA), finally, **6a** was separated and purified by vacuum distillation (colorless liquid). **^{1}H NMR** (400 MHz, DMSO-d_6), δ 4.66 (s, 2H), 3.40 (s, 4H). **^{13}C NMR** (101 MHz, DMSO-d_6) δ 62.9, The spectral data reported in the literature.[22]

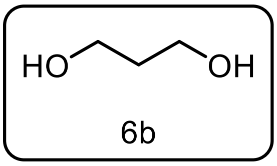

Propanediol (**6b**). Prepared following general procedure E, after TPA was precipitated from the clarified solution when used HCl (12 N) adjust pH = 3. filtered under vacuum separated TPA to obtain white clarified filtration solution, then water and TFA were separated by vacuum distillation with rotary evaporator to obtain **6b** and NaCl (s), filtered under vacuum and then washed NaCl(s) with DCM/EtOH (v/v = 1:1) to obtain mixture filtration solution, then DCM/EtOH was removed by vacuum distillation to obtain crude **6b**, finally, **6b** was separated and purified by vacuum distillation (colorless liquid). **^{1}H NMR** (500 MHz, D_2O), δ 3.62 (t, *J* = 5.5 Hz, 2H), 1.75 – 1.70(m, 4H). **^{13}C NMR** (126 MHz, DMSO-d_6) δ 58.5, 33.7. The spectral data reported in the literature.[23]

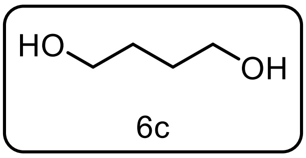

Butane-1,4-diol (**6c**). Prepared following general procedure E, after TPA was precipitated from the clarified solution when used HCl (12 N) adjust pH = 3. filtered under vacuum separated TPA to obtain light clarified filtration solution, then water and TFA were separated by vacuum distillation with rotary evaporator to obtain **6c** and NaCl (s), filtered under vacuum and then washed NaCl(s) with DCM/EtOH (v/v = 1:1) to obtain mixture filtration solution of, then DCM/EtOH was removed by vacuum distillation to obtain crude **6c** (Contains a small amount of TFA), finally, **6c** was separated and purified by vacuum distillation (colorless liquid). **^{1}H NMR** (500 MHz, D_2O), δ 3.71 – 3.69 (m, 4H), 1.68 – 1.66 (m, 4H), **^{13}C NMR** (126 MHz, DMSO-d_6) δ 61.7, 28.0. The spectral data reported in the literature.[24]

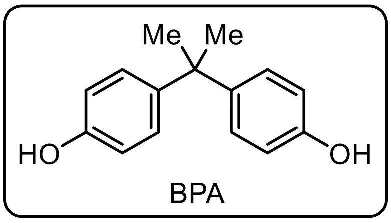

4,4'-(Propane-2,2-diyl)diphenol (**BPA**). Prepared following

general procedures G for the hydrolysis of PET/PC blend and PBT/PC blend, completely depolymerization of PET or PBT and PC to sodium terephthalate and sodium 4,4'-(propane-2,2-diyl)diphenolate dissolved in NaOH (aq.), it takes approximately 12 h, TPA and bisphenol A were precipitated from the clarified filtrate when used HCl (12 N) adjust pH = 3, followed TPA and bisphenol A were separated by vacuum filtration, bisphenol A was separated and purified by DCM/EtOH (v/v = 1:1) (yield 90%, white solid). **^{1}H NMR** (500 MHz, DMSO-d_6), δ 9.20 (s, 2H), 6.99 (d, J = 8.5 Hz, 4H), 6.66 (d, J = 8.5 Hz, 4H), 1.53 (s, 6H). **^{13}C NMR** (126 MHz, DMSO-d_6) δ 155.0, 141.3, 127.5, 114.7, 41.1, 31.0. The spectral data reported in the literature.[25]

VII. References

1) M. J. Frisch, G. W. Trucks, H. B. Schlegel, G. E. Scuseria, M. A. Robb, J. R. Cheeseman, G. Scalmani, V. Barone, G. A. Petersson, H. Nakatsuji, X. Li, M. Caricato, A. V. Marenich, J. Bloino, B. G. Janesko, R. Gomperts, B. Mennucci, H. P. Hratchian, J. V. Ortiz, A. F. Izmaylov, J. L. Sonnenberg, D. Williams-Young, F. Ding, F. Lipparini, F. Egidi, J. Goings, B. Peng, A. Petrone, T. Henderson, D. Ranasinghe, V. G. Zakrzewski, J. Gao, N. Rega, G. Zheng, W. Liang, M. Hada, M. Ehara, K. Toyota, R. Fukuda, J. Hasegawa, M. Ishida, T. Nakajima, Y. Honda, O. Kitao, H. Nakai, T. Vreven, K. Throssell, J. A. Montgomery, J. E. Peralta, F. Ogliaro, M. J. Bearpark, J. J. Heyd, E. N. Brothers, K. N. Kudin, V. N. Staroverov, T. A. Keith, R. Kobayashi, J. Normand, K. Raghavachari, A. P. Rendell, J. C. Burant, S. S. Iyengar, J. Tomasi, M. Cossi, J. M. Millam, M. Klene, C. Adamo, R. Cammi, J. W. Ochterski, R. L. Martin, K. Morokuma, O. Farkas, J. B. Foresman, D. J. Fox, Gaussian, Inc., Wallingford CT, **2016**.

2) a) S. H. Vosko, L. Wilk, M. Nusair, *Can. J. Phys.* **1980**, *58*, 1200−1211; b) A. D. Becke, *Phys. Rev. A* **1988**, *38*, 3098−3100; c) C. Lee, W. Yang, R. G. Parr, *Phys. Rev. B* **1988**, *37*, 785−789; d) A. D. Becke, *J. Chem. Phys.* **1993**, *98*, 5648−5652; e) S. Grimme, J. Antony, S. Ehrlich, H. Krieg, *J. Chem. Phys.* **2010**, *132*, 154104.

3) a) S. Miertuš, E. Scrocco, J. Tomasi, *Chem. Phys.* **1981**, *55*, 117-129; b) S. Miertuš, J. Tomasi, *Chem. Phys.* **1982**, 65, 239-245; c) J. L. Pascual-Ahuir, E. Silla, I. Tuñón, *J. Comp. Chem.* **1994**, *15*, 1127-1138; d) J. Tomasi, B. Mennucci, R. Cammi, *Chem. Rev.* **2005**, *105*, 2999-3093.

4) a). R. Ditchfield, W. J. Hehre, J. A. Pople, *J. Chem. Phys.* **1971**, *54*, 724-728, b) T. Clark, J. Chandrasekhar, G. W. Spitznagel, P. v. R. Schleyer, *J. Comp. Chem.* **1983**, *4*, 294-301. c) M. J. Frisch, J. A. Pople, J. S. Binkley, *J. Chem. Phys.***1984**, *80*, 3265-3269.

5) a) K. Fukui, *Acc. Chem. Res.* **1981**, *14*, 363-368.

6) a) Y. Zhao, D. G. Truhlar, *Theor. Chem. Acc.* **2008,** *120*, 215-241; b) S. Grimme, J. Antony, S. Ehrlich, H. Krieg, *J. Chem. Phys.* **2010,** *132*, 154104.

7) a) K. Raghavachari, J. S. Binkley, R. Seeger, J. A. Pople, *J. Chem. Phys.* **1980**, *72*, 650-654; b) A. D. McLean, G. S. Chandler, *J. Chem. Phys.* **1980**, *72*, 5639-5648; c) T. Clark, J. Chandrasekhar, G. W. Spitznagel, P. v. R. Schleyer, *J. Comp. Chem.* **1983**, *4*, 294-301; d) M. J. Frisch, J. A. Pople, J. S. Binkley, *J. Chem. Phys.* **1984**, *80*, 3265-3269.

8) A. V. Marenich, C. J. Cramer, D. G. Truhlar, *J. Phys. Chem. B* **2009**,*113*, 6378-6396.
9) W. Humphrey, A. Dalke, K. Schulten, *J. Mol. Graphics* **1996**,*14*, 33-38.
10) T. Lu, F. Chen, *J. Comput. Chem.* **2012**, *33*, 580-592.
11) T. Lu, Q. Chen, *J. Compu. Chem.* **2022**, *43*, 539-555.
12) T. Kurita, Y. Fukuda, M. Takahashi, Y. Sasanuma, *ACS Omega* **2018**, *3*, 4824-4835.
13) a) S. Grimme, C. Bannwarth, P. Shushkov, *J. Chem. Theory Comput.* **2017**, *13*, 1989-2009; b) C. Bannwarth, S. Ehlert, S. Grimme, *J. Chem. Theory Comput.* **2019**, *15*, 1652-1671; c)
14) T. Lu, Molclus program, Version x.x, http://www.keinsci.com/research/molclus.html.
15) T. Lu, Sobtop, Version 1.0 (dev3.1), http://sobereva.com/soft/Sobtop.
16) M. J. Abraham, T. Murtola, R. Schulz, S. Páll, J. C. Smith, B. Hess, E. Lindahl, *SoftwareX*, **2015**, *1-2*, 19-25.
17) S. Emamian, T. Lu, H. Kruse, H. Emamian, *J. Comput. Chem.* **2019**, *40*, 2868-2881.
18) a) A. Naraoka, H. Naka, *Synlett* **2019**, *30*, 1977-1980; b) T. Tu, Z. Wang, Z. Liu, X. Feng, Q. Wang, *Green Chem.* **2012**, *14*, 921-924.
19) M. Kawana, R. Mizoue, T. Takeda, N. Hoshino, T. Akutagawa, *J. Mater. Chem. C* **2022**,*10*, 4208-4217.
20) a) A. K. Jaiswal, A. K. Kushawaha, S. Pandey, A. Kumar, K. V. Sashidhara, Tetrahedron **2023**, *136* 133359; b) J. Meng, Y. Zhou, D. Li, X. Jiang, *Sci. Bull.* **2023**, *68* 1522-1530.
21) M. S. Krivosheina, R. S. Borisov, D. I. Zhilyaev, M. D. Matveeva, V. G. Zaikin, *Rapid Commun. Mass Spectrom.* **2021**, *35*, e8954.
22) F. Ono, H. Takenaka, T. Fujikawa, M. Mori, T. Sato, *Synthesis* **2009**, *8*, 1318-1322.
23) V. H. Jadhav, J. G. Kim, H. J. Jeong, D. W. Kim, *J. Org. Chem.* **2015**, *80*, 7275-7280.
24) A. Nalikezhathu, A. Tam, V. Cherepakhin, V. K. Do, T. J. Williams, *Org. Lett.* **2023**, *25*, 1754-1759.
25) K. Sun, Q. Liu, J. Liu, S. Li, X. Qi, M. Chen, Y. Si, Y. Gao, *J. Hazard. Mater.* **2022**, *436*, 129269.

VIII. NMR Spectra

Compound **5a** ^{1}H NMR

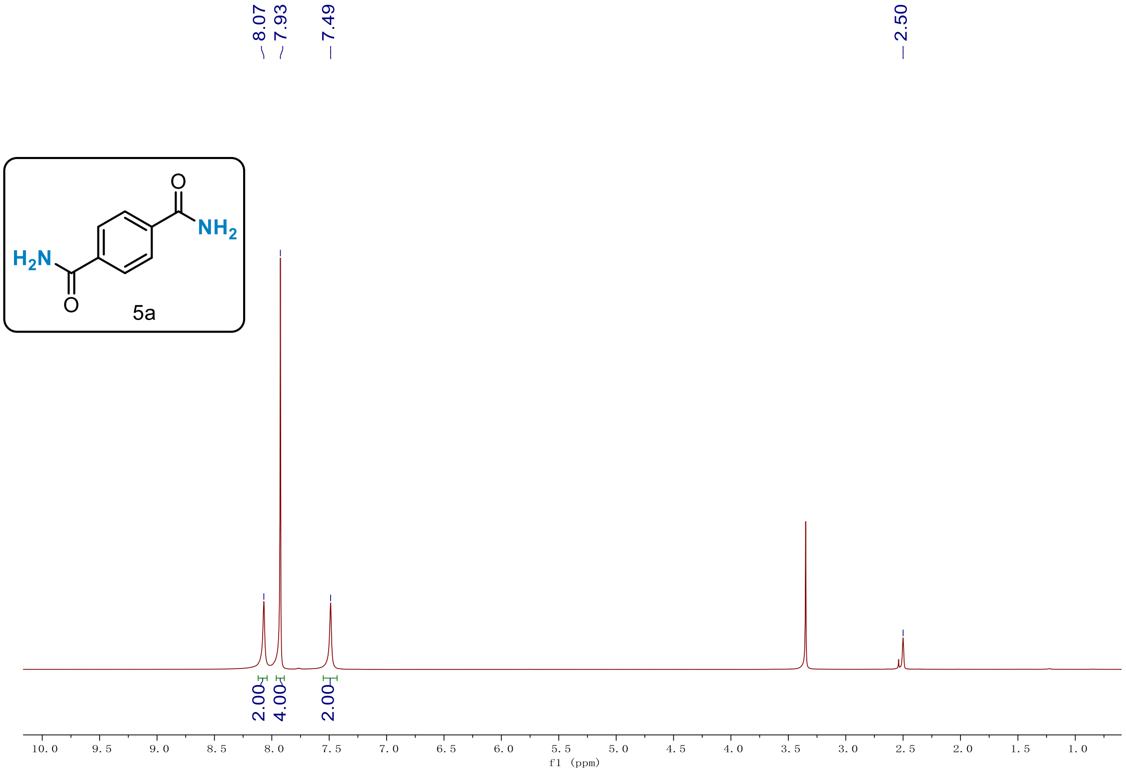

Compound **5a** ^{13}C NMR

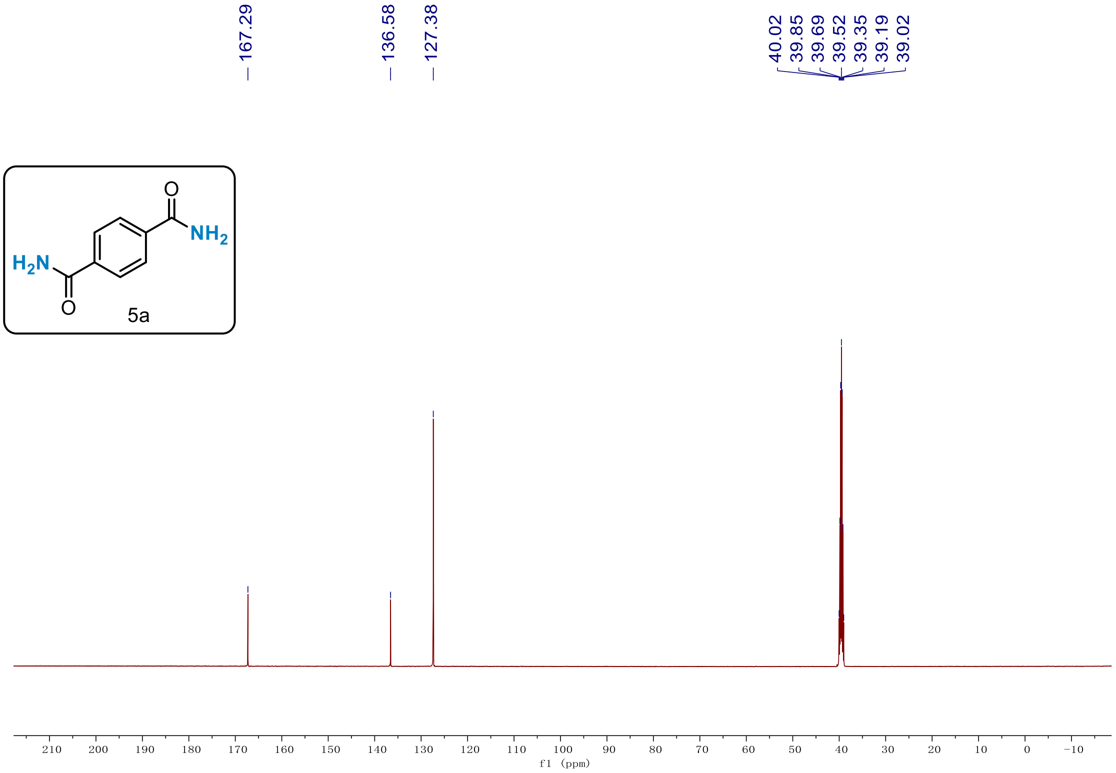

Compound **5b** ^{1}H NMR

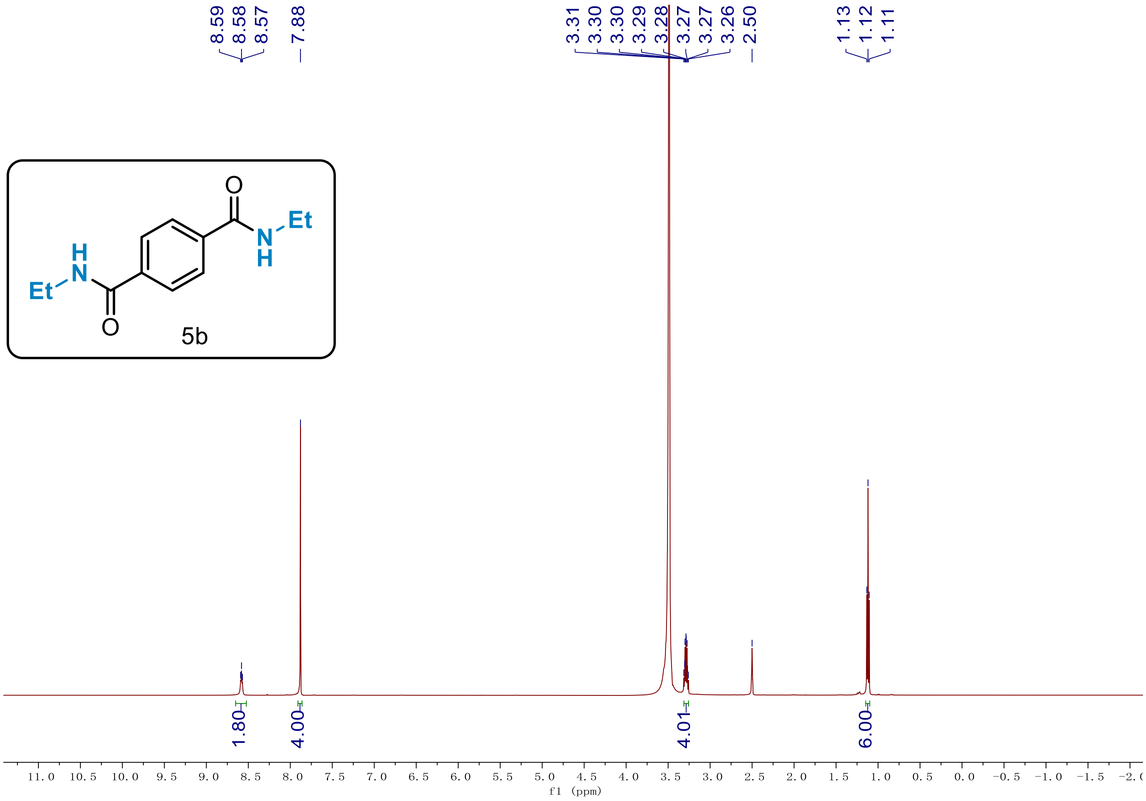

Compound **5b** ^{13}C NMR

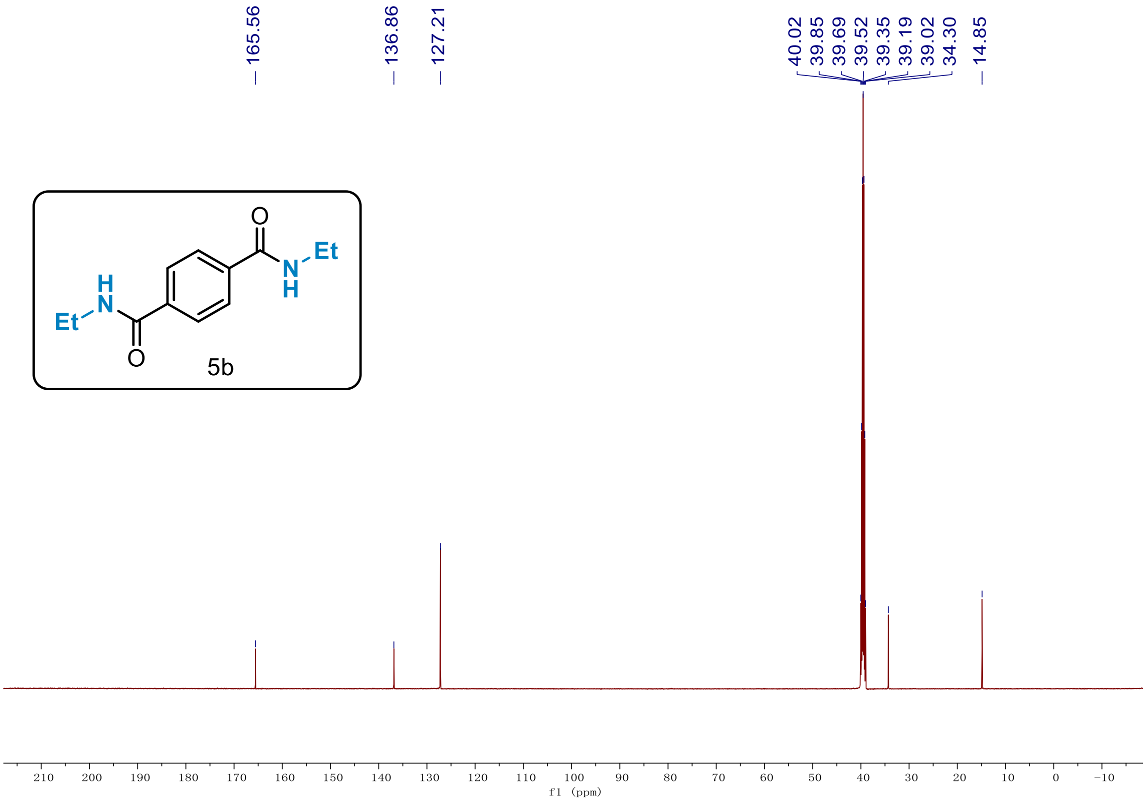

Compound **5c** ^{1}H NMR

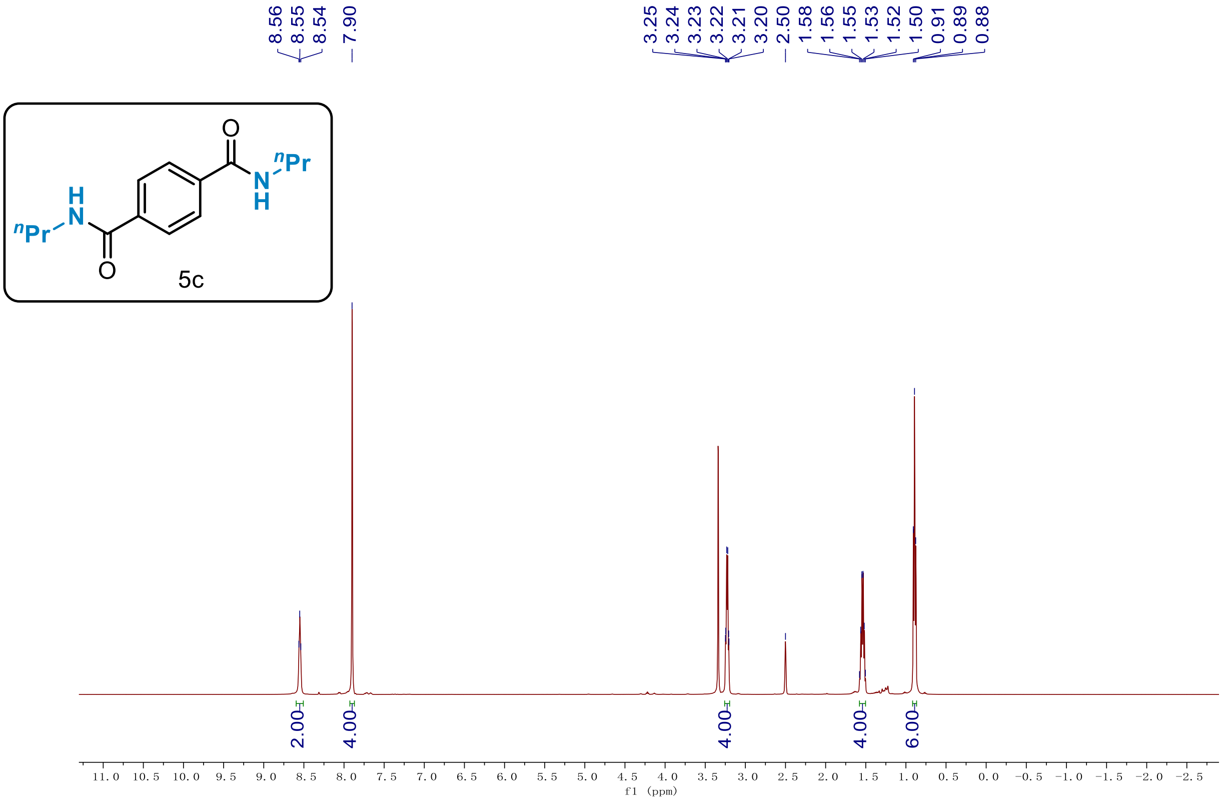

Compound **5c** ^{13}C NMR

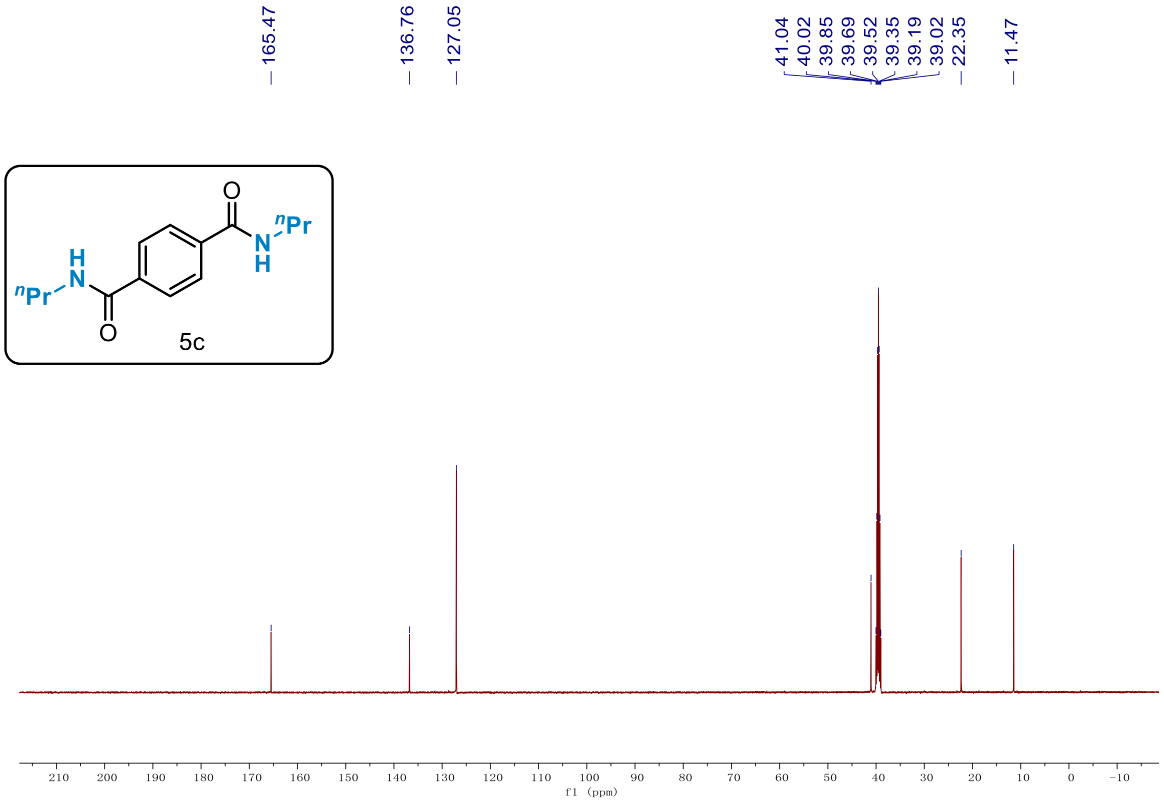

Compound **5d** ^{1}H NMR

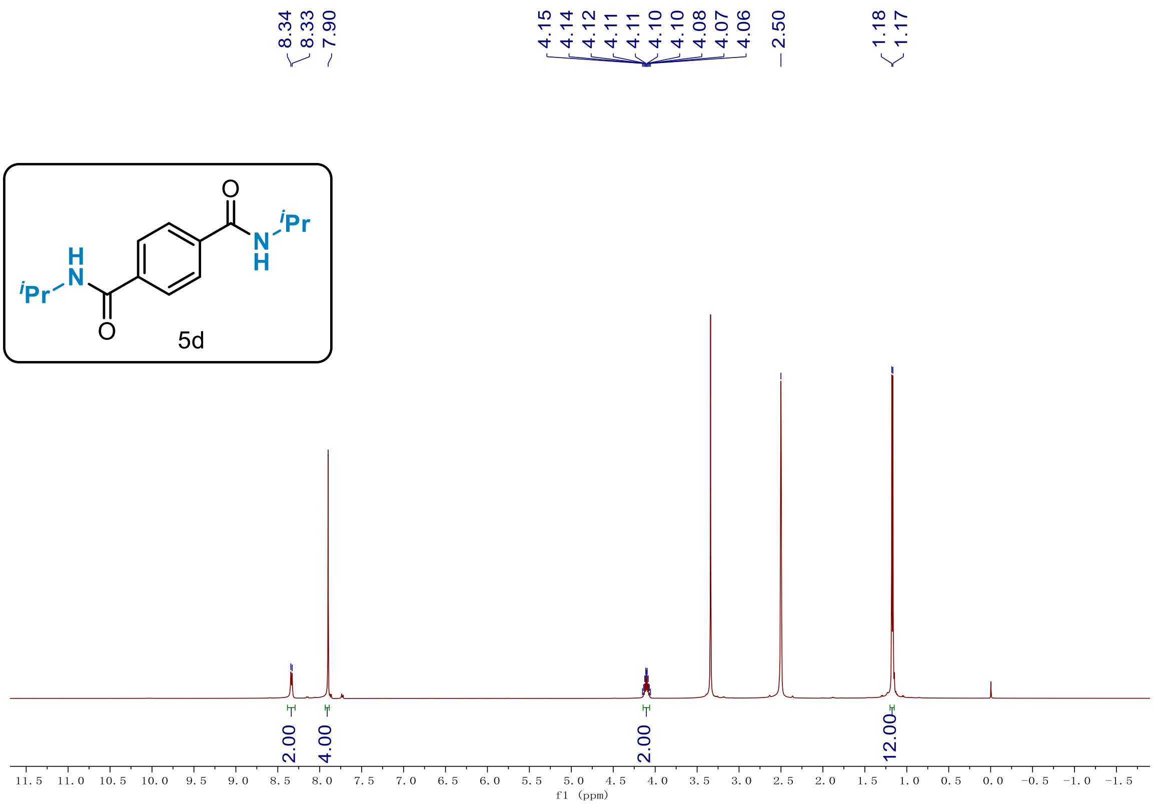

Compound **5d** ^{13}C NMR

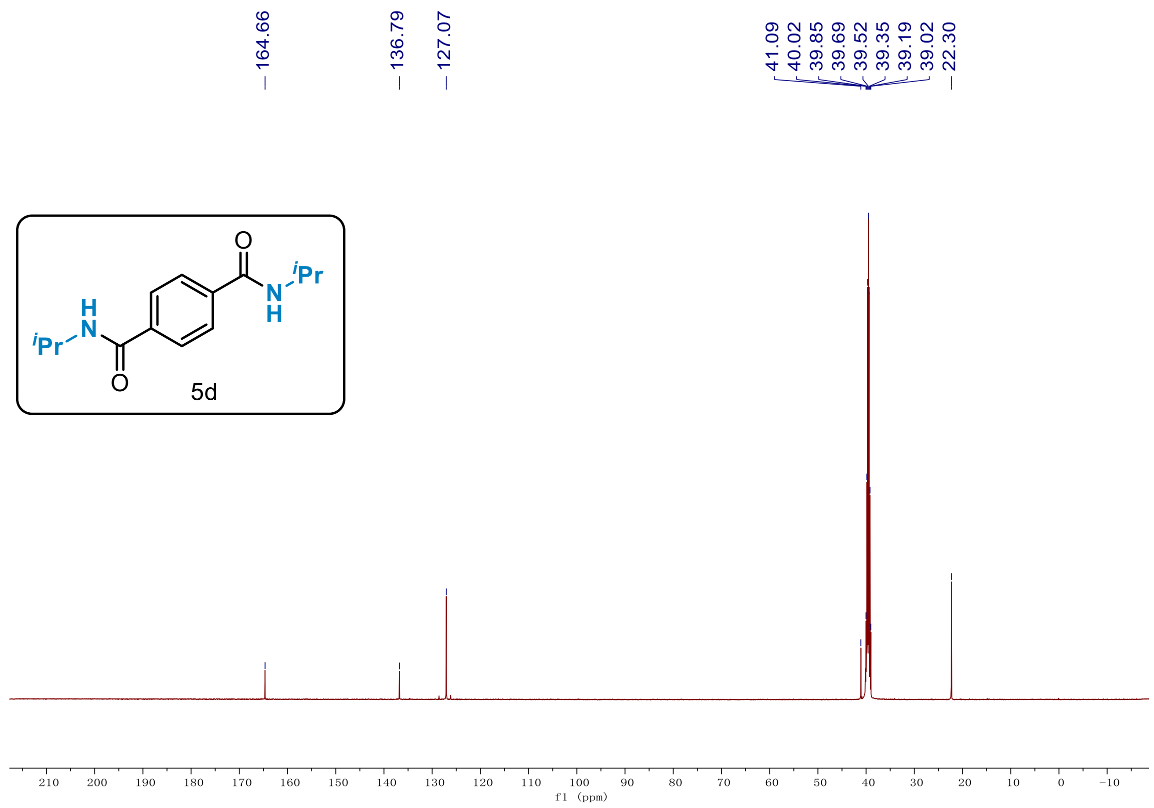

Compound **5e** ^{1}H NMR

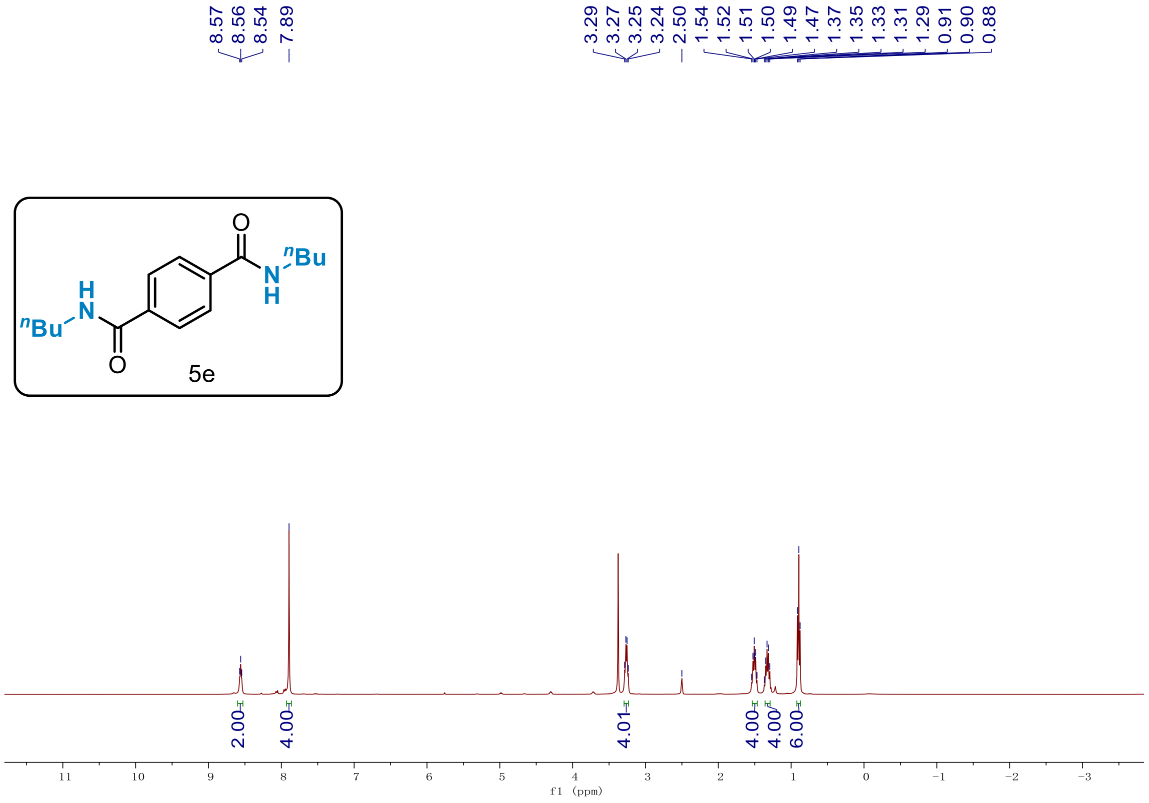

Compound **5e** ^{13}C NMR

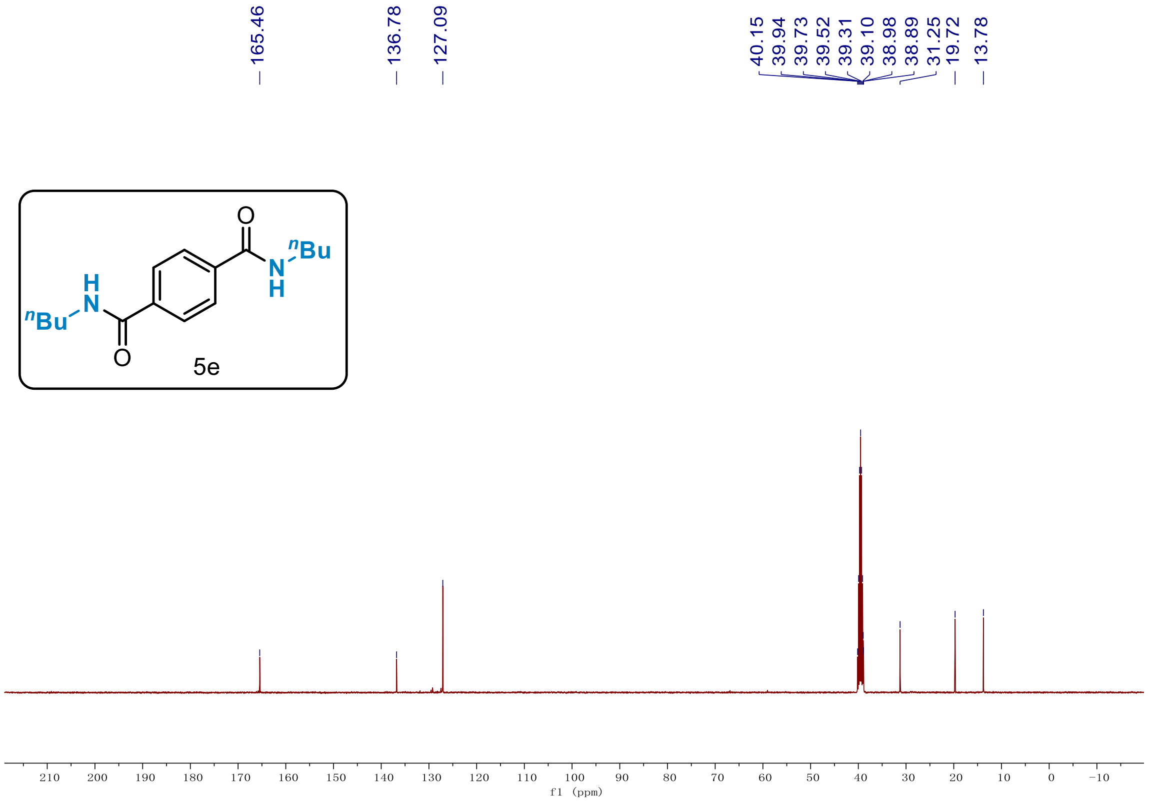

Compound **5f** ^{1}H NMR

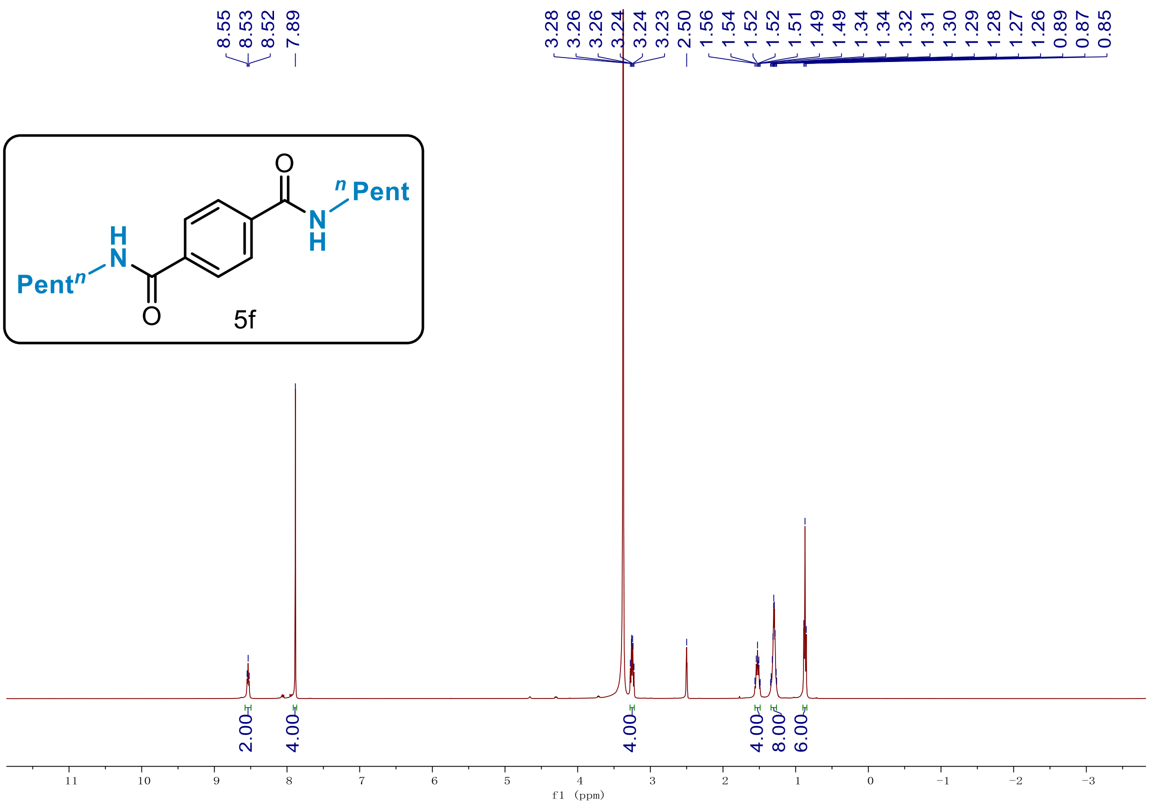

Compound **5f** ^{13}C NMR

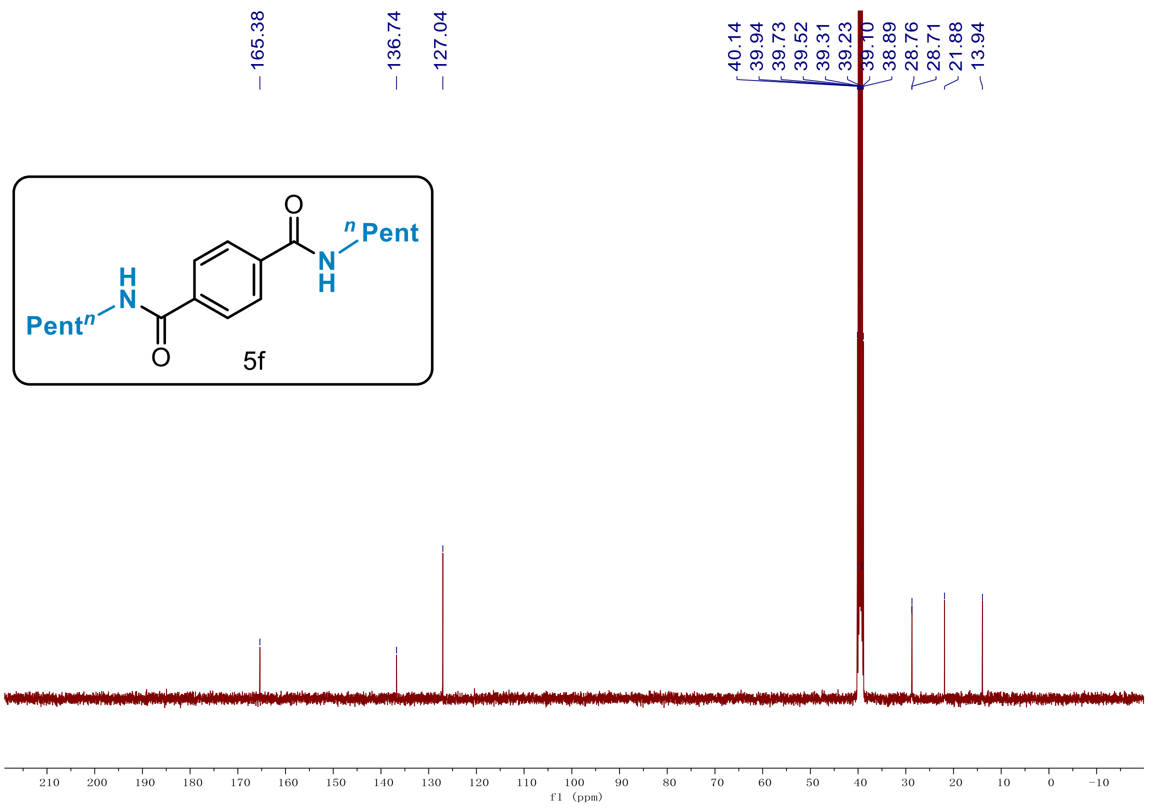

Compound **5g** ^{1}H NMR

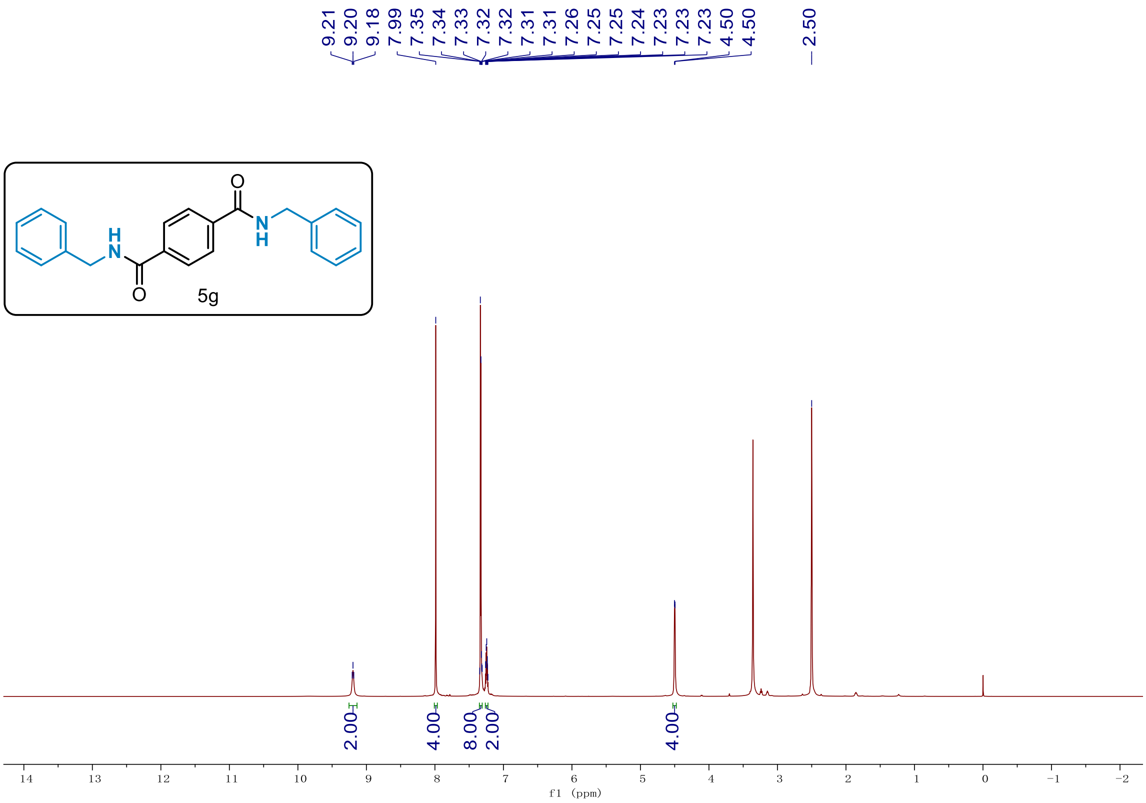

Compound **5g** ^{13}C NMR

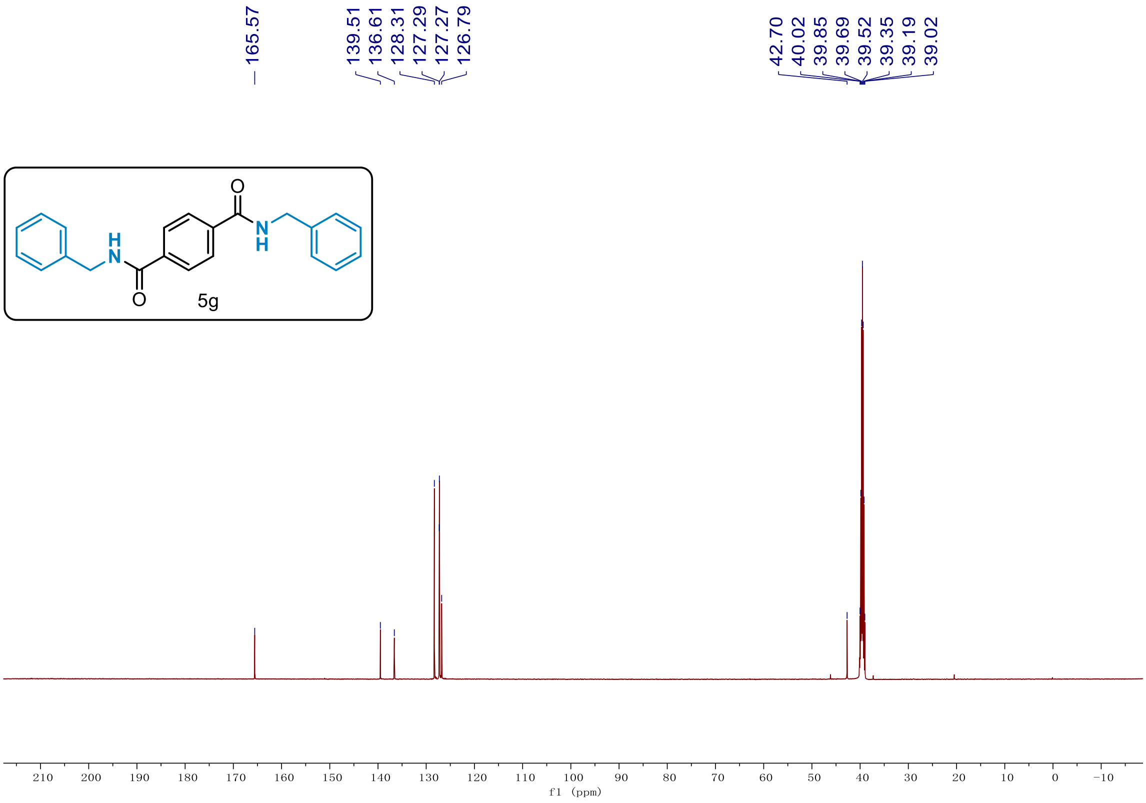

Compound **5h** ^{1}H NMR

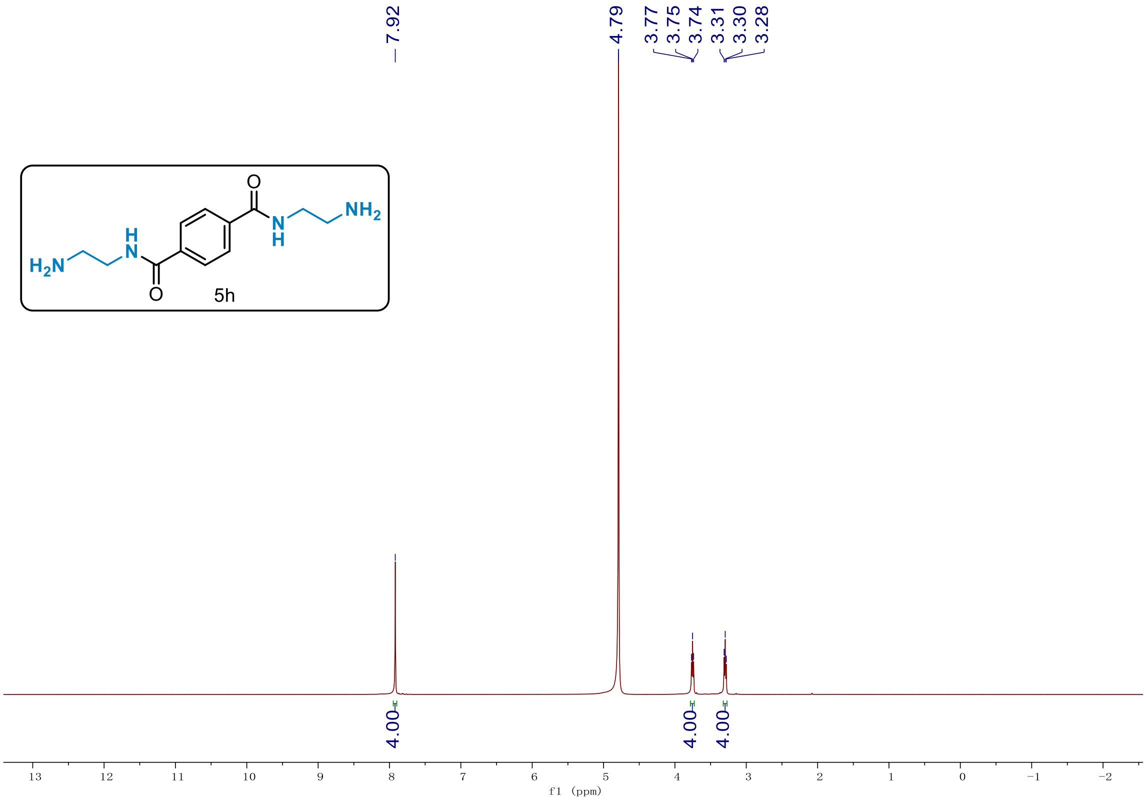

Compound **5h** ^{13}C NMR

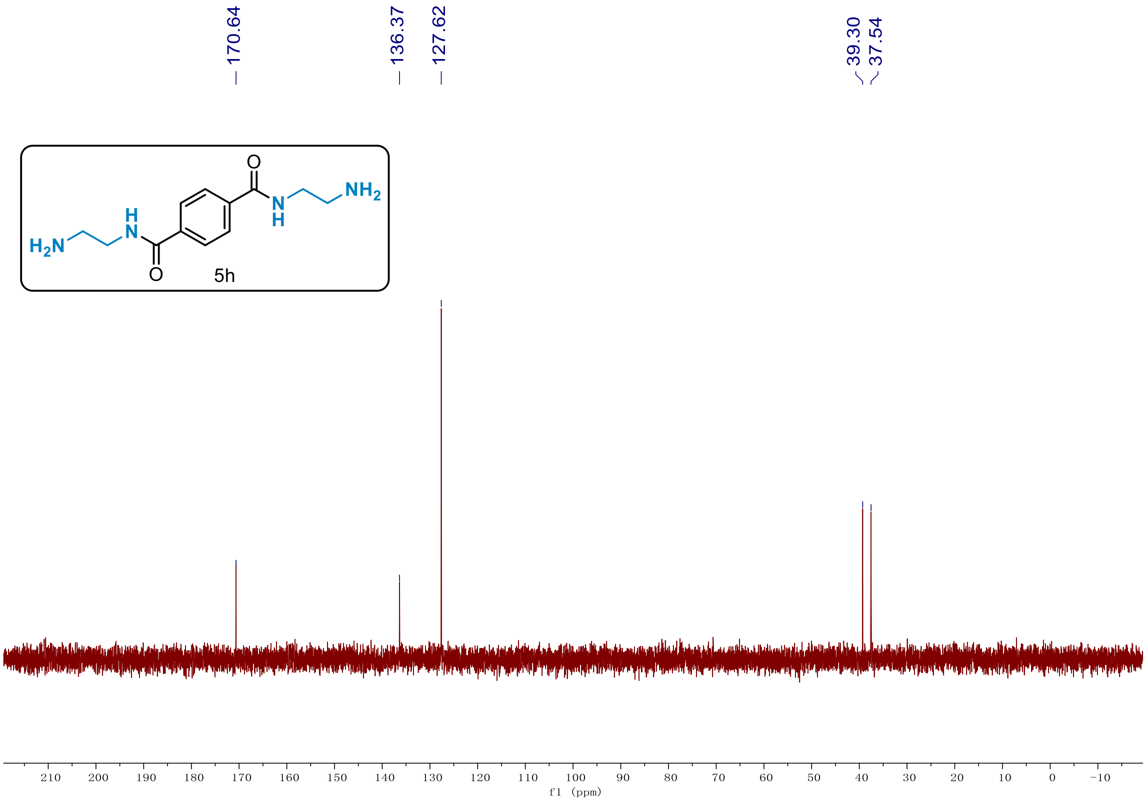

Compound **5i** ^{1}H NMR

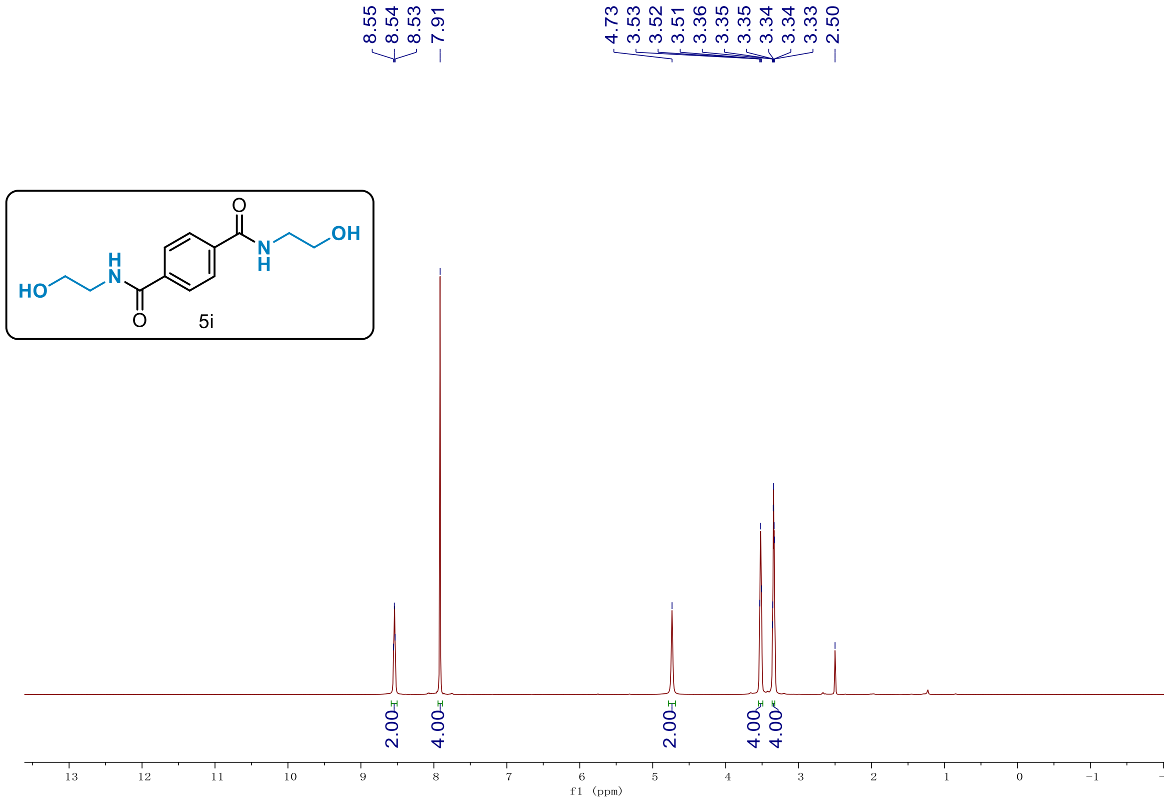

Compound **5i** ^{13}C NMR

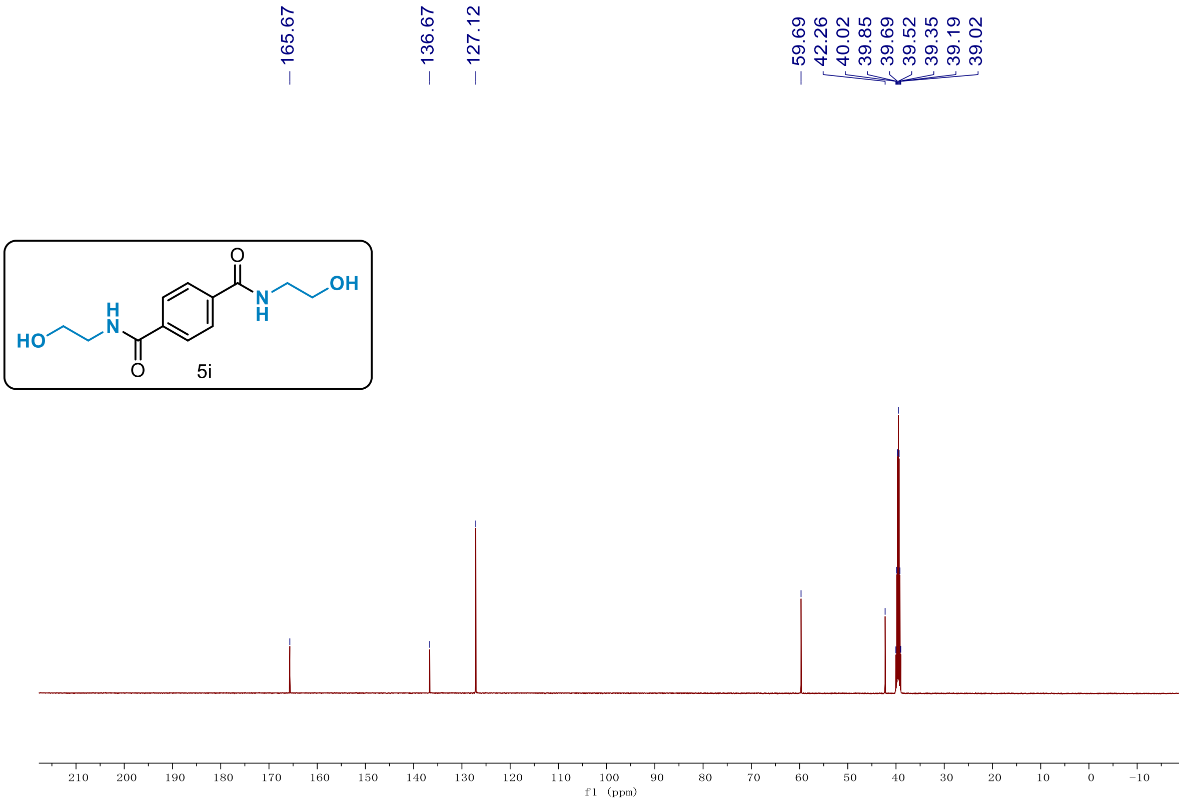

Compound **5j** ^{1}H NMR

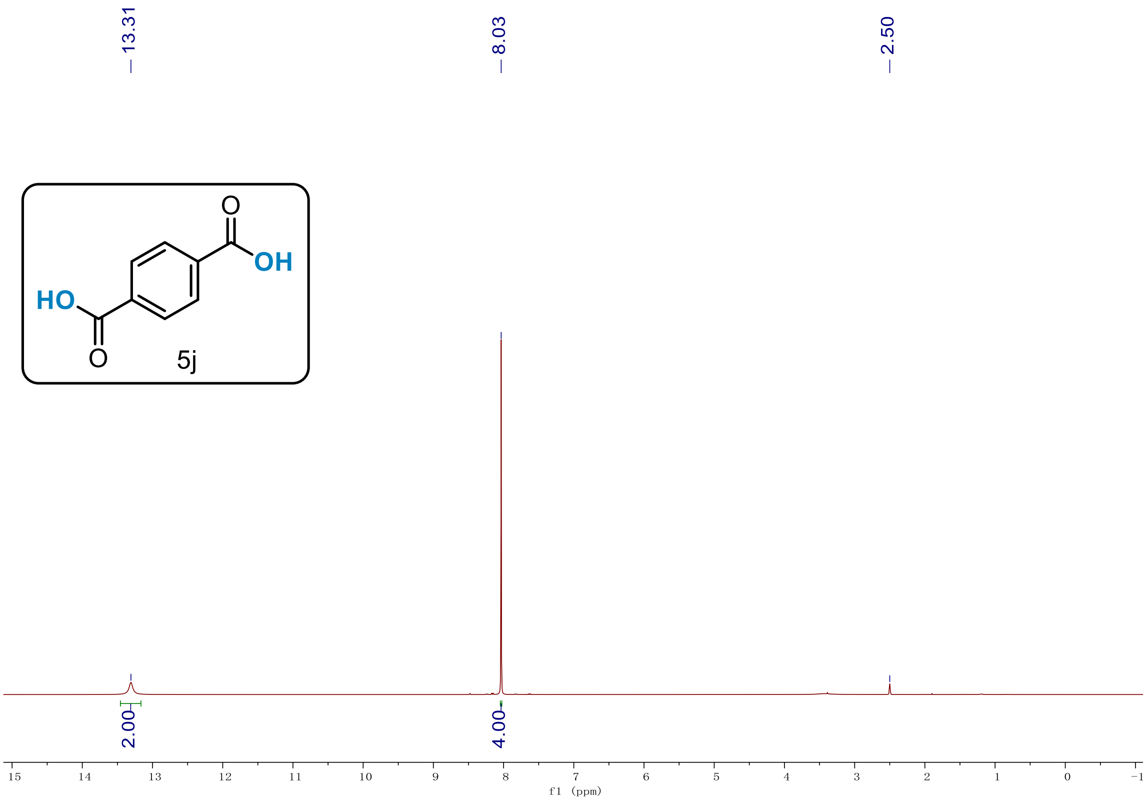

Compound **5j** ^{13}C NMR

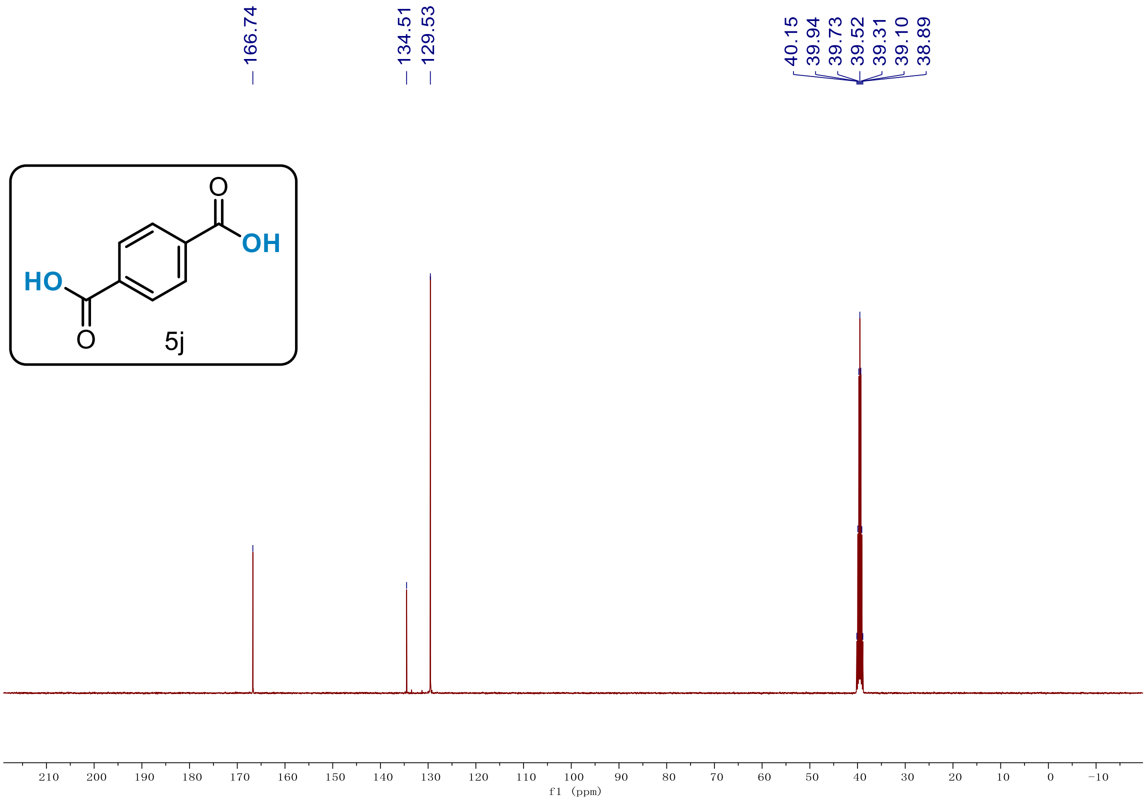

Compound **5k** ^{1}H NMR

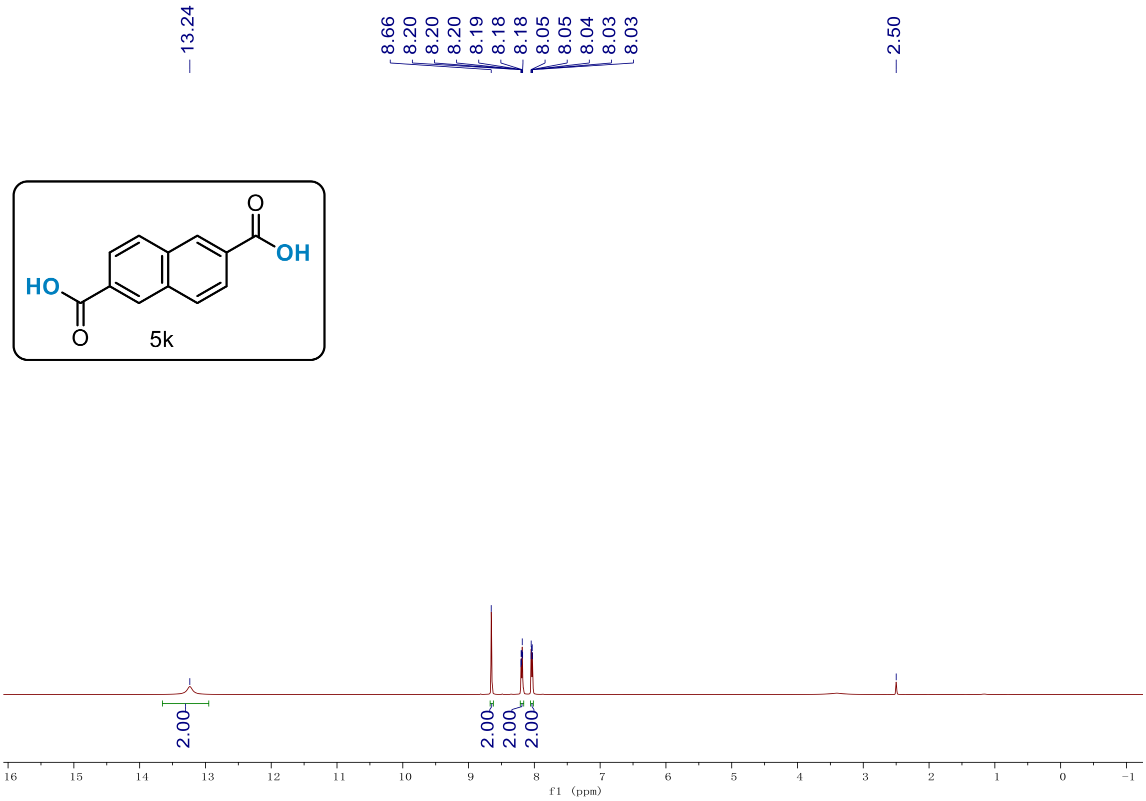

Compound **5k** ^{13}C NMR

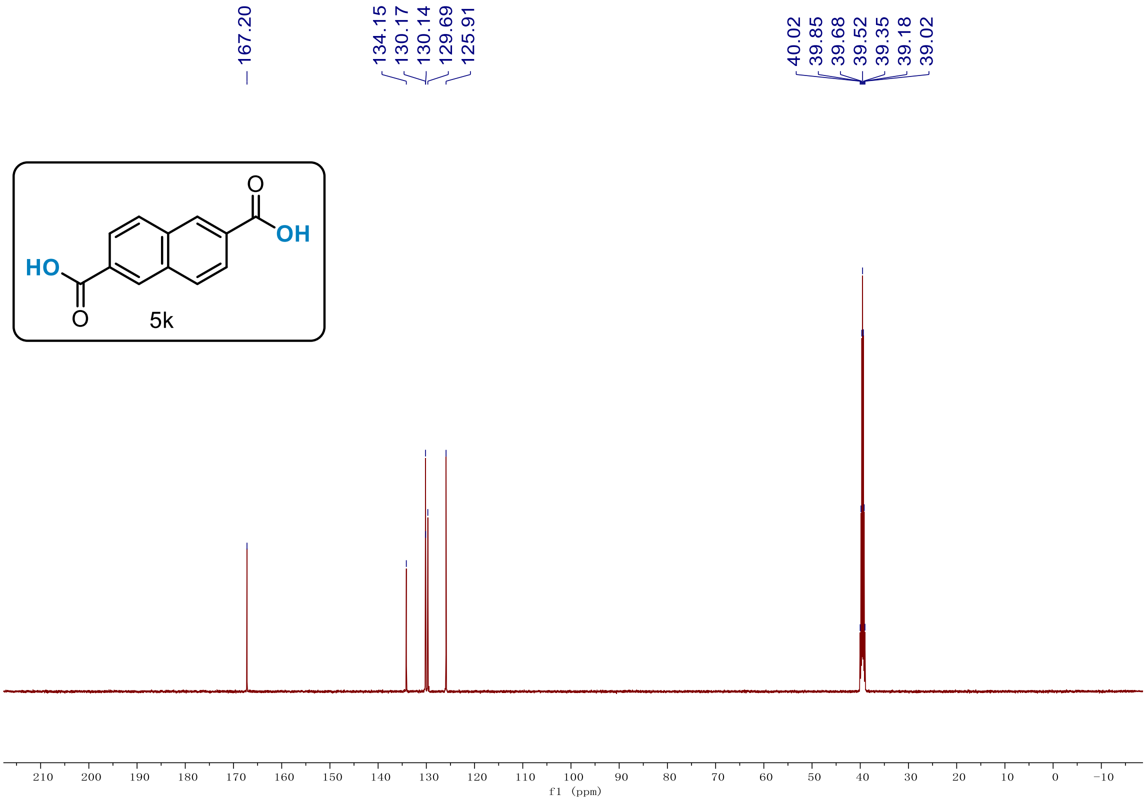

Compound **6a** ^{1}H NMR

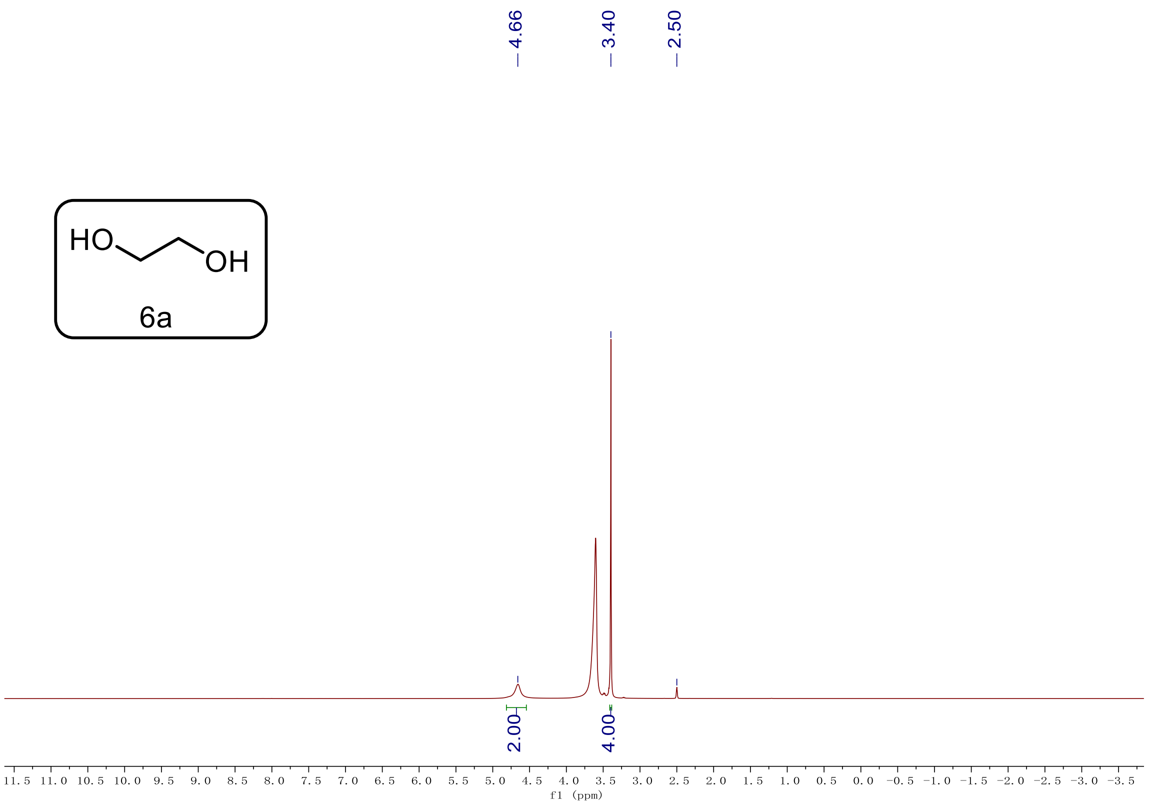

Compound **6a** ^{13}C NMR

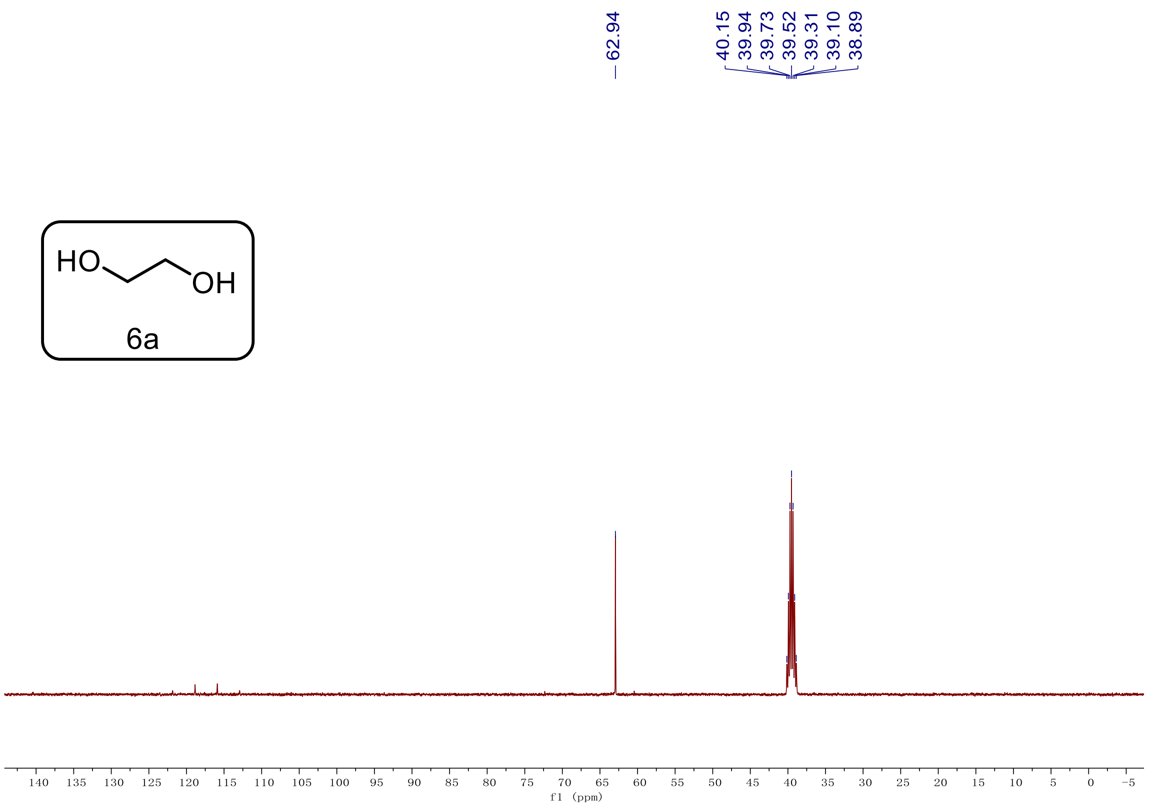

Compound **6b** ^{1}H NMR

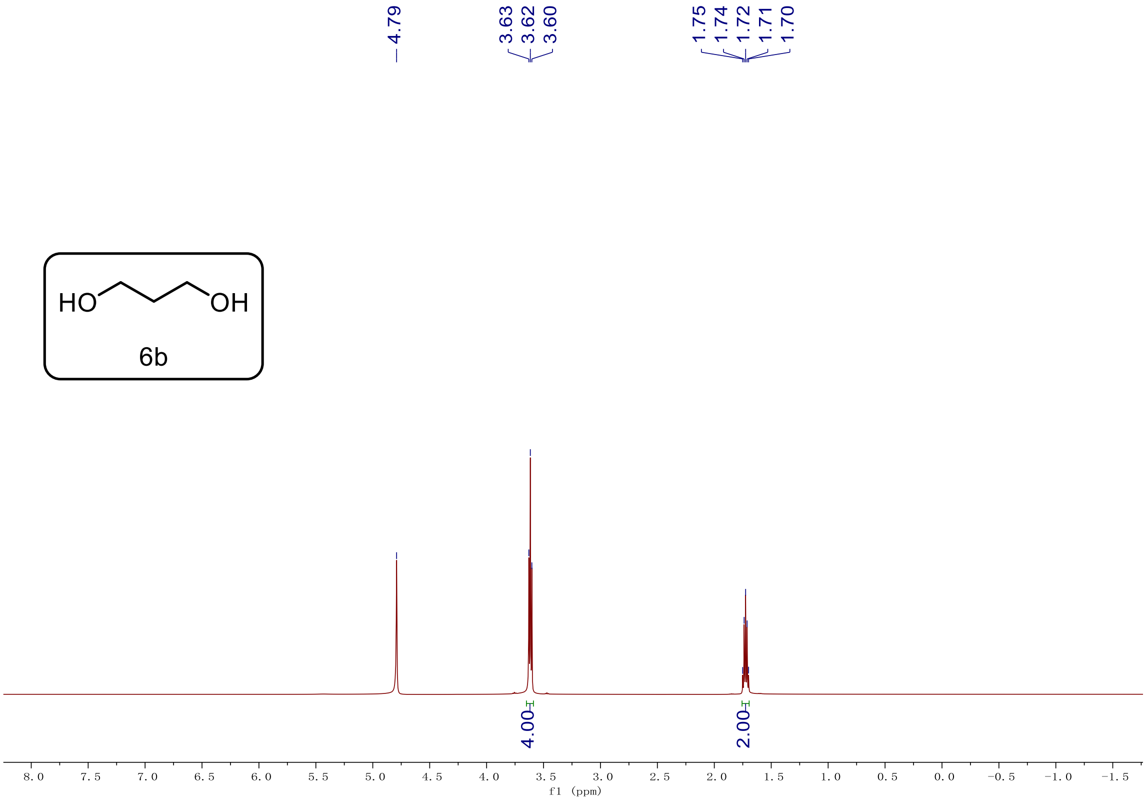

Compound **6b** ^{13}C NMR

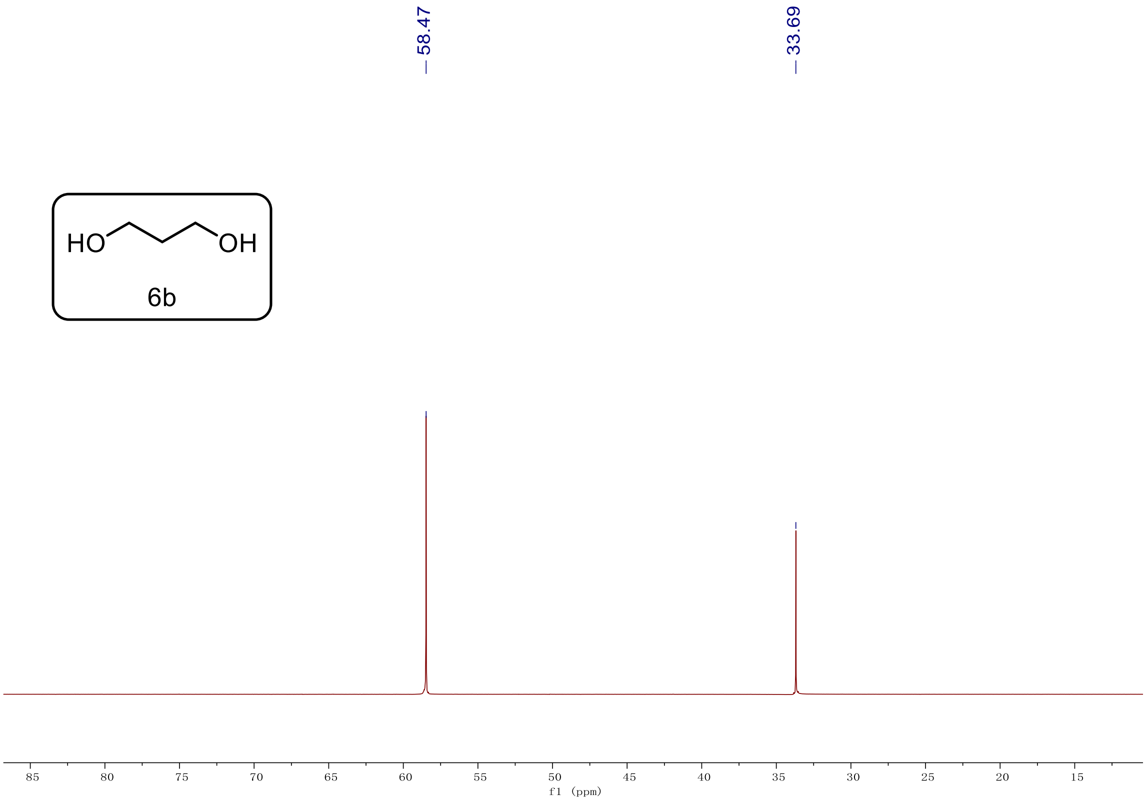

Compound **6c** ^{1}H NMR

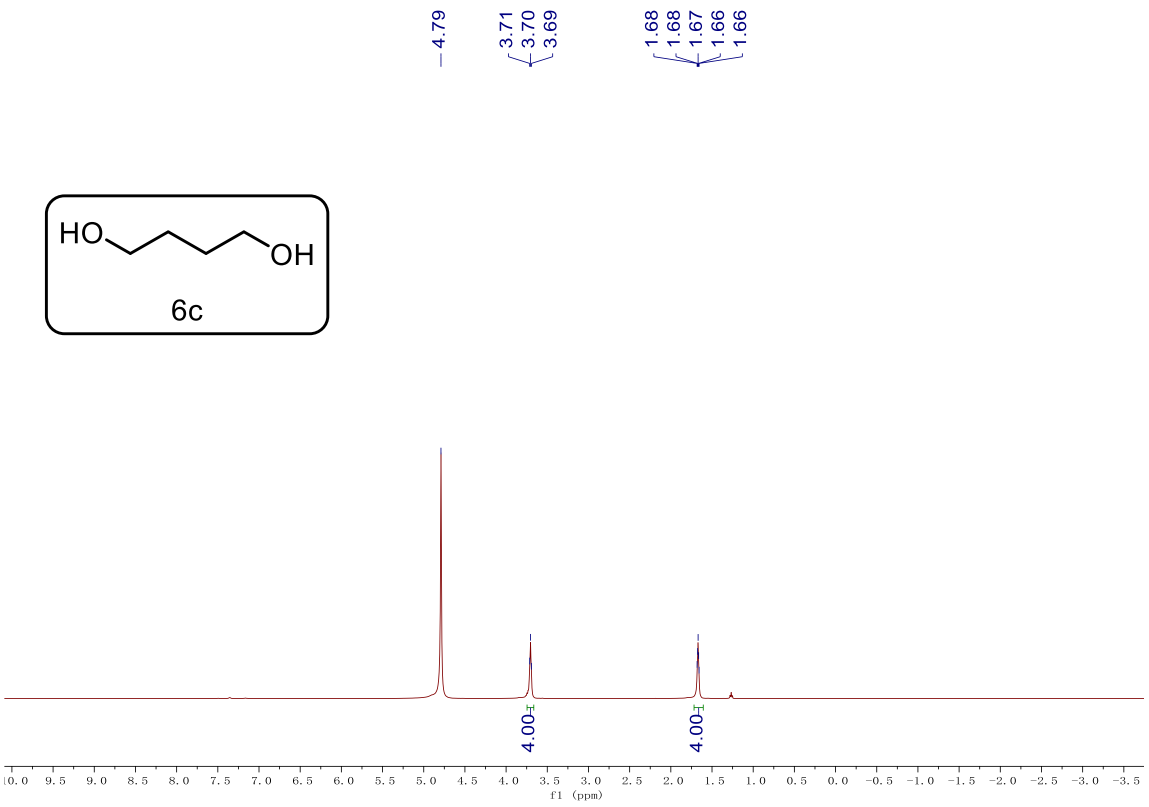

Compound **6c** ^{13}C NMR

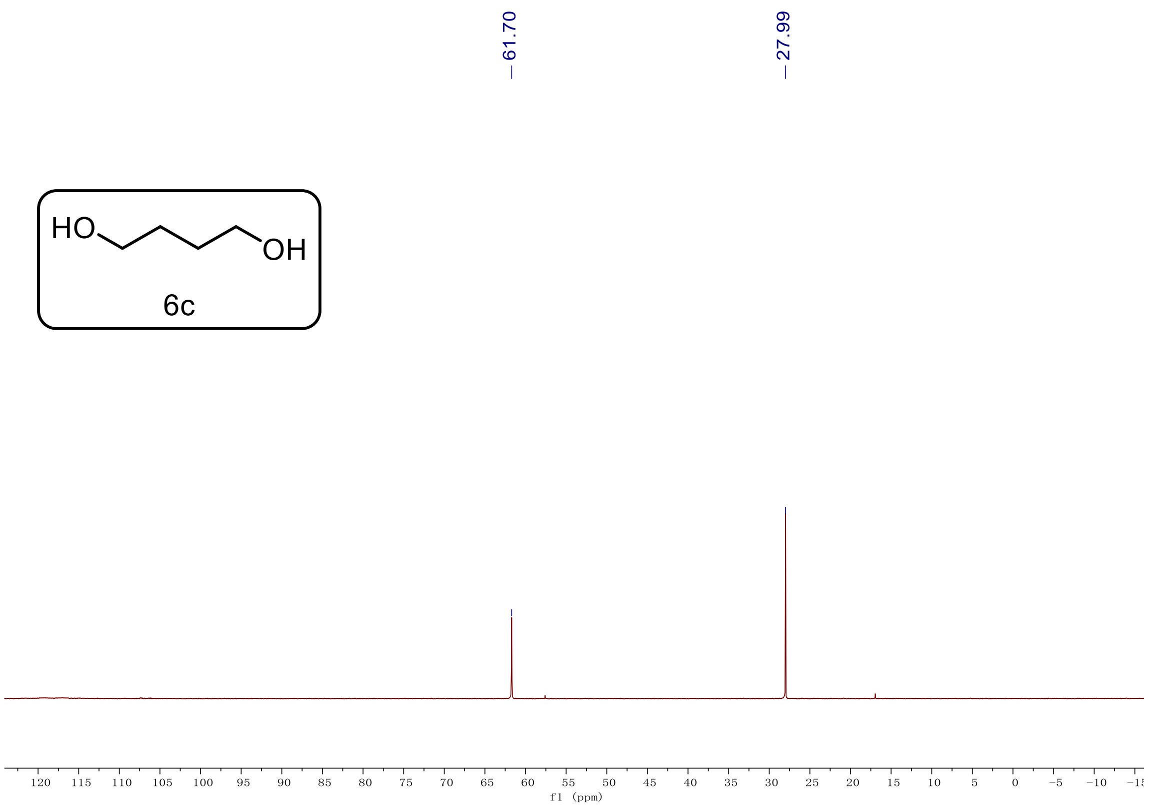

Compound **BPA** ^{1}H NMR

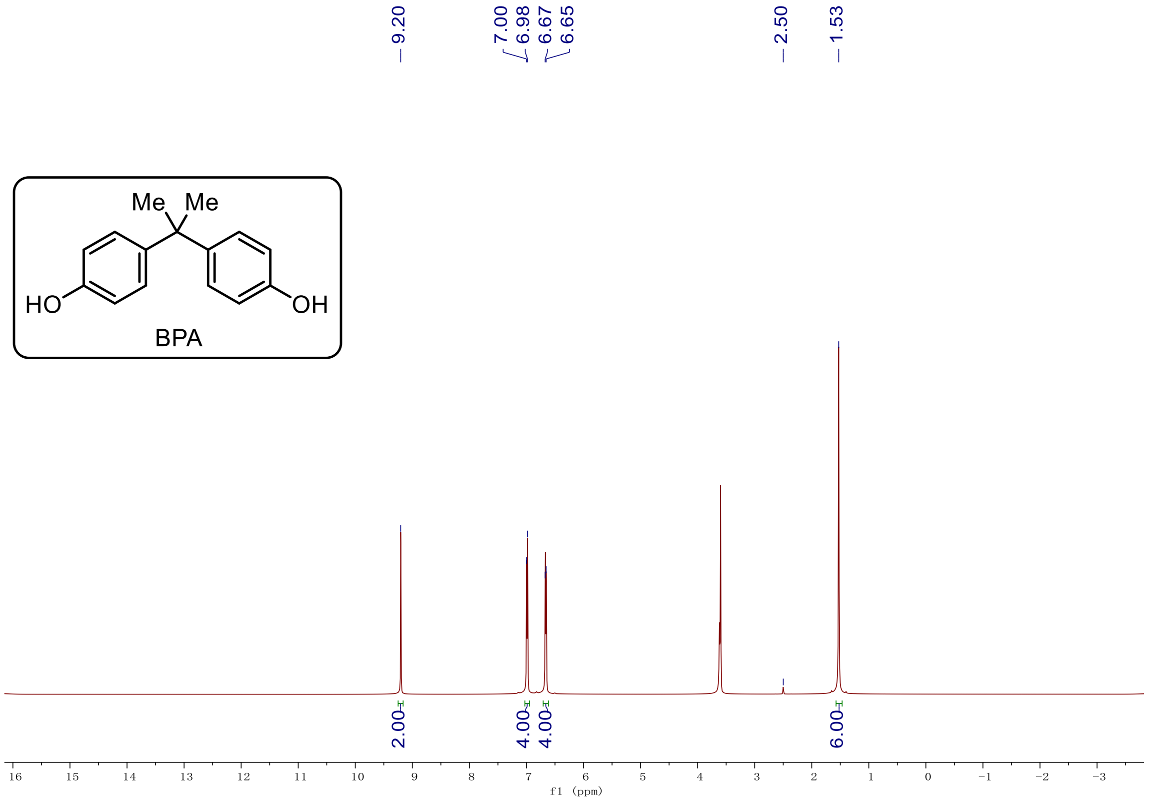

Compound **BPA** ^{13}C NMR

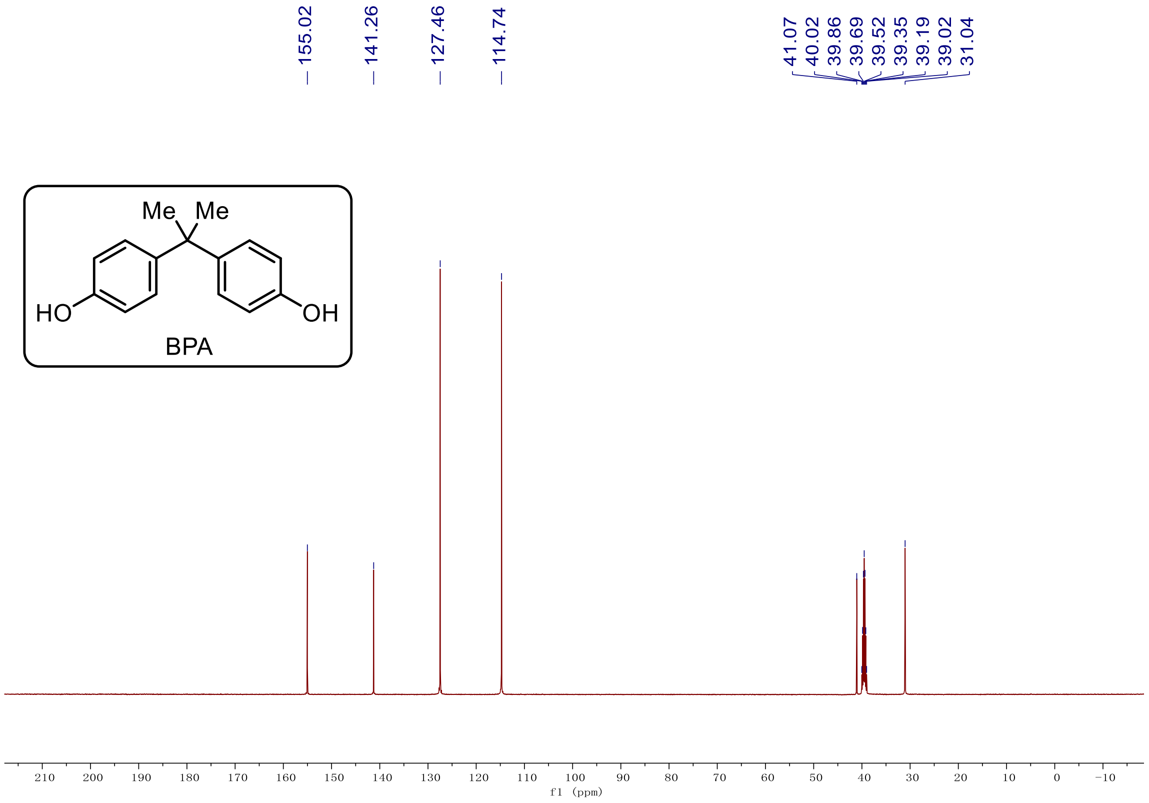

Compound TBD-TFA salt ^{1}H NMR (DMSO-d_6)

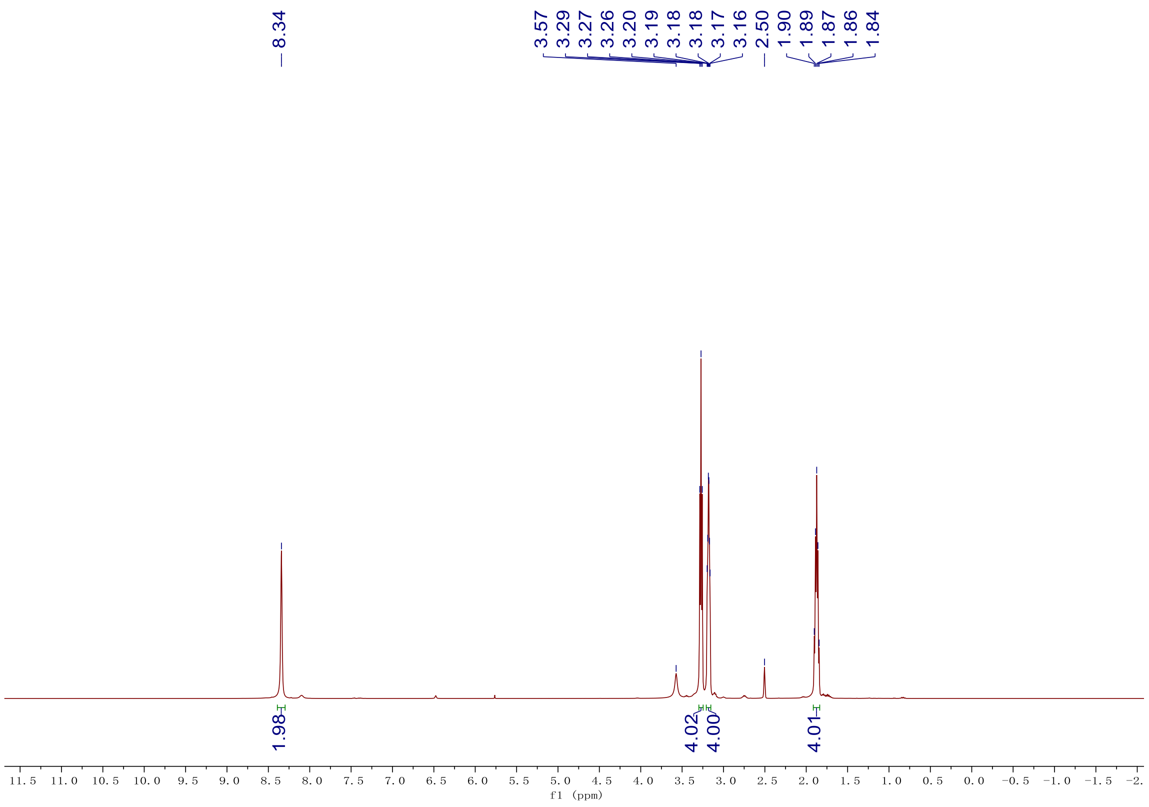

Compound TBD-TFA salt ^{19}F NMR (DMSO-d_6)

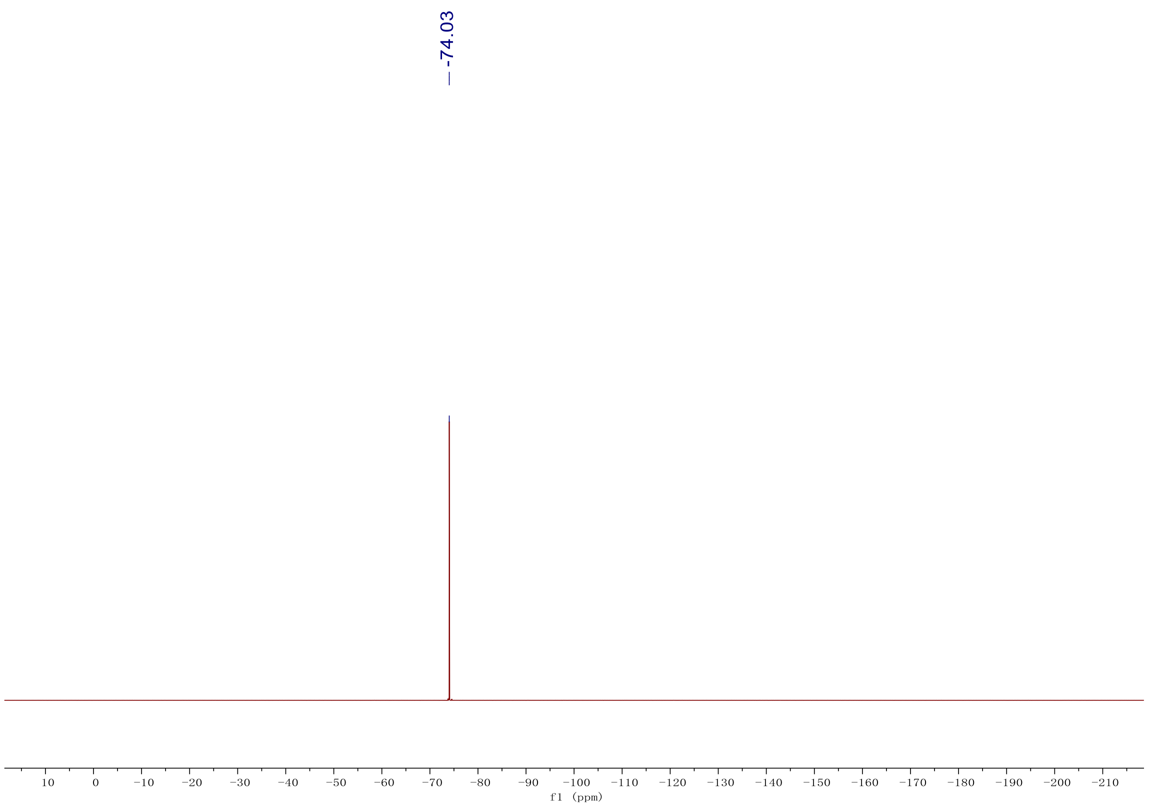

Compound TBD-TFA salt ^{13}C NMR (DMSO-d_6)

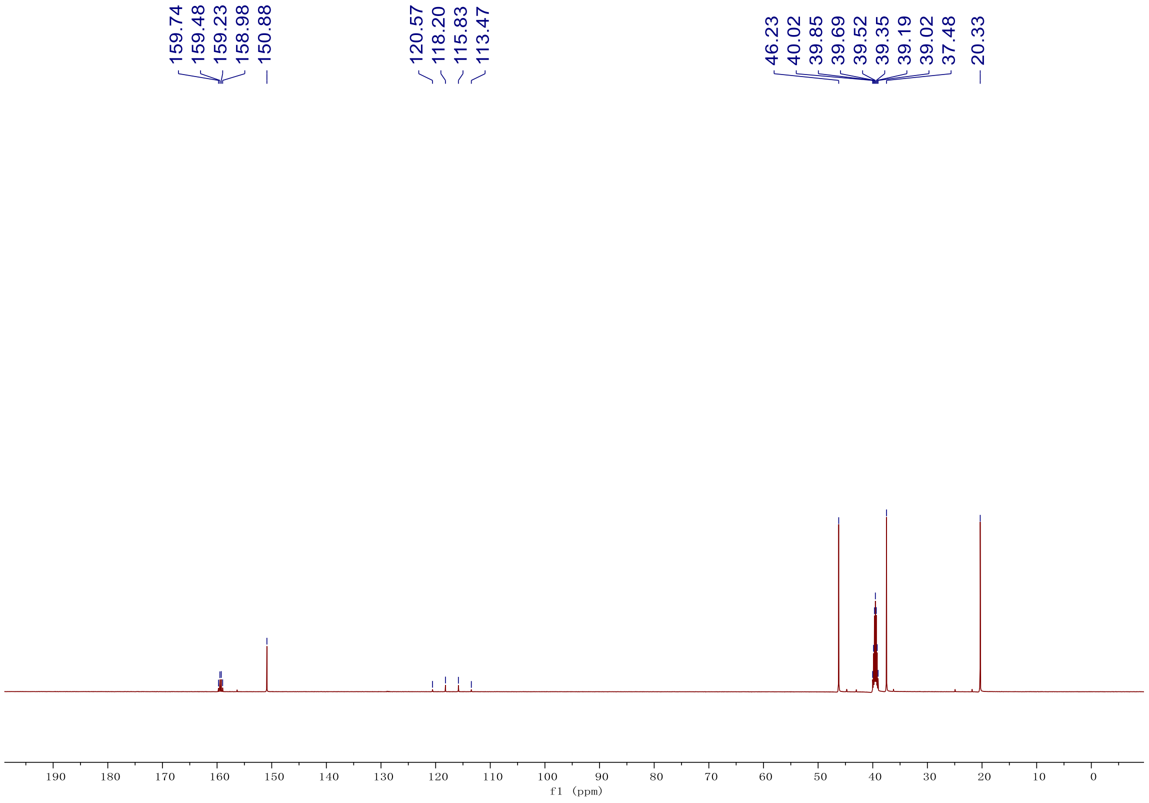

Compound recycled PA_6 ^1H NMR (TFA-d_1).

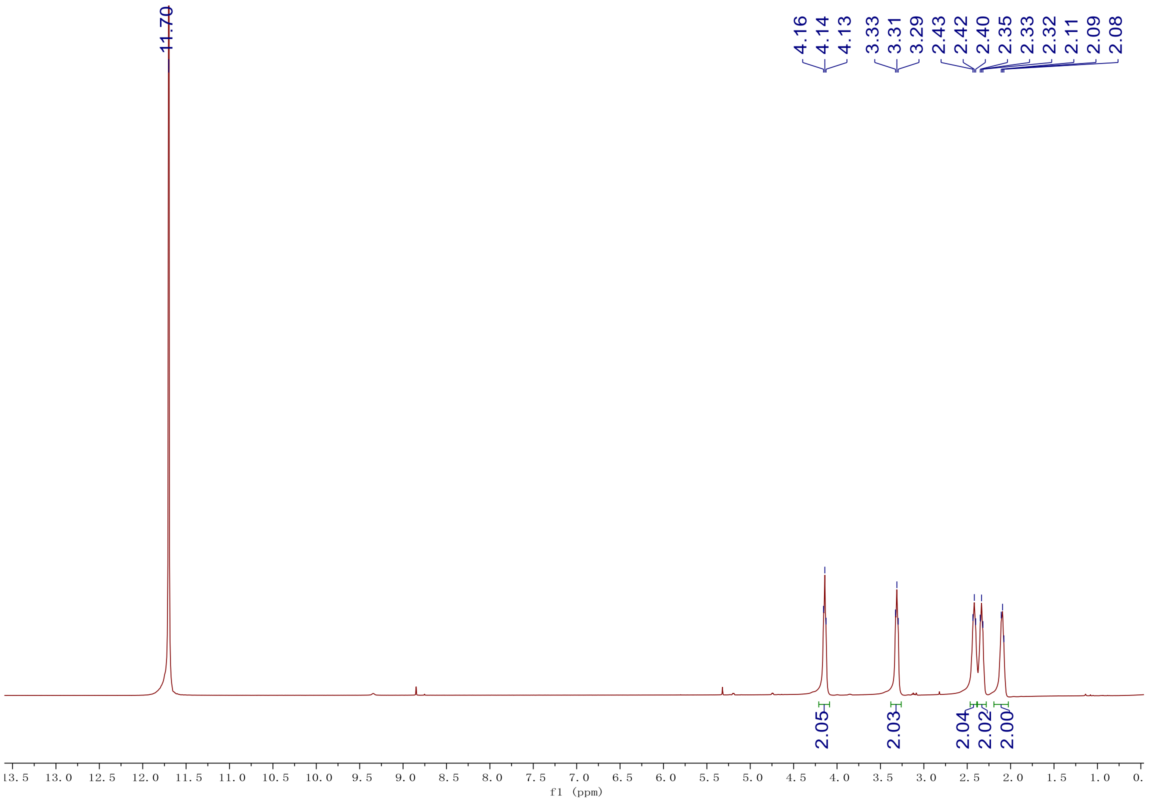

Compound recycled PA_6 ^{13}C NMR (TFA-d_1).

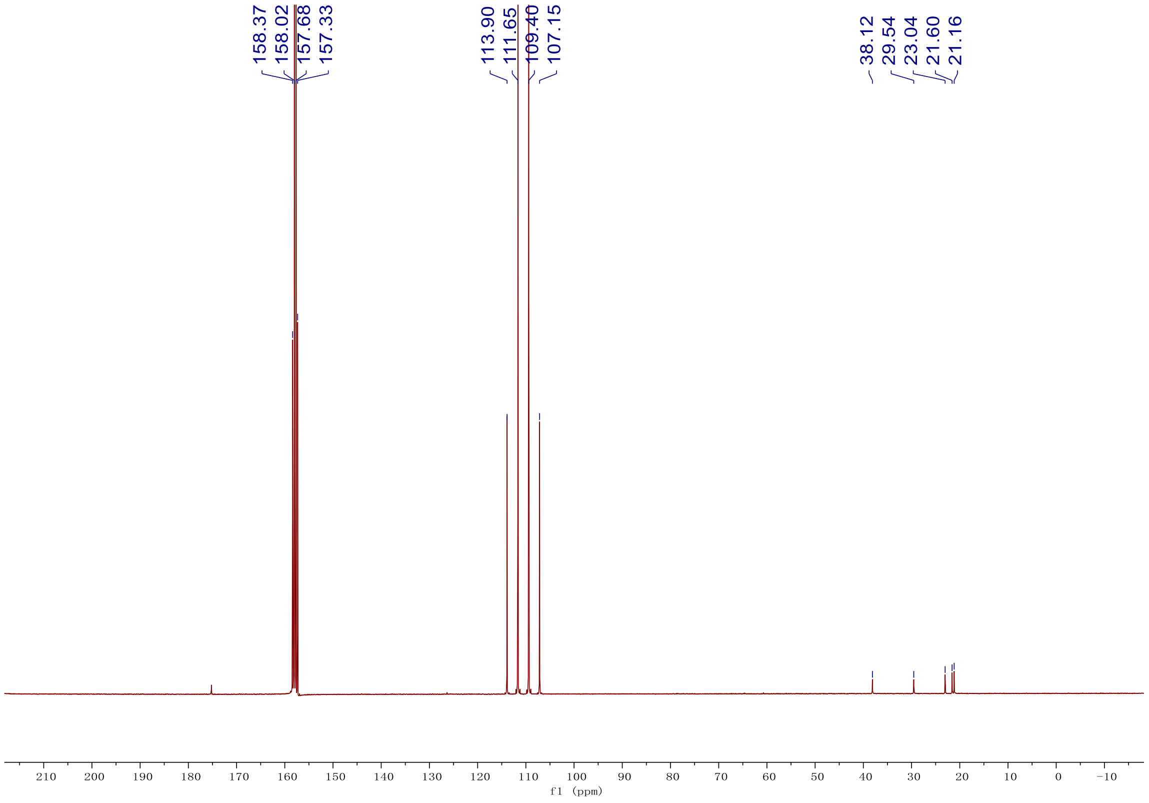

Supplement: Supplementary file 1 — Supporting Information [file ADVS-11-2403002-s001.pdf]
